# Manipulation of topoisomerase expression inhibits cell division but not growth and reveals a distinctive promoter structure in *Synechocystis*

**DOI:** 10.1093/nar/gkac1132

**Published:** 2022-12-19

**Authors:** Anna Behle, Maximilian Dietsch, Louis Goldschmidt, Wandana Murugathas, Lutz C Berwanger, Jonas Burmester, Lun Yao, David Brandt, Tobias Busche, Jörn Kalinowski, Elton P Hudson, Oliver Ebenhöh, Ilka M Axmann, Rainer Machné

**Affiliations:** Institut f. Synthetische Mikrobiologie, Heinrich-Heine Universität Düsseldorf, Universitätsstrasse 1, 40225 Düsseldorf, Germany; Photanol B.V, Science Park 406, 1098 XH Amsterdam, The Netherlands; Institut f. Synthetische Mikrobiologie, Heinrich-Heine Universität Düsseldorf, Universitätsstrasse 1, 40225 Düsseldorf, Germany; Institut f. Quantitative u. Theoretische Biologie, Heinrich-Heine Universität Düsseldorf, Universitätsstrasse 1, 40225 Düsseldorf, Germany; Institut f. Synthetische Mikrobiologie, Heinrich-Heine Universität Düsseldorf, Universitätsstrasse 1, 40225 Düsseldorf, Germany; Institut f. Synthetische Mikrobiologie, Heinrich-Heine Universität Düsseldorf, Universitätsstrasse 1, 40225 Düsseldorf, Germany; Institut f. Synthetische Mikrobiologie, Heinrich-Heine Universität Düsseldorf, Universitätsstrasse 1, 40225 Düsseldorf, Germany; School of Engineering Sciences in Chemistry, Biotechnology and Health, Science for Life Laboratory, KTH – Royal Institute of Technology, Stockholm, Sweden; Centrum für Biotechnologie (CeBiTec), Universität Bielefeld, Universitätsstrasse 27, 33615 Bielefeld, Germany; Centrum für Biotechnologie (CeBiTec), Universität Bielefeld, Universitätsstrasse 27, 33615 Bielefeld, Germany; Centrum für Biotechnologie (CeBiTec), Universität Bielefeld, Universitätsstrasse 27, 33615 Bielefeld, Germany; School of Engineering Sciences in Chemistry, Biotechnology and Health, Science for Life Laboratory, KTH – Royal Institute of Technology, Stockholm, Sweden; Institut f. Quantitative u. Theoretische Biologie, Heinrich-Heine Universität Düsseldorf, Universitätsstrasse 1, 40225 Düsseldorf, Germany; Cluster of Excellence on Plant Sciences (CEPLAS), Heinrich-Heine-Universität Düsseldorf, Universitätsstraße 1, 40225 Düsseldorf, Germany; Institut f. Synthetische Mikrobiologie, Heinrich-Heine Universität Düsseldorf, Universitätsstrasse 1, 40225 Düsseldorf, Germany; Institut f. Synthetische Mikrobiologie, Heinrich-Heine Universität Düsseldorf, Universitätsstrasse 1, 40225 Düsseldorf, Germany; Institut f. Quantitative u. Theoretische Biologie, Heinrich-Heine Universität Düsseldorf, Universitätsstrasse 1, 40225 Düsseldorf, Germany

## Abstract

In cyanobacteria DNA supercoiling varies over the diurnal cycle and is integrated with temporal programs of transcription and replication. We manipulated DNA supercoiling in *Synechocystis* sp. PCC 6803 by CRISPRi-based knockdown of gyrase subunits and overexpression of topoisomerase I (TopoI). Cell division was blocked but cell growth continued in all strains. The small endogenous plasmids were only transiently relaxed, then became strongly supercoiled in the TopoI overexpression strain. Transcript abundances showed a pronounced 5’/3’ gradient along transcription units, incl. the rRNA genes, in the gyrase knockdown strains. These observations are consistent with the basic tenets of the homeostasis and twin-domain models of supercoiling in bacteria. TopoI induction initially led to downregulation of G+C-rich and upregulation of A+T-rich genes. The transcriptional response quickly bifurcated into six groups which overlap with diurnally co-expressed gene groups. Each group shows distinct deviations from a common core promoter structure, where helically phased A-tracts are in phase with the transcription start site. Together, our data show that major co-expression groups (regulons) in *Synechocystis* all respond differentially to DNA supercoiling, and suggest to re-evaluate the long-standing question of the role of A-tracts in bacterial promoters.

## INTRODUCTION

**Figure 1.**
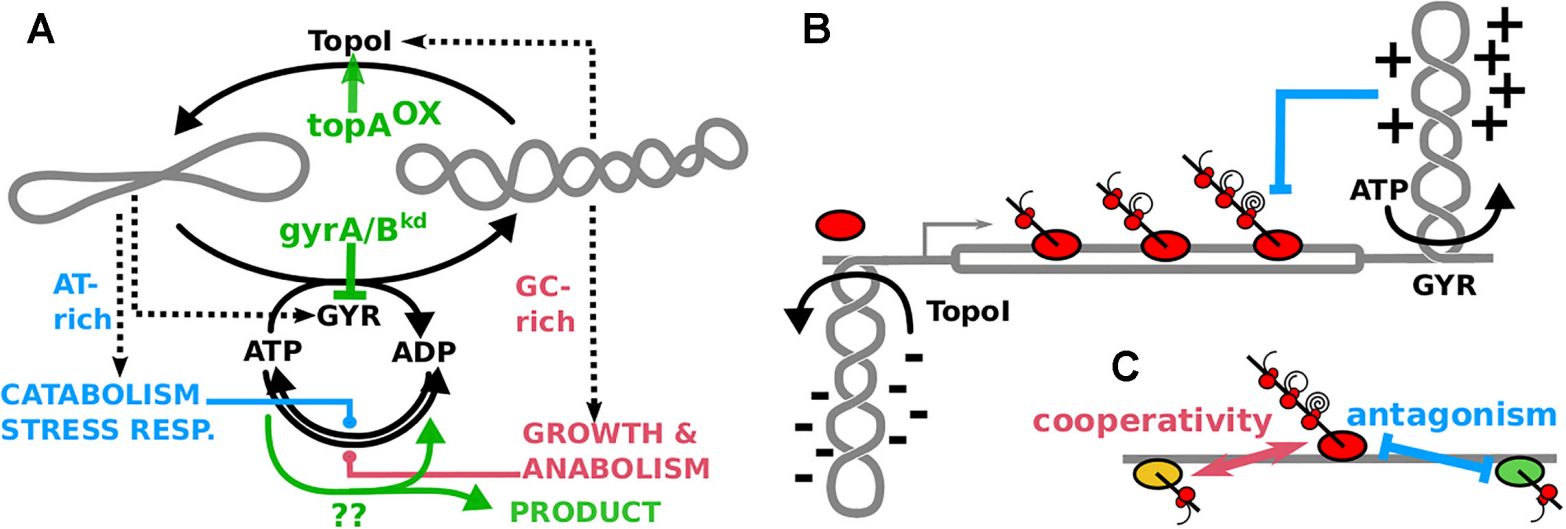
Homeostasis and *Twin-Domain* Models of DNA Supercoiling. (**A**) Global homeostasis of supercoiling by direct feedback on the expression of topoisomerases (GYR: Gyrase holoenzyme; TopoI: topoisomerase I) and G+C-rich anabolic/growth genes and A+T-rich catabolic and stress-response genes. The gray coils reflect relaxed (left) or supercoiled DNA (right). Dashed arrows indicate transcription and solid arrows catalytic conversions. Green arrows indicate the manipulations of this core regulatory hub studied in this work and the underlying hypothesis that these could be used to redirect metabolic energy towards desired products. (**B**) Transcription-dependent DNA supercoiling accumulates downstream (positive) and upstream (negative) of the RNA polymerase, widely known as the *twin-domain* model. If unresolved by TopoI and gyrase, this would lead to RNA polymerase stalling (blue arrow) and R-loop formation. (**C**) The torsional stress exerted by transcription can lead to long-distance cooperative and antagonistic effects, where negative supercoiling upstream facilitates and positive supercoiling downstream blocks transcription from adjacent loci.

*In vivo*, the DNA double helix exists in a torsionally strained and underwound state, often denoted as ‘negative DNA supercoiling’. In bacteria, a homeostatic feedback system of DNA supercoiling is coupled to differential expression of large gene groups. Supercoiling is high during times of high metabolic flux, such as during exponential growth, and is required to express rRNA and G+C-rich growth-related genes and for DNA replication (1). Supercoiling arises as a consequence of DNA transcription and replication and is regulated by two enzymes: gyrase, a heterotetramer of *gyrA* and *gyrB* gene products, can remove positive supercoiling and introduce negative supercoiling, using energy from ATP hydrolysis; and topoisomerase I (TopoI, gene: *topA*) can remove negative supercoiling without any co-factors. The transcription of both enzymes is itself regulated by supercoiling-sensitive promoters in a negative feedback, leading to a homeostatic control of supercoiling (2–6). The ATP dependence of gyrase (7–9) and the control over the expression of growth-related (rRNA, ribosomal proteins, biosynthesis) and G+C-rich genes, and catabolism-related and A+T-rich genes (10–16) extends this homeostatic system to metabolism (Figure 1A).

However, the relation between DNA transcription and replication to supercoiling is mutual and complex (1). According to the twin-domain model (17) of transcription-dependent supercoiling (Figure 1B), negative supercoiling accumulates upstream and positive supercoiling downstream of RNA polymerases, leading to cooperative and antagonistic long-range effects between transcription loci (18) (Figure 1C). Strong transcriptional activity requires downstream activity of gyrase to set the elongation rate and avoid RNA polymerase stalling (19–21) and upstream activity of TopoI to avoid R-loop formation and genome instability (22,23). Such cooperative long-range effects can underpin temporal expression programs; locally in the *leu* operon (24,25) and globally as a spatio-temporal gradient along the origin-terminus axis of the *Escherichia coli* genome (26). The DNA sequence properties of a short region (discriminator) just upstream of the transcription start site are suspected to underlie the common response of many promoters to both supercoiling and to guanosine tetraphosphate (ppGpp) (27–35). Additionally, bacterial and bacteriophage promoters often show a pattern of short repeats of A and T nucleotides (A-tracts) upstream of the core promoter, repeated at distances that match the pitch of the DNA helix (helically phased) (5,36–46), e.g. in light-responsive genes of cyanobacteria (43). On a genome-wide scale (47–54) the helically phased enrichment of A-tract-related dinucleotide motifs is especially pronounced in genomes of polyploid cyanobacteria, including *Synechocystis* sp. PCC6803 (hereafter: *Synechocystis*), where it is found in both intergenic and protein-coding regions (53).

In cyanobacteria and chloroplasts (descendants of cyanobacteria) supercoiling fluctuates with the light/dark (LD) cycle (55,56), and supercoiling homeostasis is integrated with the transcriptional output of the cyanobacterial circadian clock in *Synechococcus elongatus* PCC 7942 (*S. elongatus*) (12,57,58). Recently, the focus has shifted towards the role of ppGpp (59), in dark-phase transcription shutdown (60) and light-phase modulation of the diurnal transcription program (61) of *S. elongatus*, but ppGpp and supercoiling affect the same type of promoters in the same direction in many species (32). In *Synechocystis*, a cold-shock induced increase in supercoiling was found to affect fatty acid synthesis (62), and the transcriptome response to the gyrase inhibitor novobiocin (NB) largely overlapped with the response to various stress conditions (13).

Its central position in metabolism- and growth-related transcription suggests supercoiling as a biotechnological target, where an artificial de-construction and re-construction of this homeostatic feedback may allow to control cellular resource allocation and channel metabolic energy into desired products. Here, we tested the current models of supercoiling in *Synechocystis*, also with respect to this biotechnological potential (Figure 1A). Overexpression of *topA* (63) and CRISPRi-based knockdown of gyrase subunits (64) induced a pleiotropic phenotype, where cell division was blocked but cell volume growth continued. Glycogen and ATP+ADP content increased only upon *topA* overexpression. The transcriptome changed quickly and globally upon induction, compatible with the global homeostatic model (Figure 1A), then remained locked in a state reflecting the dark/light transition at dawn. A graded response at rRNA loci and growth-related transcription units in gyrase knockdown strains is compatible with the twin-domain model (Figure 1B, C), where gyrase activity downstream of RNA polymerase facilitates strong transcription. Coexpressed groups of transcription units show significant deviations from a common core promoter structure.

## MATERIALS AND METHODS

### Strains and plasmids

The *Synechocystis* parental strain used for all genetic alterations is described by Yao *et al.* (64) and contains an CRISPRi-based gene knockdown system where both the dCas9 protein and the sgRNA expression are inducible by anhydrotetracycline (aTc). All strains further contained our pSNDY plasmid for rhamnose-inducible overexpression (63). Supplementary Table S1 provides details on strain construction and plasmid design. The sgRNA protospacer sequences (Supplementary Table S2) were designed with CHOPCHOP (65), and potential off-targets were predicted after Cui *et al.* (66).

### Batch culture conditions

For pre-culturing, growth and endpoint experiments, strains were cultivated in BG11 medium (67) in 100 ml Erlenmeyer flasks in an Infors HT multitron chamber, at 30°C with 150 rpm shaking, continuous illumination of ≈80 μmol m^−2^ m^−1^ and CO_2_ enriched air (0.5%). Pre-culturing was performed in 20 ml cell suspension for three days. For experiments, cultures were adjusted to OD_750_ ≈ 0.4 in 30 ml, grown for one additional day, then density was again adjusted to a start OD_750_ ≈0.25 and all inducers were added (100 ng/ml aTc, 1 mM l-rhamnose) to each strain at time *t* = 0 h. Antibiotics were added to liquid and solid media as required to maintain genetic constructs, i.e., 25 μg ml^−1^ (liquid) and 50 μg ml^−1^ (solid) nourseothricin, 20 μg ml^−1^ spectinomycin and 25 μg ml^−1^ kanamycin.

### Continuous culture, online measurements and calculations

The continuous culture was performed in a Lambda Photobioreactor (PBR) (Appendix A, Figure A1) in BG11 medium, supplemented with the required antibiotics, at culture volume *V*_ℓ_ = 1 l, aeration with 1 l min^−1^ of CO_2_-enriched (0.5%) air, agitation by the Lambda fish-tail mixing system at 5 Hz, temperature control at 30°C, and pH 8, with 0.5 M NaOH and 0.5 M H_2_SO_4_ as pH correction solutions. After equilibration to these conditions the reactor was inoculated to a start OD_750_ ≈0.5, from 100 ml pre-culture. White light from the Lambda LUMO module was calibrated to μmol m^−2^ m^−1^ (Figure A2E and F) and intensity adjusted to achieve ≈90 μmol m^−2^ m^−1^ per OD_750_ over the experiment (Figure A2F). For evaporation control and continuous culture mode, the total weight of the reactor setup was kept constant using the built-in Lambda reactor mass control module and automatic addition of fresh culture medium through the feed pump. Continuous culture was performed by setting the waste pump to a fixed speed. The PBR was equipped with additional monitoring of optical density by a DASGIP OD4 module, calibrated to offline OD_750_ (Figure A2A and B), and monitoring of offgas O_2_ and CO_2_ concentrations and the weights of feed and pH control bottles by Arduino-based custom-built data loggers (Figure A1). Culture evaporation and dilution rates and biomass growth rates were calculated from the slopes of measured data (Figure A3) using piecewise linear segmentation with our R package dpseg (https://cran.r-project.org/package=dpseg, version 0.1.2 at https://gitlab.com/raim/dpseg/). Cell volume growth rate was calculated as the rate of change of the peaks of the CASY cell volume distributions.

### Biomass measurements: OD, spectra and cell dry weight

The optical density (OD_750_) and absorbance spectra were measured on a Specord200 Plus (Jena Bioscience) dual path spectrometer, using BG11 as blank and reference. Samples were appropriately diluted with BG11 before measuring. All topA^OX^ time series samples were diluted 1:4 before recording OD_750_. For absorbance spectra the OD_750_ was adjusted to 0.5 before measurement. The spectra were all divided by the absorbance at 750 nm.

To determine the cell dry weight (CDW) 5 ml cell culture was filtered through a pre-dried and pre-weighed cellulose acetate membrane (pore size 0.45 μm) using a filtering flask. After that the membrane was dried at 50°C for 24 h and weighed after cooling. 5 ml of filtered and dried growth medium served as a blank. For normalization of glycogen measurements by biomass and for estimation of the biomass density of cells (g_DCW_/ml_cell_), Figure 5A) the OD_λ_ signal was calibrated to CDW (Figure A2C and D).

### Cell count and size distributions

**Figure 2.**
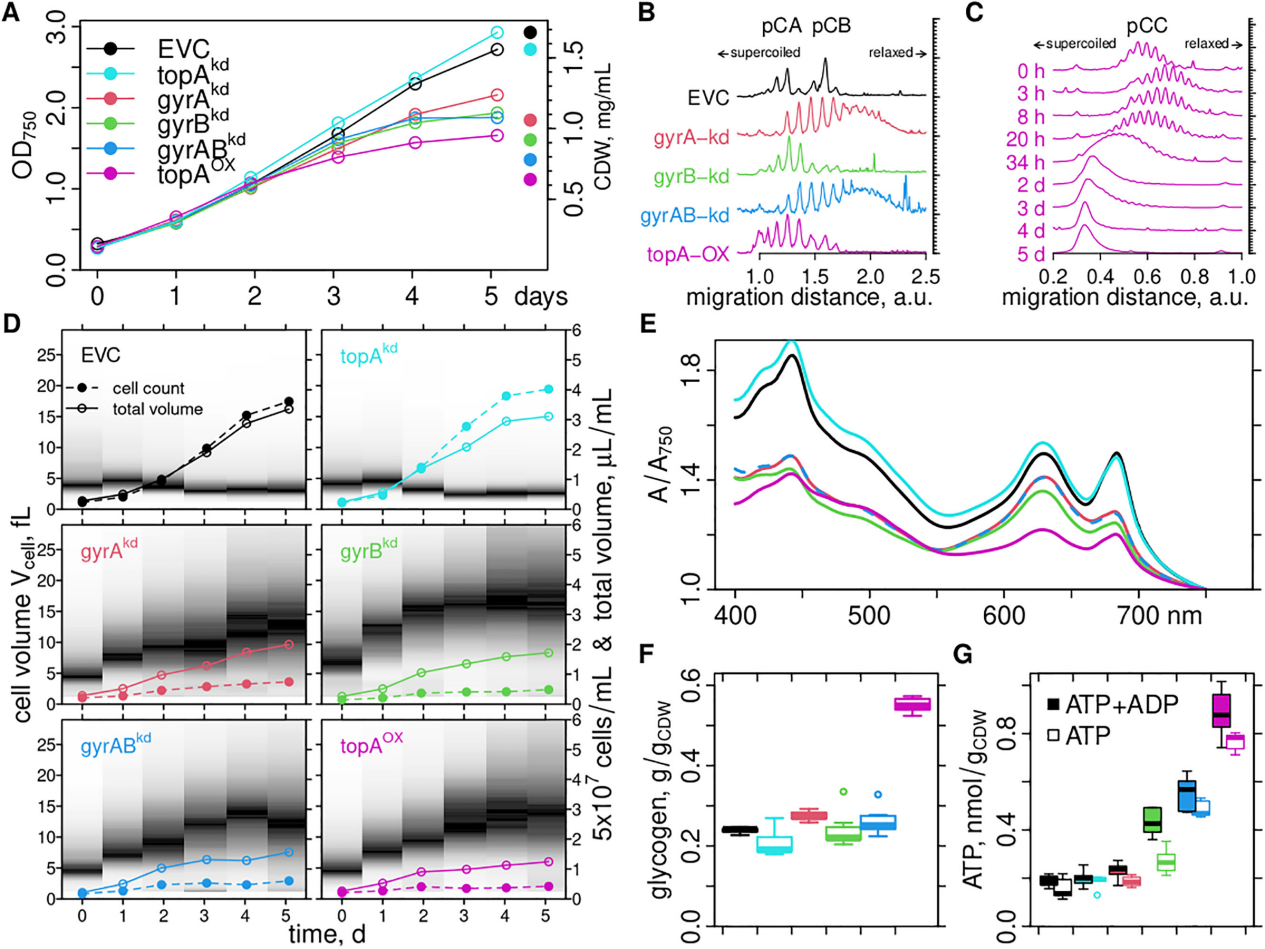
Batch culture endpoint measurements. Overexpression and knockdown strains of this study where grown for 5 days in BG11 medium supplemented with all required antibiotics, and all inducers for the plasmid constructs in each experiment (100 ng/ml aTc, 1 mM l-rhamnose). (**A**) The optical density at 750 nm (OD_750_) was measured daily and cell dry weight (CDW) determined directly after the last measurement on day 5. (**B**, **C**) Electropherograms of chloroquine-supplemented agarose gels (1.2% agarose, 20 μg ml^−1^ chloroquine) of plasmids extracted at harvest time (B) of the cultures in (A), or as a time series (growth curve, Supplementary Figure S2D) of the topA^OX^ strain (C). The migration direction of more supercoiled and more relaxed topoisomers is indicated. See Supplementary Figure S2 for the original gel images. (**D**) Cell counts and size distributions were measured daily in the CASY cell counter and plotted as a gray-scale gradient (black: more cells at this volume). (**E**) Absorption spectra after the harvest on day 5. See Supplementary Figure S1B for spectra at inoculation time. All spectra were divided by the absorption at 750 nm. (**F**) Glycogen content at harvest time was determined by a colorimetric assay after harvest, and boxplots of 18 technical replicates (three samples, each measured 3× in two assays) are shown. (**G**) ATP and ATP+ADP contents at harvest time were determined by a luciferase-based assay, and boxplots of six technical replicates (three samples and two measurements) are shown.

To determine cell counts and size distributions, 10 μl cyanobacteria culture, pre-diluted for OD_750_ measurement, were dispensed in 10 ml CASYton and measured with a Schaerfe CASY Cell Counter (Modell TTC) using a diameter 45 μm capillary. Cell size was recorded in the diameter range 0–10 μm. Each sample was measured with 400 μl in triplicate runs. Analysis of the raw data was performed in R. Counted events in the CASY are a mix of live cells, dead cells, cell debris and background signals. Only counts with diameter *d* > 1.5 μm and *d* < 5 μm were considered for the time series experiment (Figure 5) while a lower cutoff *d* > 1.25 μm was used for the endpoint measurements (Figure 2B) to avoid cutting the distribution of the slightly smaller topA^kd^ cells. Since *Synechocystis* cells are spherical, the cell volumes were calculated from the reported cell diameters *d* as $V_{\rm cell}=(\frac{d}{2})^3 \pi \frac{4}{3}$.

### Glycogen measurement

0.5 ml of cell culture was harvested into reaction vessels that had been pre-cooled on ice, samples were centrifuged at maximum speed (5 min, 4°C). The pellets were flash-frozen in liquid nitrogen and stored at –80°C. The pellets were resuspended in 400 μl KOH (30% w/v) and incubated (2 h, 95°C). For precipitation, 1200 µl ice cold ethanol was added and the mix incubated (over night, –20°C). After centrifugation (10 min, 4°C, 10 000 g), the pellet was washed once with 70% ethanol and again with pure ethanol. The pellets were dried in a Concentrator Plus (Eppendorf) speed-vac (20 min, 60°C). To degrade glycogen to glucose units, pellets were resuspended in 1 ml 100 mM sodium acetate (pH 4.5) supplemented with amyloglucosidase powder (Sigma-Aldrich, 10115) at a final concentration of 35 U/ml and incubated (2 h, 60°C). The sucrose/d-glucose assay kit from Megazyme (K-SUCGL) was applied according to the manufacturer’s specifications to measure the total glucose content, but omitting the fructosidase reaction step and scaling down the total reaction volume to 850 μl. Absorbance at 510 nm was measured using a BMG Clariostar photospectrometer.

### ATP and ADP measurement

2 ml tubes were preloaded with 250 μl of buffer BI (3 M HClO_2_, 77 mM EDTA). 1 ml culture sample was added, vortexed and incubated (lysis, 15 min on ice). 600 μl of BII (1 M KOH, 0.5 M KCl, 0.5 M Tris) were added (neutralization). Samples vortexed and incubated (10 min, on ice), centrifuged (10 min, 0°C, 12 000 g), flash-frozen in liquid nitrogen and stored at –80°C. Extracts were thawed on ice and centrifuged (10 min, 0°C, 12 000 g). 200 μl samples were added either to 320 μl of BIII/PEP (100 mM HEPES, 50 mM MgSO_4_·7H_2_O, adjusted to pH 7.4 with NaOH, and 1.6 mM phosphoenolpyruvate (Sigma-Aldrich, 860077)) for ATP quantification or BIII/PEP + PK (BIII/PEP with 2 U/μl pyruvate kinase, (Sigma-Aldrich, P1506)) for ATP + ADP quantification, incubated (30 min, 37°C), and heat-inactivated (10 min, 90°C). ATP concentrations were determined using the Invitrogen ATP determination kit was used (ThermoFisher: A22066). 10 μl of each PEP or PEP + PK-treated sample was loaded in a white 96-well plate with solid bottom and kept on ice until the reaction was started. The luciferase master mix was scaled down in volume, and 90 μl of master mix was added to each well. Luminescence was recorded using a BMG Clariostar. ATP concentrations were calculated from a standard curve on the same plate.

### Microscopy

500 μl cell culture was sampled four days after induction and mixed with glutaraldehyde to 0.25%. After incubating for 15 min at room temperature (RT) samples were flash-frozen in liquid nitrogen and stored at –80°C. Cells were thawed on ice for 2 h and additionally 30 min at RT. Then washed twice with 1 ml 1× PBS (phosphate buffered saline) and stained with HOECHST 33342 (1 μg ml^−1^, ThermoFisher: 62249) and propidium iodide (30 μm, ThermoFisher: L13152). After 15 min incubation cells were washed with 1 ml 1× PBS. Coverslips (18 × 18  mM, IDL: 19 00 02460) were covered with poly-l-lysine solution for 5 min. Poly-l-lysine solution was removed with a pipette. Coverslips were placed in six-well plates and covered with 1 ml 1× PBS, 10 μl cell suspension was added, and the well plates centrifuged at 1500 g for 15 min. Coverslips were placed on slides and images were captured with the Olympus FluoView FV3000 confocal microscope. HOECHST fluorescence was excited with a 405 nm laser and emission was captured from 430 to 470 nm. Chlorophyll was excited with a 640 nm laser and emission was captured from 650 to 750 nm. Images were analyzed using Fiji (ImageJ, version: 2.1.0/1.53f51). To automatically detect cells and measure cell dimensions the plugin ObjectJ (version: 1.04z) and its Coli-Inspector macro were used (68), with minimum and maximum widths of 0.5 and 3.5 μm, applied to the chlorohpyll fluorescence images. Some objects were manually edited: undetected cells were added, 8-shaped cells recognized as two single cells were merged, and artifacts marked as objects were deleted. To determine the ratios of single cells and 8-shaped cells, cells were counted manually. Images for publication were prepared following the QUAREP-LiMi guidelines (69).

### Flow cytometry and analysis

Samples were fixed in 4% para-formaldehyde in 1× PBS, washed three times in 1× PBS, and stained with the SYTO9 green fluorescent nucleic acid stain from the LIVE/DEAD BacLight kit (ThermoFisher, L13152) according to manufacturer’s instructions. The flow cytometric measurements were taken at the FACS Facility at the Heinrich-Heine University (Dipl.-Biol. Klaus L. Meyer) using a BD FACSAria III. Forward scatter (FSC) and side-scatter (SSC) were recorded. Syto9 was measured with a 530/30 nm filter, and chlorophyll fluorescence was measured with 695/40 nm filter. For each sample 10 000 events (cells, debris and background) were recorded. Data was exported in .fcs format, parsed and analyzed using the flowCore R package (70), and plotted using our in-house segmenTools R package.

### Total DNA and plasmid extractions

To isolate total DNA, 1 ml culture was centrifuged at maximum speed (10 min, 4°C), flash-frozen in liquid nitrogen and stored at −80°C. Thawed samples were resuspended in 1 ml 1× TE buffer, and incubated (1 h, 37°C) with 100 μl lysozyme (50 mg/ml stock solution). 10 μl Proteinase K (20 mg/ml) and 100 μl 20% SDS were added and samples incubated ( 20 h, 37°C). DNA was extracted in Phasemaker Tubes (ThermoFisher: A33248) with one volume of phenol/chloroform/isoamyl alcohol, centrifugation at maximal speed (10 min, 4°C). The upper phase was transferred, mixed with 100 ng/μl RNAse A and incubated (15 min, 37°C). After addition of 1 volume of chloroform/isoamyl alcohol, the centrifugation step was repeated. DNA was precipitated from the upper phase with 1 volume 2-propanol (over night, −20°C), and pelleted by centrifugation at maximal speed (10 min, 4°C). The pellet was washed twice with 500 μl ice-cold 70% EtOH and centrifuged at maximal speed (10 min, at 4°C), dried at room temperature, and resuspended in 30 μl MilliQ water.

To isolate the small endogenous plasmids, 20 ml of cell culture were mixed with 20 ml of undenatured 99.5% ethanol, pre-cooled to −80°C, in 50 ml centrifuge tubes and stored at −80°C until processing. Samples were thawed on ice, centrifuged (10 min, 4°C, 4000 g). The QIAprep Spin Miniprep kit was modified to extract plasmids from the pellet. The cell pellet was resuspended in 250 μl Qiagen P1 solution and transferred to 1.5 ml reaction tubes, 50 μl lysozyme solution (50 mg ml^−1^) was added, and the mix incubated (1 h, 37°C). Then 55 μl of 20% SDS and 3 μl of proteinase K (20 mg ml^−1^) were added and the mix incubated (16 h, 37°C). Further extraction proceeded with alkaline lysis (Qiagen P2) as per manufacturer’s instruction but with volumes adjusted. To enrich covalently closed circular DNA, the samples where digested with the T5 exonuclease (NEB: M0363, 30 min, 37°C), and purified with the QIAprep Spin Miniprep kit.

### Chloroquine agarose gel electrophoresis of plasmids

Agarose gels (1.2%) with 20 μg ml^−1^ chloroquine diphosphate (CQ, Sigma: C6628-50G, CAS: 50-63-5 in 0.5× TBE buffer) were performed as detailed at protocols.io (https://dx.doi.org/10.17504/protocols.io.rbcd2iw) and in a bioRxiv preprint (71), Briefly, gels were run at 1.8 V cm^−1^, protected from light and for 18 h–22 h (as indicated, Supplementary Figure S2), stained with SYBR Gold (ThermoFisher: S11494) and imaged on a BioRad Imaging System (ChemiDoc MP). Electropherograms of each lane were extracted in ImageJ and processed in R, with smoothing and peak detection functions from the msProcess R package (version 1.0.7) (https://cran.r-project.org/web/packages/msProcess/). A baseline was determined in two steps using the msSmoothLoess function. The first step used the full signal and served to determine the coarse positions of peaks. The final baseline was then calculated from the signal after removal of peak values and subtracted from the total signal and subtracted from all electropherograms.

### RNA extraction and processing

1 ml culture was added to 250 μl pure ethanol supplemented with 5% phenol, flash-frozen in liquid nitrogen and stored at −80°C. RNA was extracted after (72) with some modifications. Frozen samples were centrifuged (10 min, 4°C, maximum speed), and the pellet resuspended in 1 ml PGTX (per 1 l: 39.6  g phenol, 6.9 ml glycerol, 0.1 g 8-hydroxyquinoline, 0.58 g EDTA , 0.8 g sodium acetate, 9.5 g guanidine thiocyanate, 4.6 g guanidine hydrochloride and 2 ml Triton X-100) and incubated (5 min, 95°C). After cooling on ice for 2 min, 700 μl chloroform:isoamyl alcohol (24:1) was added and the mixture incubated ( 10 min, room temperature) while shaking gently. The mixture was centrifuged (10 min, 4°C, maximal speed). The upper phase was transferred to a fresh tube and 1 volume chloroform:isoamyl alcohol was added. After repeating the centrifugation step, the upper phase was transferred and precipitated with 3 volumes of 99.5% ethanol and 1/2 volume 7.5 M ammonium acetate and (time series only) 1 μl RNA-grade glycogen at −20°C over night. The RNA was pelleted by centrifugation (30 min, 4°C, maximum speed), washed twice with 70% ethanol and resuspended in 30 μl RNase-free water. Volumes were adjusted to contain 2 μg of nucleic acid (Nanodrop), and DNA was removed by DNaseI (ThermoFisher: EN0525) according to the manufacturer’s specifications but at 2× reaction buffer concentration. RNA was extracted as above but using 1/10 volume of 3 M sodium acetate (pH 5.3) instead of ammonium acetate.

### Quantitative RT-PCR

100 ng DNaseI-digested RNA samples were reverse-transcribed to cDNA using the RevertAid RT (ThermoFisher: K1621) according to the manufacturer’s specifications in a reaction volume of 20 μl, and RT-qPCR performed with the DyNAmo ColorFlash SYBR Green qPCR-Kit (ThermoFisher: F416L). Briefly, 60 μl RNase-free water was added to the cDNA reaction mix. 2 μl (2.5 ng) were transferred into qPCR 96-well microplates and 8 μl Master Mix added. Primer efficiencies (Supplementary Table S3) were assessed from calibration curves. Primers were added at a final concentration of 0.5 mM. The thermal cycling conditions were: 7 min at 95°C, followed by 40 cycles of 5 s at 95°C and 30 s at 60°C. Melting curves were recorded for each sample to ensure sample purity. RT-negative controls and no-template-controls (distilled water) were included for each run. Each sample was loaded in technical triplicates. Gene expression changes at indicated time points were then quantified by the ΔΔ*Ct* method (73), using *rpoA* as a reference gene (74), and a time point before induction of genetic construct (time series) or the empty vector control (EVC) strain (batch culture endpoint experiments) as the reference expression state. ΔΔ*Ct* is then the log_2_ fold-change with respect to this reference state. MIQE guidelines were followed where applicable.

### RNAseq: total RNA analysis, library generation and sequencing

DNaseI-digested RNA samples (25 μl) were sent for sequencing analysis. RNA quality was evaluated spectrometrically by Trinean Xpose (Gentbrugge, Belgium) and by size separation by capillary gel electrophoresis on an Agilent 2100 Bioanalyzer with the RNA Nano 6000 kit (Agilent Technologies, Böblingen, Germany). For total RNA analysis, electropherograms were parsed from exported XML files using the R package bioanalyzeR (v 0.9.1, obtained from https://github.com/jwfoley/bioanalyzeR) (75), and each lane was divided by the total RNA content as reported by the Agilent 2100 Bioanalyzer software. The Illumina Ribo-Zero Plus rRNA Depletion Kit was then used to remove the ribosomal RNA, and removal confirmed by capillary gel electrophoresis as above. Preparation of cDNA libraries was performed according to the manufacturer’s instructions for the TruSeq stranded mRNA kit (Illumina, San Diego, CA, United States). Subsequently, each cDNA library was sequenced on an Illumina NextSeq 500 system (2 × 75 nt, PE high output v2.5).

### RNAseq: read mapping

The resulting sequence reads were quality trimmed with Trimmomatic v0.33 (76) using standard setting. The quality trimmed reads were subsequently mapped to coding genes of the Synechocystis sp. PCC 6803 reference genome, its seven endogenous plasmids and our pSNDY construct (Supplementary Table S4) using Bowtie 2 (77). For the endpoint measurements from batch cultures the log_2_ fold changes with respect to the control (EVC) were calculated with the DESeq2 algorithm (78) *via* the ReadXplorer software version 2.0 (79), based on three replicate measurements for each strain (‘M-value’), and these values are denoted log_2_(<strain>/EVC) in figures, where <strain> is the tested strain and EVC is the control strain. For the analysis of the expression gradient within transcription units, the difference of these values between the first and the last transcribed gene of each TU was taken. This difference equals the log_2_ ratio of the fold changes. For the time series read count data were normalized by library sizes to the transcripts per kilobase million (TPM) unit. Missing values at individual time points were interpreted as 0 TPM. For plots, the log_2_ fold change of each time point to the mean of the two pre-induction time points was calculated, denoted as $\log _2\left(x_i/\overline{x_{1,2}}\right)$ in figures.

### Clustering analyses

For clustering the time series into co-expressed groups, a previously established pipeline was used (80,81). The input time series were RNAseq samples 2 to 16 (from –0.5 to 72 h around the time of induction at 0 h), i.e., without the first pre-induction time-point and ignoring the two long-term response samples (Supplementary Figure S12C). Briefly, the time-series of TPM values was arcsinh-transformed, the Discrete Fourier Transform (DFT) *X*_*k*_ was calculated, each DFT component *k* > 0 normalized ($X^{\prime }_k$) to the mean of amplitudes at all other components *k* > 1. The real and imaginary parts of selected components $X^{\prime }_{k=1,\dots ,6}$ were then clustered with the flowClust algorithm (82) over cluster numbers *K* = 2, …, 10. The clustering with the maximal Bayesian Information Criterion, as reported by flowClust (Supplementary Figure S12A), was selected for further analyses. Data transformation and clustering were performed by the processTimeseries and the clusterTimeseries2 functions of segmenTier and segmenTools R packages (81), respectively. The resulting clusters were sorted and colored based on the comparison with diurnal co-expression cohorts (Figure 6 and Supplementary Figure S17) for informative plots of the subsequent analyses. To map this clustering from genes to transcription units (TU) (83), the mean expression of all coding genes in each TU was calculated. The resulting TU time-series were then clustered by k-means, using the cluster centers from the gene-based clustering as input (Supplementary Figure S18). To estimate the immediate transcriptional response to *topA* overexpression the log2 ratio of the means of the two post-induction time points (5 min, 20 min, or as indicated) to the means the two pre-induction time points (−1 day, −35 min) were calculated ($\log _2\left(\overline{x_{3,4}}/\overline{x_{1,2}}\right)$). Transcripts with negative values (<−θ) were labeled as ‘down’, with positive values (>θ) as ‘up’, and all others as ‘nc’ (for ‘no change’). A low threshold θ = 0.01 was used for the gene-level analysis (Figure 6D, E), since here a comprehensive picture of directionality was desired, and a stricter θ = 0.15 for TU-level analysis (Supplementary Figure S18C) since the extremes were of interest for promoter structural analysis. Diurnal expression data (84) were obtained from GEO (GSE79714) and genes summarized as the mean over all associated probes. These expression values were clustered (Supplementary Figure S17) as described for the RNAseq data, but using the flowclusterTimeseries function.

### Cluster enrichment profiles

Categorical enrichments, e.g., coding gene co-expression cohorts vs. gene annotations, were analyzed by cumulative hypergeometric distribution tests (R’s phyper) using segmenTools’s clusterCluster function and the clusterAnnotation wrapper for GO and and protein complex analysis, which compares overlaps of each pair of two distinct classifications into multiple classes, and stores overlap counts and *P*-values (enrichment tables). To analyze log_2_ fold-changes by clusters two-sided t-test were performed (R base function *t*-test, incl. Welch approximation for different sample sizes), comparing the distribution of values of the cluster with all other values (function clusterProfile).

For intuitively informative plots the enrichment table rows were sorted along the other dimension (table columns) such that all categories enriched above a certain threshold *p*_sort_ in the first column cluster are moved to the top, and, within, sorted by increasing *p*-values. Next, the same sorting was applied to all remaining row clusters for the second column cluster, and so on until the last column cluster. Remaining row clusters are either plotted unsorted below a red line or removed. This is especially useful to visualize enrichment of functional categories along the temporal program of co-expression cohorts, e.g., Figure 6B. This sorting is implemented in segmenTools’ function sortOverlaps.

Sorted enrichment tables were visualized as colored table plots (Enrichment Profiles) (e.g. Figure 6B, C), using segmenTools’ function plotOverlaps. For the categorical overlap tests, the total counts of overlapping pairs are plotted as text, and for t-test profiles the rounded *t* statistic. The text color is black or white based on a p-value cutoff *p*_txt_ (as indicated). The field background color intensities scale with log_2_(*p*) of the reported p-values, where the full color corresponds to a minimal p-value *p*_min_ cutoff (as indicated) and white reflects *p* = 1. For categorical enrichment tests the full color is black. For numerical tests, the sign of the *t* statistic is used to determine a color to indicate the direction of change: red for negative values (*t* < 0, downregulated) and blue for positive values (*t* > 0, upregulated).

### Promoter nucleotide frequency profiles

Only transcription units from the main chromosome were considered for promoter structure analysis. The genome sequence was converted into a vector of 0 and 1, where 1 indicates occurrence of the motif under consideration. Motif occurrence vectors upstream and downstream of transcription start sites were extracted from the genome vector and aligned into a matrix (columns: positions around the alignment anchor, rows: all genomic sites under consideration). The occurrence of a motif in all sequences of a cluster were counted at each position in 66 or 5 bp windows surrounding the position. Cumulative hypergeometric distribution tests (R’s phyper) were performed to analyze statistical enrichment or deprivation within the window of all TU in a cluster vs. the same window in all TU. The mean position-wise motif occurrence (frequency, in %) was plotted on the y-axis and the size of the plotted data point was scaled by the enrichment and deprivation p-values to emphasize regions of significant difference. The maximal size was determined by the minimum p-value in each test series, as indicated in the Figure legends. The point style (closed or open circles) indicates the directionality of the test (enriched or deprived). These significance points are shown at every third or tenth position to avoid overlaps.

### Other data sources

Genome sequences and annotation were downloaded from NCBI (Supplementary Table S4). The gene ‘categories’ annotation was downloaded on 2017-09-23 from CyanoBase, file category.txt (85). Gene Ontology annotation was downloaded from the UniProt database (2021-03-20, organism:1111708) (86). Datasets from other publications were all obtained from the supplemental materials of the indicated publications.

## RESULTS

### Cell division block and redirection of cellular resources

#### Manipulation of gyrase and Topoisomerase I expression

Based on the current models of the role of DNA supercoiling homeostasis in bacteria (Figure 1A), we hypothesized that artificial genome relaxation should inhibit growth and redirect metabolic flux. To test this idea, we constructed three strains (Supplementary Table S1) to inducibly repress (knockdown, kd) gyrase subunits with the dCas9-mediated CRISPR-interference system (64), and one strain to overexpress TopoI: strains gyrA^kd^ (target: *slr0417*), gyrB^kd^ (*sll2005*) and gyrAB^kd^ (both subunits), all inducible by anhydrotetracycline (aTc); and strain topA^OX^ with *slr2058* with a rhamnose-inducible promoter on the pSNDY plasmid (63). As controls, we included a TopoI knockdown strain (topA^kd^), and an empty vector control (EVC) strain, bearing all plasmids but without the sgRNA or the *topA* gene. All six strains were induced with aTc and rhamnose and cultured in continuous light for 5 days (Figure 2A), then harvested for quantification of plasmid supercoiling, cell dry weight, ATP + ADP, and glycogen. Reverse transcription quantitative PCR (RT-qPCR) verified the functionality of our inducible genetic constructs, but two reference genes gave disparate results (Supplementary Figure S1A). This points to global changes of the transcriptome and precludes quantification in terms of fold changes by RT-qPCR, which we resolve below by RNAseq analysis.

#### Hypernegative plasmid supercoiling in the topA^OX^ strain

To analyze the specificity of our manipulations, we first investigated the effects on plasmid supercoiling by agarose gel electrophoresis in the presence of an intercalator. Samples taken from the harvest time of the batch growth experiments showed three sets of topoisomer bands (Supplementary Figure S2A), consistent with the presence of three annotated small plasmids of *Synechocystis*, pCA2.4_M, pCB2.4_M and pCC5.2_M. Electropherograms of the two smaller plasmids indicate that only strains gyrA^kd^ and gyrAB^kd^ showed plasmid relaxation (Figure 2B). We could not extract plasmids from the topA^kd^ strain (Supplementary Figure S2A). Unexpectedly, plasmids in the topA^OX^ and gyrB^kd^ strains had a higher level of supercoiling. To investigate this effect, we measured plasmid supercoiling as a time series of the topA^OX^ strain after inoculation in fresh medium with and without the inducer (Supplementary Figure S2B–F). The gel run time was increased to better separate topoisomers of pCC5.2_M. All three plasmids were more relaxed after induction for 3 h (Figure 2C). Already after 8 h the trend had reversed, and at 20–34 h plasmids were more supercoiled than at time 0 h and in the uninduced control time series (Supplementary Figure S2C). Then plasmids became further supercoiled to an extent where topoisomers were not separable anymore. In summary, the effects on plasmids verify the functionality of our constructs on protein activity level, and indicate quick compensatory reactions.

#### Cell volume growth, and increased adenosine and glycogen content

Next, we investigated the phenotypes to test the hypothesis that genome relaxing manipulations could set free cellular energy for potential use in bioproduction. Initially, all cultures showed comparable growth. After three days all strains except topA^kd^ grew slower than the EVC; and topA^OX^ showed the strongest growth defect. The cell dry weight (CDW) at harvest time correlated with the final OD_750_ of the cultures (Figure 2A), but was relatively higher for the EVC and topA^kd^ strains. Cell volume distributions of the EVC and topA^kd^ strains showed a transient small increase ($\approx {10}\%$) on the first day of cultivation and were stable thereafter (Figure 2D). In contrast, cell volumes of the gyr^kd^ and topA^OX^ strains increased over time, from 4 –5 fL to 12–15 fL after four days of cultivation. Total cell numbers increased only slightly. Thus, strains where gyrase subunits were knocked down or TopoI was overexpressed showed inhibition of cell division but not of cell growth. Absorption spectra (Figure 2E, Supplementary Figure S1B) showed an overall decrease of all pigments in topA^OX^. The gyr^kd^ strains showed a stronger decrease at chlorophyll-specific wavelengths than at phycocyanin-specific wavelengths. All knockdown strains showed glycogen levels similar to the EVC, with 25 % of the total CDW (Figure 2F). In contrast, topA^OX^ contained more than twice as much glycogen, 55% of the CDW, and more than four times as much ATP+ADP as the EVC (Figure 2G). gyrB^kd^ and gyrAB^kd^ accumulated about twice as much ATP+ADP as the EVC; topA^kd^ and gyrA^kd^ showed no difference to the EVC control. While the strains show clear differences in their metabolic phenotype, the volume growth phenotype is consistent for all manipulations that should (in principle) decrease supercoiling and not seen in the two controls; a further verification of the functionality of our constructs.

#### Confirmation by microscopy and flow cytometry

**Figure 3.**
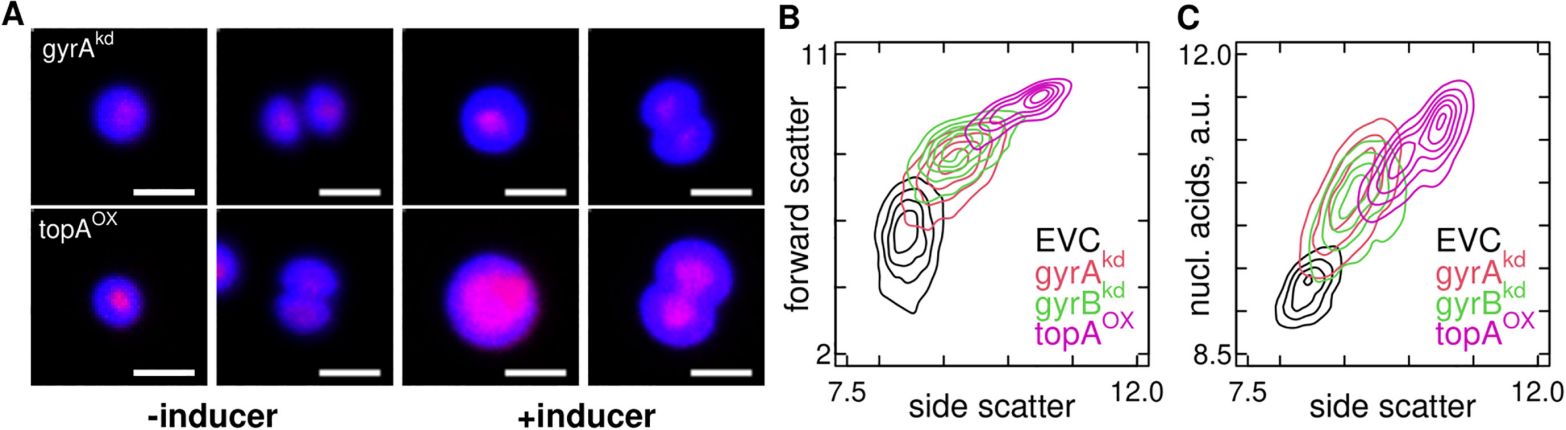
Microscopy and Flow Cytometry Confirm the Volume Growth Phenotype. (**A**) Fluorescence microscopy images of typical round and dividing cells, after 4 days of growth with or without the inducers (Supplementary Figure S3). The bar indicates 2 μm. Chlorophyll-specific fluorescence is shown in blue and DNA-specific (HOECHST 33342) fluorescence in red. Bright-field and single channel images are provided in Supplementary Figure S4. (**B**, **C**) Flow cytometry after 6 days of growth in the presence of the inducers (Supplementary Figure S6). The natural logarithms of forward scatter, side scatter (B) and nucleic acid stain Syto9 (C) were calculated and 2D distributions plotted as contour plots (flow cytometry raw data: Supplementary Figure S7).

The conductivity-based cell sizes provided by the CASY system does not distinguish cell shape. We thus confirmed the volume growth phenotype by fluorescence microscopy and flow cytometry, each with nucleic acid staining. Cell volumes were increased in the gyrA^kd^ and topA^OX^ strains only in the presence of the inducers (Figure 3A, Supplementary Figures S3 and S4). The cell size distributions, measured from microscopy images with the Coli-Inspector (68) (Supplementary Figure S3D), agreed well with the CASY data (Supplementary Figure S5). Manual counting of cells in division (8-shaped) or estimation from the distribution of cell widths and lengths showed an increase from $<{10}\%$ to $\approx {20}\%$ after four days of growth in the presence of the inducers (Supplementary Figure S5D, E). The phenotype was further confirmed by flow cytometry (Figure 3B, Supplementary Figure S7): forward scatter (FSC), which reflects cell size, was increased in all strains, and most in topA^OX^. Total nucleic acid content (RNA+DNA) also increased with cell size (Figure 3C).

#### Reduction of rRNA, global remodeling of mRNA & homeostatic regulation of supercoiling enzymes

**Figure 4.**
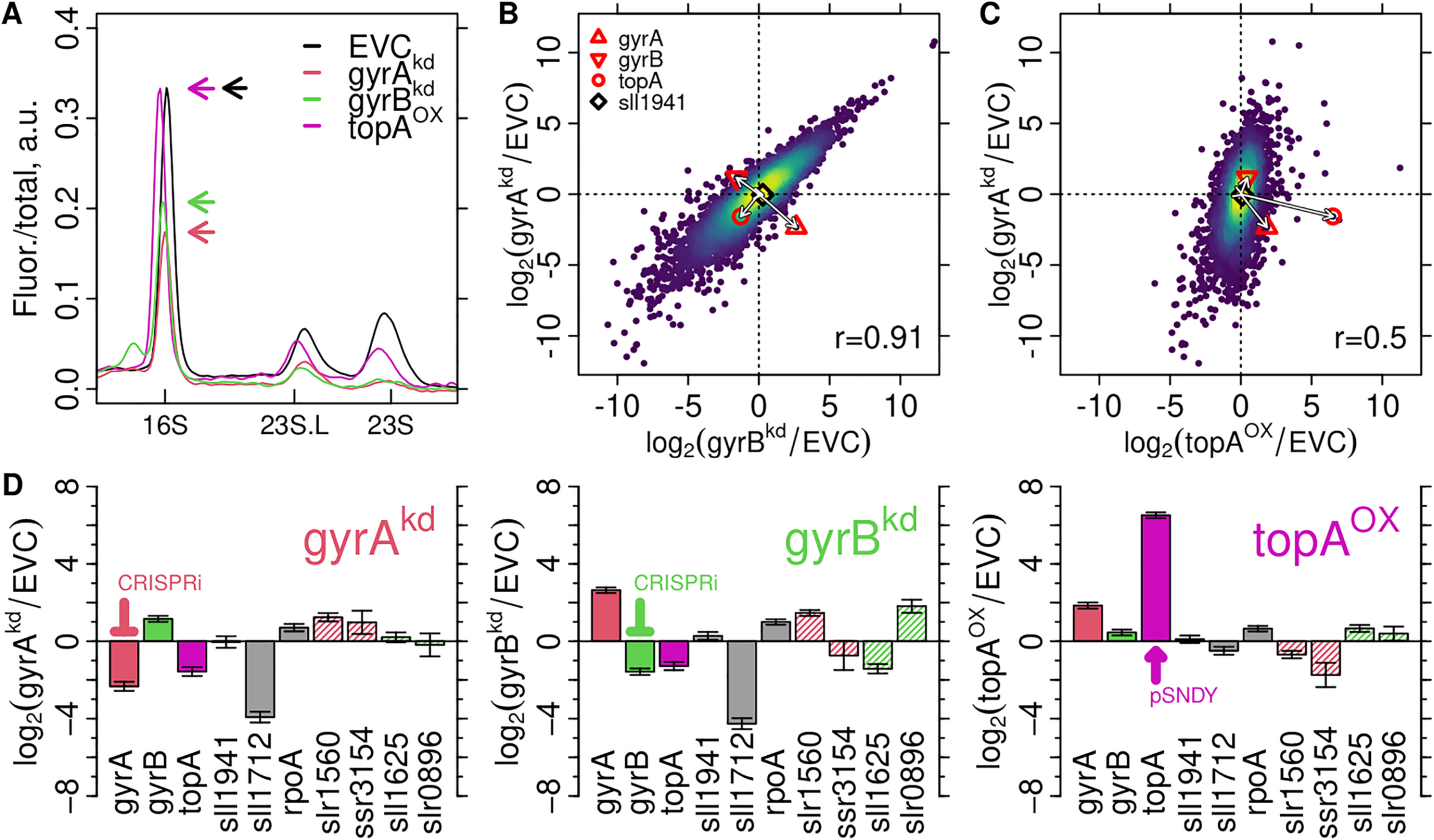
Global transcriptome changes and homeostatic regulation of topoisomerase genes. (**A**) Electropherograms of the capillary gel electrophoresis analysis of extracted RNA used for RNAseq. The fluorescence signal of each lane was normalized by the total RNA content as reported by the Bioanalyzer 2100 software (Supplementary Figure S6C). Lines are the means of three replicates (Supplementary Figures S8 and S9). Locations of the 16S, the 23S and the large fragment of the 23S rRNA (23S.L) are indicated on the x-axis. Arrows indicate the maxima of the 16S rRNA peaks. (**B**, **C**) Expression changes of coding genes in induced strains relative to the control strain (EVC) were derived as the log_2_ ratio of RPKM normalized read counts and then compared between the three different strains by 2D histograms (yellow: highest and purple: lowest local density of genes). The Pearson correlations (r) are indicated in the bottom right corner. (B**)** gyrA^kd^ (y-axis) versus gyrB^kd^ (x-axis) strains. (C) gyrA^kd^ (y-axis) versus topA^OX^ (x-axis). The induction/repression and the homeostatic responses of *gyrA*, *gyrB* and *topA* are highlighted by arrows from the origin to indicate the direction of change. (**D**) Expression changes of the targeted topoisomerase genes, the *gyrA*/*parC* homolog *sll1941*, the HU protein (*sll1712*), the qPCR reference *rpoA*, and the predicted CRISPRi off-targets (indicated by colored stripes). Error bars are standard errors reported by DESeq2.

To investigate the effects on transcription, the same cultures that were used for flow cytometry (Supplementary Figure S6A) were subjected to transcriptome analysis. Total RNA composition and the relative abundances of rRNA and mRNA were analyzed by capillary gel electrophoresis (Supplementary Figures S6B, C, S8). Ribosomal RNA species were strongly reduced in the gyrA^kd^ and gyrB^kd^ strains and less reduced in topA^OX^ (Figure 4A, Supplementary Figure S9). Interestingly, the reduction was stronger for the 23S than for the 16S subunit, even though they are synthesized as one transcript, with 16S upstream and 23S downstream, and processed into subunits co-transcriptionally (87). The same RNA samples were further processed (rRNA species depleted) and sequenced on the Illumina platform, and transcript abundances relative to the EVC control strain (fold change) evaluated with DESeq2 (78). All strains showed overall similar expression changes, but the extent was lower in topA^OX^ (Figure 4B, C). However, this difference could also just reflect normalization effects (88) by the decreased rRNA content in the gyrase knockdowns. In all strains, the targeted manipulation was still observable at harvest time (Figure 4B–D), i.e., *gyrA* transcripts were reduced in gyrA^kd^, *gyrB* transcripts in gyrB^kd^ and *topA* transcripts were increased in topA^OX^. The non-manipulated genes showed the compensatory response expected from homeostatic regulation, i.e., *topA* was repressed in both gyr^kd^ strains, and all non-manipulated gyrase subunits were upregulated in all experiments. Transcription of the DNA binding protein HU (*sll1712*) was strongly downregulated in the gyr^kd^ strains but only weakly in the topA^OX^ strain. In contrast, the *sll1941* gene, annotated either as a second gyrase A subunit or as the topoisomerase IV ParC subunit (89,90), showed no response in either experiment. The upregulation of the qPCR reference gene *rpoA* in all strains explains the disparate results of RT-qPCR (Supplementary Figure S10B–D). Both CRISPRi constructs have potential off-targets (Supplementary Table S2). Indeed, the succinate dehydrogenase gene *sll1625* (91,92) a predicted off-target of the *gyrB*-specific sgRNA was downregulated in the gyrB^kd^ strain (Figure 4D), while other off-targets were not systematically affected.

### Dynamic response and adaptation to topoisomerase I overexpression

**Figure 5.**
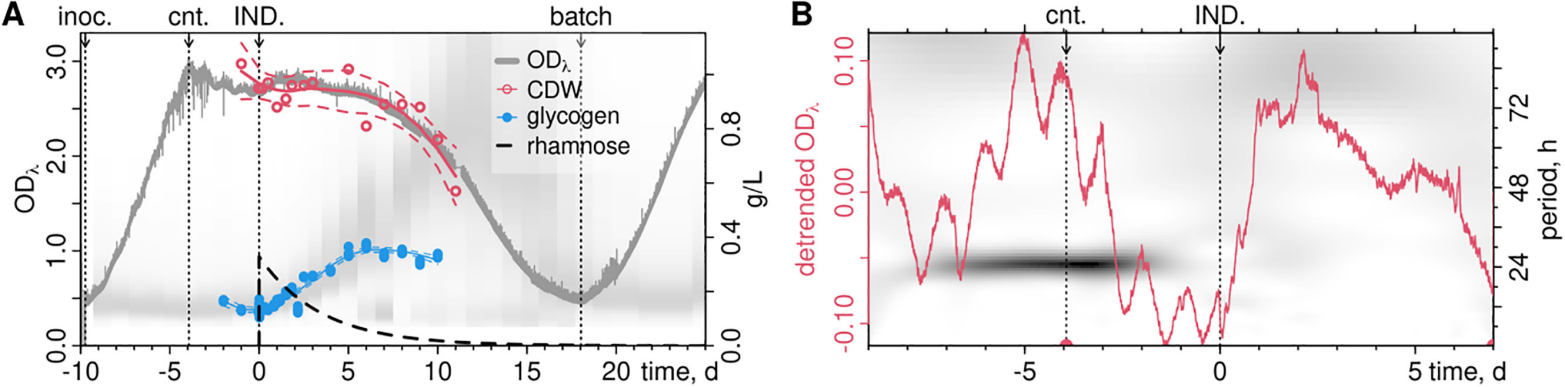
Pulsed induction in continuous culture. (**A**) Photobioreactor growth of the topA^OX^ strain (1 l BG11 medium, 0.5% CO_2_, illumination ≈90 μmol m^−2^ m^−1^ per OD_750_). Optical density was recorded online (OD_λ_) and post-calibrated to offline OD_750_. The arrows indicate inoc.: inoculation; cnt.: onset of continuous culture (rate ϕ = 0.01 h^−1^); IND.: induction of *topA* by pulse-addition of rhamnose to 2 mM (0.33 g l^−1^) at time 0 day; and batch: switch-off of dilution. The dashed black line shows the theoretical wash-out curve of rhamnose ( g l^−1^). Cell dry weight (CDW, g l^−1^, red) and glycogen content ( g l^−1^, blue) were measured at the indicated times (points), and LOESS regressions are shown (solid lines) with 95% confidence intervals (dashed lines). The CASY-based cell volume distributions (Supplementary Figure S11A) are shown as a background in gray-scale for reference. (**B**) The detrended OD_λ_ signal (red line, Supplementary Figure S11D) shows a ≈24 h trend throughout batch phase and continuous culture before induction (IND.) A wavelet analysis of the dominant periods in the signal is shown as gray-scale background (right axis).

Compensatory regulation of the non-manipulated topoisomerase genes in each strain was observed even five days after induction. Ribosomal RNA synthesis was strongly impaired. These observations are compatible with the established models of the role of supercoiling in bacterial transcription (Figure 1). The resulting phenotypes may therefore reflect such compensatory regulation. To investigate the direct effects of our manipulations, we selected the strain with the most pronounced phenotype, topA^OX^, and studied the transient effects after induction in continuous culture. The culture was grown in continuous light at OD_750_ ≈ 2.7 and with a dilution rate ϕ ≈ 0.24 d^−1^ (Figure 5A, Supplementary Figure S11). After pulse-addition of the inducer rhamnose, the *topA* transcript abundance increased to ≈ 45x over its pre-induction level (Supplementary Figure S10F, G). Cell division was inhibited and cell volumes increased with similar kinetics as in the batch culture experiments. Glycogen content increased to ≈40 % of the CDW. After inducer wash-out, cells recovered to their pre-induction state. Appendix A provides a detailed record of these culture dynamics. The online OD signal (OD_λ_) showed a subtle ≈24 h component which vanished after *topA* induction (Supplementary Figure S11D). Sustained circadian rhythms in constant light have been reported before (93). However, we sampled daily for OD_750_ and absorption spectra, and can not exclude that we inadvertently entrained the culture. Sampling in high temporal resolution may similarly have affected the disappearance of the signal after induction.

#### Dynamic transcriptome response in continuous culture

**Figure 6.**
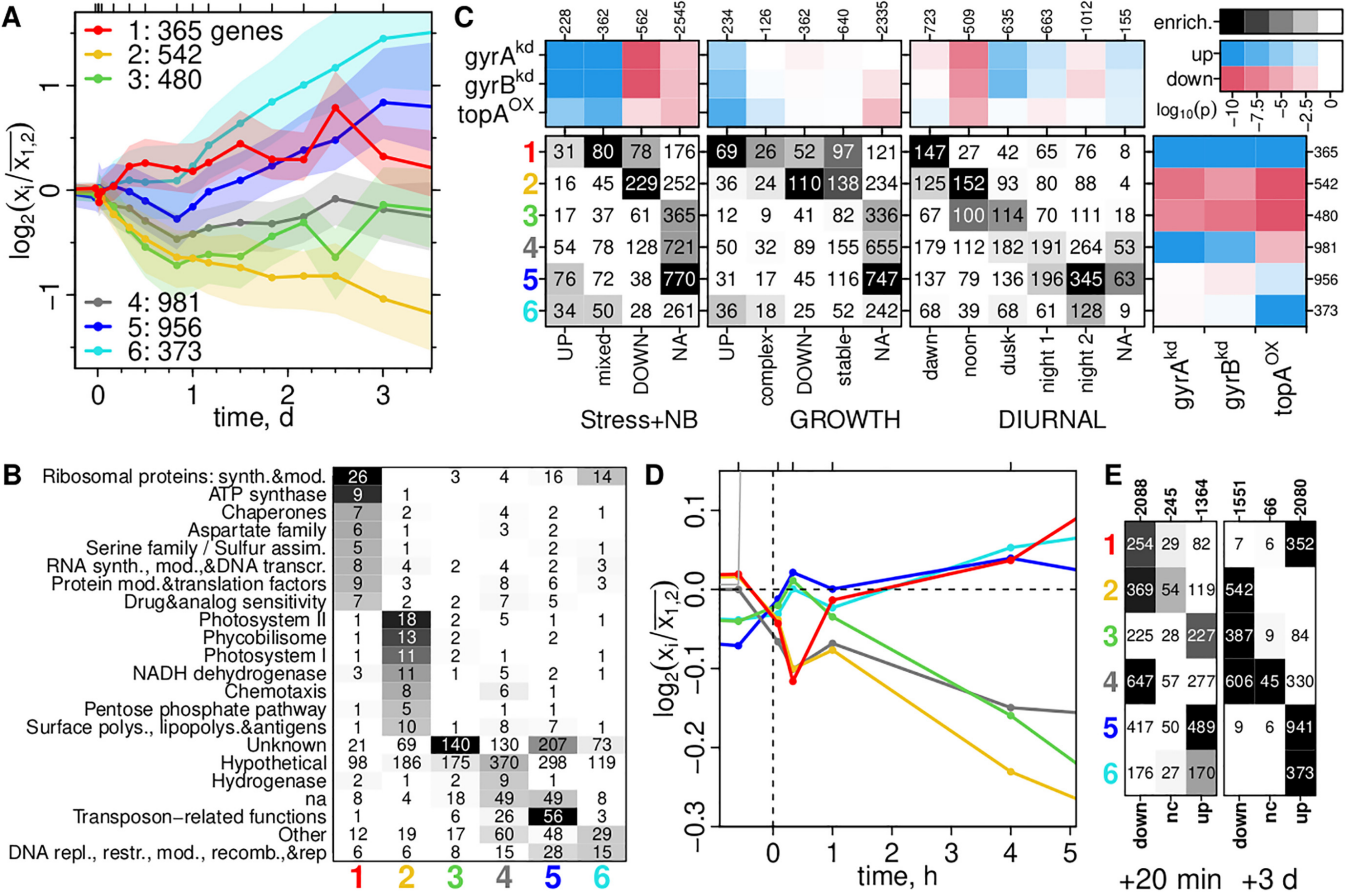
Cluster analysis of the transcriptome time series data. (**A**) Cluster medians of transcript abundances (solid lines), relative to the mean of two pre-induction samples. The transparent ranges indicate the 25%/75% quantiles; points and ticks on upper axis indicate the RNAseq sampling times. Cluster labels (1–6) and sizes (number of genes) are indicated in the legend. (**B**) Sorted enrichment profile of the six clusters with the CyanoBase ‘categories’ gene annotation. The numbers are the gene counts in each overlap, and gray scale indicates the statistical significance (enrichment) of these counts (black field: *p*_min_ ≤ 10^−10^; white text: *p*_txt_ ≤ 10^−5^). Only overlaps with *p*_sort_ ≤ 0.01 are shown (full contingency table in Supplementary Figure S14A). (**C**) Enrichment profiles (gray scale as in (B)) with other published gene classifications (see text) and t-value profiles (red-blue scale, Supplementary Figure S13) of clusters in the end-point transcriptome experiments. Blue indicates upregulation (*t* > 0) and red downregulation (*t* < 0). (**D**) Cluster medians as in (A) but zoomed in on the first 5 h after induction. (**E**) Cluster enrichment profile (gray scale as in (B)) with genes upregulated (up), downregulated (down) or without change (nc), 5–20 min (left) or 2.5–3 days (right) after induction.

Samples for RNAseq analysis were taken at three different time scales, i.e., in highest resolution around induction (–35, 5, 20, 60 min), then over 3 days (4–8 h time steps), and three further samples until 26 days, covering the phases of volume increase and recovery. Coding gene transcript read counts were calculated, the resulting time series clustered (Figure 6A, Supplementary Figure S12) and clusters sorted based on the following analysis. The clusters were scanned for statistical enrichments with functional category annotations (Figure 6B, Supplementary Figures S14 and S15) and with clusterings from published experiments. Specifically, we tested for enrichments of (i) genes that responded coherently to stress conditions in the presence or absence of the gyrase inhibitor novobiocin (13), (ii) genes that were either upregulated or downregulated with increasing growth rate (94), and (iii) two diurnal (light/dark) time series (53,84) that were clustered with the same method (Supplementary Figure S17).

Over the first three days post-induction (Figure 6A), cluster 1 (red) transcripts were upregulated in waves with a ≈24 h pattern and afterwards returned to pre-induction levels. This cluster is enriched with genes encoding for ribosomal proteins and biosynthetic enzymes (Figure 6B), with genes that positively correlated with growth rate, and genes that peaked at dawn (Figure 6C, Supplementary Figure S14B). Cluster 2 (yellow) transcripts were downregulated in our experiment and comprise most photosynthesis genes, and overlap with gene groups that were downregulated in stress conditions, negatively correlated with growth rate, and peaked at noon. Cluster 3 (green) transcript abundances initially decreased and showed a weak ≈24 h pattern, opposite to the transcripts of cluster 1. It is enriched with genes peaking at noon or dusk. The transcript abundances of clusters 5 and 6 (blue and cyan) increased from 1 day post-induction, were enriched with genes that peak at night, with DNA replication and repair machinery, and with transposons. These clusters also contain most plasmid-encoded transcripts (Supplementary Figure S16), and were not upregulated in the endpoint measurement of the gyr^kd^ strains (Figure 6C). And finally, the largest cluster 4 (gray) comprises the genes with the weakest response to induction of *topA* overexpression. In summary, *topA* overexpression differentially affected gene cohorts that overlapped with genes whose transcript levels change over the diurnal cycle (84) and vary with growth rates (94). Diurnal cohorts that are expressed at night or at dawn were upregulated, while cohorts expressed at noon and dusk were downregulated.

### Alignment of −10 and TSS with the structural code

**Figure 7.**
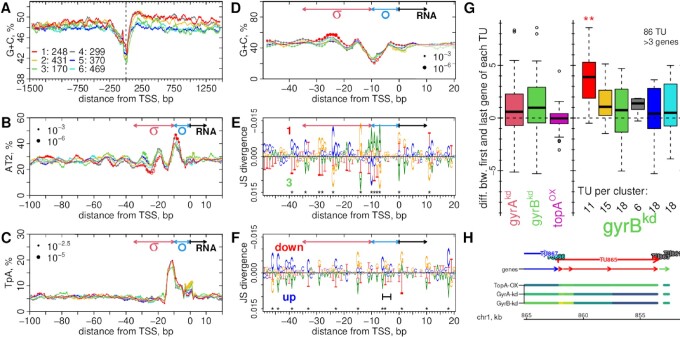
Promoter and Transcription Unit (TU) Structure. (**A**–**D**) Cluster nucleotide frequencies around transcription start sites (TSS) (Supplementary Figure S18); only TU on the main genome were considered and the legend in (A) provides the number of TU in each cluster. The G+C content in (A) was calculated in 66 bp windows at each position, all others in 5 bp windows. Point sizes (B–D) scale with −log_2_(*p*) from local motif enrichment (filled points) and deprivation (open circles) tests, and the minimal *p*-values in each plot are indicated in the legends. The sigma factor binding region (σ, −35 to −10), the location of the open bubble ($\mathbf {\circ }$, −10 to 0) and the transcript (RNA, from 0) are indicated. See Supplementary Figures S19–S23 for the full analysis. (**E**, **F**) The Jensen–Shannon (JS) divergence (Supplementary Figures S24–S25) between the position weight matrices of time series clusters 1 and 3 (E) and of immediate response clusters ‘up’ and ‘down’ (F); * indicates *p* < 0.05 (Supplementary Figure S24) (95). The short horizontal bar in (F) indicates the GC discriminator region −6 to −3. (**G**) Graded response along TU with ≥4 genes in the batch culture experiments in Figure 4. The *y*-axis shows the difference of the log_2_ fold changes between the first and last transcribed gene of each TU. The left panel shows all strains and the right panel the gyrB^kd^ strain and TUs by their cluster association. See Supplementary Figure S27 for all strains and all TU with ≥2 genes. (**H**) An example TU from cluster 1 (red) with a transcript abundance gradient in the gyr^kd^ strains but not in topA^OX^ strain. The genes on TU865 are, from 5’ to 3’, *rps20*, *tatD*, *rpoB* and *rpoC2*. The color scheme (viridis) in the strain tracks reflects the log_2_ fold-changes (Figure 4), where yellow indicates higher and blue lower expression than the control strain (EVC). The colors of genes and TU reflect their time series cluster association.

Our intervention thus revealed gene groups that were also co-regulated in previous experiments. To analyze the underlying promoter structures we mapped the clustering onto transcription units (TU) (83) (Supplementary Figure S18), and calculated the nucleotide content around their transcription start sites (TSS). To avoid bias we only considered TU from the main genome for these analyses. As expected from many other bacterial species (10,12,14,16), the differential response to manipulation of supercoiling correlates with the G+C content of the coding region (Figure 7A). This is especially pronounced in the TU that were most upregulated or downregulated 20 min after induction, and in the typical direction, i.e., upregulated TU are A+T-rich and downregulated TU are G+C-rich (Supplementary Figure S19B). However, already 1 h post-induction the different clusters bifurcated, and one G+C-rich cluster (1, red) became upregulated, while one A+T-rich cluster (3, green) became downregulated (Figure 6D, E). Next, we focused on the core promoter (Figure 7B–D, Supplementary Figures S19–S24) to query for previously described supercoiling-sensitive structural features (32,52). This revealed a distinctive feature of *Synechocystis* promoters, namely, a strong coupling of the TSS with an A-tract-based structural DNA code present in most bacterial genomes (47,51), but specifically pronounced in polyploid cyanobacteria such as *Synechocystis* (53). A-tracts of length four show a clear helically phased enrichment with the maximal peak at the −10 bp region of the promoter (Supplementary Figure S20A). This A-tract pattern can be further decomposed into a helically phased enrichment of the AT2 dinucleotide motif (ApA, ApT, TpT) and a localized enrichment of the complementary TpA step just upstream of the −10 peak of AT2, and again at the TSS (Figure 7B, C, Supplementary Figures S20B, C, S21); i.e., spanning the region of single-stranded DNA (open bubble) in the transcription initiation complex. Each cluster showed significant deviations from this common structure. The TSS-associated peak of the TpA step is most pronounced in cluster 2 (yellow). Cluster 3 (green) shows the lowest AT2 peak at −10 bp but the highest peaks up to −50 bp, covering the σ factor-binding region. In contrast, cluster 1 (red) shows the highest AT2 peak at −10 bp, but significantly lower peaks directly upstream. Periodic enrichments further upstream may be out of phase due to variable distances from the TSS. Indeed, the autocorrelation function of concatenated promoter sequences shows comparable amplitudes in all clusters, and with higher periods (>11 bp) in upregulated and lower periods in downregulated clusters (Supplementary Figure S22).

### The discriminator region and sigma factors

The deviations from this common structural pattern may be related to the differential immediate and adaptive responses of transcription to topA^OX^ induction. The GC content between the −10 element and the TSS, known as the discriminator region, partially determines positive and negative responses to both the regulatory metabolite ppGpp and to changes in DNA supercoiling (27,30,31,96,97). This pattern is consistently found in phylogenetically distant bacteria (32). We find two distinct nucleotide enrichment patterns in this region, one downstream and another one upstream of a conserved T at position −7 (Supplementary Figure S24A, B) which binds to a pocket of the σ^70^ factor of the *E. coli* RNA polymerase initiation complex (35,98,99). Firstly, the promoters that were upregulated 20 min post-induction show an enrichment of A+T nucleotides at −6 to −3 (Figure 7F, Supplementary Figure S19D,F, S24C). This is consistent with data from other bacterial species (32). However, it reflects the overall GC/AT bias of these promoters, extending beyond the core promoter and into the coding region. Secondly, cluster 1 (red) promoters are enriched in A between −7 and −11, upstream of the T at −7 (Figure 7D, E, Supplementary Figure S25).

Thus, the immediate response to topA^OX^ induction is largely consistent with responses observed throughout the bacterial phylogeny. The subsequent adaptive response likely reflects regulatory mechanisms specific to cyanobacteria or *Synechocystis*, and may involve specific sigma factors. We thus, investigated the expression patterns of the nine annotated sigma factors (100) (Supplementary Figure S26). The *sigA* transcript was downregulated quickly after topA^OX^ induction and was low in all endpoint measurements. The transcripts of group 2 sigma factors *sigB* and *sigC* were upregulated in all experiments but the time series shows that both are initially downregulated until 5 days post-induction. The group 2 sigma factors SigD and SigE are involved in circadian control, and their target genes partially overlap with those of SigA (101–103) (Supplementary Figure S26A). SigE activates sugar catabolic pathways during growth in light/dark conditions (104). Its transcript was down-regulated, but showed a diurnal pattern, slightly ahead (phase-advanced) of the diurnal pattern of cluster 3 (green) transcripts (Supplementary Figure S26C). The transcript of SigD was downregulated at all time points, but upregulated in the gyr^kd^ strains. And finally, the group 3 and 4 factors *sigH* and *sigI* are the only sigma factors that were upregulated upon topA^OX^ induction: *sigI* transiently over the first three days, and *sigH* in three circadian steps, slightly phase-advanced of the circadian pattern of cluster 1 (red) transcripts (Supplementary Figure S26C).

### Graded response along transcription units

Supercoiling does not only affect initiation but also elongation of transcription (105). Gyrase activity downstream of transcription units can resolve positive supercoiling that arises from the act of transcription itself (Figure 1B), and such sites are found, e.g., downstream of rRNA loci and highly transcribed operons in *E. coli* (21). Failure to remove downstream supercoiling leads to RNA polymerase stalling (19–21). Thus, we inspected the spatial fold-change patterns along TU in the batch culture RNAseq data (Figure 4) by analyzing the differences between the first and last transcribed gene of each multi-gene TU. Indeed, we find that the gyr^kd^ but not the topA^OX^ strains showed graded expression along TU (Figure 7G, H, Supplementary Figure S27). This mostly affected large (multi-gene) TU of the G+C-rich clusters 1, 2 and 4 (red, yellow, gray), is most pronounced in cluster 1, comprising of ribosomal protein genes, and is reminiscent of the graded effect at the rRNA loci (Figure 4A).

## DISCUSSION

We manipulated the expression of gyrase and TopoI genes in *Synechocystis*, and showed that increased DNA relaxing (topA^OX^ strain) or decreased DNA supercoiling (gyr^kd^ strains) activity inhibits cell division and broadly affects physiology. Our data largely confirm the prevailing models of the role of DNA supercoiling homeostasis in bacteria for *Synechocystis* (Figure 1). We further demonstrate a direct coupling of *Synechocystis* promoters to helically phased A-tracts.

### A toolbox for biotechnology and supercoiling research

Using the inducible dCas9-mediated CRISPR-interference system (64) we successfully repressed transcription of gyrase subunits *gyrA* and *gyrB*, or *gyrA* and *gyrB* simultaneously. Our tunable expression plasmid pSNDY (63) allowed us to over-express the native *topA*. All manipulations impacted pigment content, cell volume and ATP levels: pigments decreased whereas cell volume and ATP+ADP content increased. The most pronounced effects were observed for the strain topA^OX^, which contained more than twice as much glycogen, comparable to the levels in nitrogen-starved cells (106,107). The sigma factor SigE activates glycogen degradation genes during the diurnal cycle (104). Its downregulation may thus underpin glycogen accumulation in the topA^OX^ strain. SigD, a diurnal counterpart of SigE (101), was downregulated in topA^OX^ but upregulated in the gyr^kd^ strains. This may underlie some of the differences between the phenotypes. The enlarged cell volume, in all strains, was further confirmed by flow cytometry and microscopy, which additionally revealed an increase in the fraction of 8-shaped cells, suggesting a block in cell division but not growth. Thus, we successfully redirected cellular resources by manipulation of DNA supercoiling, providing a promising platform for photoproduction. A combination of our constructs into a single strain, towards a fully synthetic control over the endogenous DNA supercoiling homeostasis, may allow optimization of growth and production phases in photobioreactors. The higher transcript abundances from strongly supercoiled plasmids in the topA^OX^ strain may prove specifically useful to boost expression of exogenous genes, as integration sites for most plasmids have been suggested recently (108). As a next step towards a biotechnological chassis organism, our manipulation of topoisomerase expression must be assessed on protein abundance level. Protein stability of the targeted topoisomerases will likely have to be modified, e.g. by inducible degron systems, to allow for a rapid switch of DNA supercoiling at an optimal point during a production phase.

The *gyrB* knockdown strains gyrB^kd^ and gyrAB^kd^ showed increased ATP+ADP content, and only the gyrB^kd^ strain showed (slightly) increased plasmid supercoiling. We did not further investigate these differences. They could be related to an additional function of GyrB, together with the second GyrA-like protein in *Synechocystis* (*sll1941*) and potentially as a decatenating topoisomerase (ParC/D, TopoIV) (89,90), or may stem from the CRISPRi off-target *sll1625*, a succinate dehydrogenase (91,92). The single knockdown gyrA^kd^ strain showed the weakest metabolic phenotype and is therefore best suited for future studies into the dynamic response to supercoiling in *Synechocystis*.

### Evidence for the supercoiling homeostasis and the twin-domain models in *Synechocystis*

Overexpression of *topA* only transiently relaxed the plasmid DNA, and after ≈1 day, the plasmids became increasingly supercoiled. This overcompensation exemplifies the often counterintuitive consequences of manipulating a homeostatic feedback system. *In vitro*, hypernegative supercoiling of plasmids can be generated by gyrase and transcription (109). *In vivo*, it has been observed in a *topA*-deficient *E. coli* strain and depended on transcription (110,111). In our topA^OX^ strain, plasmid yields (per OD) and transcript abundances of plasmid-derived genes all increased with supercoiling. Transcript abundances of both gyrase subunits and the gyrase substrate, ATP, increased in parallel. The overexpression of *topA* may have triggered a positive feedback between plasmid transcription and/or replication and gyrase activity. Gyrase binding sites are frequently found in native plasmids and phage genomes (112) and such sites could contribute to this phenomenon.

Our other results are more consistent with previous observations. We observed compensatory upregulation of *topA* in the gyr^kd^ strains and of *gyrA* and *gyrB* in the topA^OX^ strain. Menzel and Gellert (1983) first suggested that transcription of the topoisomerase genes is under homeostatic control by negative feedback via the supercoiling status (2); and the same pattern is observed in many species across the bacterial phylogeny (2,6,12–15,113–115). Even the stronger response of *gyrA* than of *gyrB* (to a decrease of supercoiling) has been previously reported in *E. coli* (116). Likewise, the immediate genome-wide response to *topA* overexpression is consistent with reports from many species (10–16): genes with G+C-rich coding regions were downregulated and A+T-rich upregulated 20 min post-induction. In the gyr^kd^ strains the G+C-rich TU clusters showed a graded response along TU, such that the downstream gene showed lower upregulation or stronger downregulation than the upstream gene. To date, there is no clear explanation for the correlation between the G+C content and the differential immediate response to DNA relaxation. G+C-rich DNA requires more energy for melting of the double helix, due to three instead of two hydrogen bonds per base pair. Indeed, the *in vivo* elongation rate was lower in G+C-rich genes of eukaryotes (117,118). In bacteria, elongation depends on downstream gyrase activity to avoid build-up of positive supercoiling and RNA polymerase stalling, especially at strongly transcribed loci such as RP and rRNA genes (19,21). This requirement could specifically explain the graded effect along G+C-rich TU in the gyrase knockdown strains which was most pronounced in cluster 1 (red), as well as the stronger downregulation of the downstream 23S than the upstream 16S rRNA at the rRNA loci. In summary, our data suggest that both the homeostatic feedback control of topoisomerase transcription (Figure 1A), and the twin-domain model of transcription-dependent supercoiling (Figure 1B) also hold in *Synechocystis*.

### Helical phasing of the −10 elements and the TSS in *Synechocystis*

Already 1 h post-induction the transcriptional response diversified into at least six distinct groups of transcription units. Due to the quick compensatory reactions as well as the strong phenotype, we can not infer any causal models for this response. However, the six gene clusters overlapped with gene groups that were diurnally co-expressed (84,88) or responded differentially to growth rate (94). They may thus reflect physiologically relevant regulons (groups of TU with functionally interacting protein products). Their differential response correlated with significant deviations from a common promoter structure: a periodic enrichment of the AT2 motif, in-phase with the −10 element and the TSS. The AT2 motif is a minimal representation of short repeats of A and T nucleotides (A-tracts) without the TpA step (119,120). The TpA dinucleotide step is locally enriched just upstream of the AT2 peak at the −10 element (cf. ‘TATA box’), and again at the TSS. TpA is structurally distinct and has been called a twist capacitor, since it can adopt both high and low twist states in molecular dynamics simulations, and thereby locally absorb torsional stress (121). Here, at −10 and the TSS, this property could facilitate open bubble formation of the transcription initiation complex. A-tracts have a narrower minor groove of the DNA double helix (46,122,123), providing binding sites for arginine residues in proteins that wrap DNA (124), notably: gyrase (21,125,126), or locally pinning DNA loop (plectoneme) formation (54). Their helically phased enrichment is observed throughout all domains of life (47,53), and specifically also upstream of bacterial promoters (46,50,52,54,127). However, only a few anecdotal observations reflect the direct coupling that we observe in *Synechocystis*. For example, four helically phased A-tracts alone served as a promoter, and the most downstream A-tract served as the TSS in artificial constructs (38). The phasing of the A-tracts relative to the −35 region determined the efficiency of a bacteriophage promoter (37). Kravatskaya *et al.* (2013) found that alignment of promoter sequences at the TSS facilitates the detection of AA+TT dinucleotide periodicities in supercoiling-sensitive *E. coli* promoters (52). To our knowledge, we provide the first observation of a direct alignment of the TSS and the −10 element with helical phased A-tracts on a genome-wide scale. It is possible that cyanobacterial RNA polymerases (128) and σ factors (102) rely more on such DNA structural features than the well-studied *E. coli* case. However, strong genome-wide A-tract periodicities in some cyanobacteria, incl. *Synechocystis*, correlated with a polyploid life style (53) and could also serve efficient packaging of the multiple genomes into plectonemic structures (48). The pattern we observe at promoters could thus merely reflect the proper integration of such a genome packaging code with promoters, similar to its embedding into the first and third codon positions in protein-coding regions (53). These explanations are not mutually exclusive, and evolution could yield A-tract-aligned promoters when these A-tracts are also beneficial for genome packaging.

### Torsional strain and open bubble formation

Each time series cluster showed significant deviations from this common structure. Are these distinct patterns directly involved in the differential response to changes in DNA supercoiling? Several non-exclusive models how DNA supercoiling can affect transcription initiation have been proposed (5,29,32,39,40,42,52,129–131). A-tracts can locally stabilize DNA plectonemes, and such DNA loops can suppress *lac* operon promoters if positioned correctly (132,133). Notably, the dependence on a correct phasing of repressive motifs with the −35 element was stronger in *Synechocystis* than in *E. coli* (134). RNA polymerase can bind to the apical loop of a plectoneme and shifts this loop during transcript elongation, thereby avoiding rotation around the template (135). It was suggested that the RNA polymerase channels the torsional strain that is stored in the plectonemic structure into the opening of the DNA double helix between ca. −12 and +1 of the TSS (5,131), with differential supercoiling-dependence of A-tract periods shorter or longer than the DNA helical pitch (≈10.5 bp) (40,52). The auto-correlation analysis did reveal subtle differences in AT2 motif periods (Supplementary Figure S22), but a significance of these differences remains to be shown.

The sequence-dependent stability of the open bubble conformation of the RNA polymerase (open complex) determines the promoter’s response to both ppGpp and DNA supercoiling (30–32,97). For example, the stability is affected by the GC content between the TSS and the −10 element, a region therefore known as the discriminator (27,96): a higher GC content correlates with both supercoiling-dependence and ppGpp repression of promoters. These differences are observed in a variety of bacterial species (32), including *S. elongatus*. We found a consistent pattern in the promoters affected 20 min after induction of topA^OX^, specifically A+T are enriched from −6 to −3 bp of the TSS, just downstream of a conserved T at position −7, in upregulated (relaxation-induced) promoters. In contrast, the time series cluster 1 (red) was enriched in A from −12 to −7. In *E. coli*, the T at −7 is flipped out of the helix and bound to a pocket of domain 2 of the σ^70^ factor during open complex formation (35,98,99). The discriminator is bound by the domain 1.2 of σ^70^ (96). All sigma factors that contain domain 1.2 (group 1 and group 2, (136)) were downregulated during the adaptive response. Only *sigH* and *sigI* were upregulated, and the latter with a circadian pattern. The *sigI* transcript was also upregulated during the dark phase of the diurnal cycle (84,137). The lack of domain 1.2 of these group 3 and 4 sigma factors may weaken the dependence of the promoters on supercoiling. And finally, differential enrichment of TpA at the TSS may point to a role of this twist capacitor dinucleotide during open complex formation (121).

### DNA supercoiling and the diurnal program

Despite significant differences of the cyanobacterial core transcription infrastructure (102,138,139), ppGpp has very comparable consequences on transcription in cyanobacteria (59,60). Its increase is directly associated with the transcriptional shut-down during dark periods (60), and, during the light phase, it may modulate the diurnal transcription program (61). By inference from the roles of supercoiling and ppGpp in other bacteria, we can suggest a tentative model for the observed changes in gene expression upon *topA* induction or gyrase knockdown: in our constant light experiments ppGpp was likely low, while overexpression of *topA* shifts the supercoiling homeostasis and DNA structure towards the opposed physiological state, usually encountered during the dark phase. This combination, low ppGpp and low supercoiling, could reflect the dark/light transition during the diurnal cycle, and induce the expression of the dawn-specific cluster 1 (red), comprising of growth-relevant genes such as ribosomal proteins and the RNA polymerase. Indeed, the increase of translation-related transcript abundances started shortly before the actual onset of light in *Synechocystis* (84). In physiological context, strong transcription of this cluster would require downstream gyrase activity, and this transcription would lead to an overall increase in genomic supercoiling, according to the twin-domain model. This increase in genomic supercoiling in turn could be required to progress through the temporal expression program, and to initiate dawn-to-noon DNA replication (140). The promoters of clusters 2 and 3 (yellow and green), overlapping with noon-specific and dusk-specific cohorts, show a coherent helical phasing of the A-tract motif up to at least -60 bp which may specifically mediate sensitivity to the local or global level of supercoiling. In our experiments, increased TopoI or decreased gyrase activity would inhibit this transcription-dependent accumulation of supercoiling. The diurnal transcription program would be stuck in a dawn-like state, the genome would not be replicated and cell division blocked.

As an outlook, our strains should next be studied in diurnal conditions. A spatially resolved analysis of transcription along the genome (81), as well as DNA-structural footprinting methods, e.g., mapping of supercoiling-sensitive psoralen-binding sites (141), of gyrase-cleavage sites (21) or of the core transcription machinery (142) will provide an integrative picture of global regulatory mechanisms in a physiological context.

## DATA AVAILABILITY

The clustering and time series data from the topA^OX^ strain (both as raw abundances in TPM and as the log2 ratios to the mean of two pre-induction values, as plotted in this manuscript), and endpoint measurements (log2 ratio of abundances in the gyrA^kd^, gyrB^kd^ and topA^OX^ strains to the EVC strain) are available as Supplemental Data File S1 (file Datatable_S1.tsv). The RNA-seq raw data have been deposited in the ArrayExpress database at EMBL-EBI (www.ebi.ac.uk/arrayexpress) under accession number E-MTAB-10949. The annotated sequence of the pSNDY plasmid, is available as Supplemental Data File S2 (genbank file pSNDY_Prha_topA-6_119rhaS.gb).

## Supplementary Material

gkac1132_Supplemental_Files
