# Supplementary material for: Manipulation of topoisomerase expression inhibits cell division but not growth and reveals a distinctive promoter structure in Synechocystis
Source: Nucleic Acids Res. 2022 Dec 19;50(22):12790–808. doi: 10.1093/nar/gkac1132 (PMC9825172; doi:10.1093/nar/gkac1132)
Supplement: gkac1132_Supplemental_Files [file gkac1132_supplemental_files.zip › supplement_v4.pdf]

Supplementary Information for Behle, Dietsch, *et al.* (2022): Manipulation of Topoisomerase Expression Inhibits Cell Division but not Growth, and Reveals A Distinctive Promoter Structure in *Synechocystis*.

Strain name	Chromosomal genotype	Plasmid
EVC	P_{L22}:dCas9	pSNDY (EVC)
gyrA^{kd}	P_{L22}:dCas9; P_{L22}:sgRNA$_{gyrA}$	pSNDY (EVC)
gyrB^{kd}	P_{L22}:dCas9; P_{L22}:sgRNA$_{gyrB}$	pSNDY (EVC)
gyrAB^{kd}	P_{L22}:dCas9; P_{L22}:sgRNA$_{gyrA}$; P_{L22}:sgRNA$_{gyrB}$	pSNDY (EVC)
topA^{kd}	P_{L22}:dCas9; P_{L22}:sgRNA$_{topA}$	pSNDY (EVC)
topA^{OX}	P_{L22}:dCas9	pSNDY P_{J23119}:*rhaS*; P_{rha}:*topA*

Table S1. Construction of strains investigated in this work. The parental strain for all strains listed here (*Synechocystis* sp. PCC 6803 encoding aTc-inducible dCas9) is based on a strain originally obtained from Martin Fulda (Göttingen, Germany) and engineered for CRISPRi by Yao *et al.* [1]. It contains a TetR cassette, as well as dCas9 under the promoter PL_{22}, inducible with anhydrotetracycline (aTc), at the genomic insertion site *psbA1*. The sgRNA sequences (Tab. S2) were constructed *via* overlap extension PCR and integrated into the vector designed by Yao *et al.* [1] (Addgene ID 73224), which inserts into the *slr0230* site of the *Synechocystis* genome. The sgRNA plasmids were integrated *via* transformation. Briefly, 10 mL of exponentially grown culture was concentrated to 250 µL, 1 µg–2 µg of pure plasmid was added and the mixture was incubated up to 5 h before plating the entire mixture on BG11 plates. After drying the plates, agar was underlaid with 300 µL of 1 mg mL^{-1} kanamycin stock using a sterile spatula, thereby forming a diffusion gradient. After 1-2 weeks of incubation at 30 °C with the lid facing upward, isolated green colonies were carefully transferred to a fresh plate. Over time, positive clones were gradually shifted to higher concentrations of kanamycin (4, 8, 12, 20, 40 µg mL^{-1} final concentration in the plate). Complete segregation of mutants was ensured *via* colony PCR. For rhamnose-inducible overexpression, the coding sequence of *slr2058* (*topA*) was integrated into pSHDY P_{J23119}:*rhaS*; P_{rha}:*mVenus* (Addgene ID 137662) [2] in place of *mVenus via* Gibson assembly. Both this new construct and pSHDY (Addgene ID 137661) were further modified by exchanging the spectinomycin resistance cassette with the nourseothricin cassette, resulting in pSNDY P_{J23119}:*rhaS*; P_{rha}:*topA* and pSNDY (EVC), respectively. Replicative vectors were introduced into the dCas9 background strain *via* conjugation as described (dx.doi.org/10.17504/protocols.io.ftpbnmn). Clones were selected using nourseothricin (Jena Bioscience, #AB-102L) at a final concentration of 50 µg mL^{-1} and verified *via* colony PCR.

Gene	Protospacer Sequence	Predicted Off-Targets
gyrA	TCAGTCATGCAATTACTCCA	*ssr3154*, *slr1560*
gyrB	ACTCCAAAATCAGGCTGAGCTT	*sll1625*, *slr0896*
topA	GATAGTGCGGGCTTTAGTGG	*sll1660*

Table S2. sgRNA Protospacer Sequences. The target-specific parts (protospacer) of the sgRNA for CRISPRi-based knockdown strains (Tab. S1) were designed using CHOPCHOP [3]. and potential off-targets analyzed by the Cas-OFFinder [4], allowing ≤ 2 mismatches or DNA/RNA bulges. Only the *topA* sgRNA had had one additional target (in gene *sll1660*), but this strain was not further analyzed beyond Figure 2. Furthermore, Cui *et al.* [5] found that a 9-nt identity of an an off-target site to the sgRNA seed sequence (seed sequence = first 12 nt) can result in significant repression, if an NGG PAM site is present and that the sgRNA anneals to the coding strand. We searched for such potential off-target sites for the three sgRNAs used here. The *topA* sgRNA does not meet this criteria for any other sites. The *gyrB* sgRNA meets this criteria for two off-target sites, *sll1625* and *slr0896*. The *gyrA* sgRNA meets this criteria for two off-target sites in the genome, *ssr3154* and *slr1560*. These off-targets were included in the RNAseq analysis in Figure 4D.

Gene	L_A	T_m	Primer efficiency	Direction	Sequence
gyrA	102	82.9℃	104.16%	forward	GAACTTTGGCTCCGTGGATAA
				reverse	GCCTCAATGTCCCGCAATAA
gyrB	107	78.9℃	100.32%	forward	TGCCCGTAAGCGCAATAA
				reverse	ATTCTGGGTCCGGTACTTTAAC
topA	105	80.6℃	99.43%	forward	AGACCGGGAAGGAGAAAGTA
				reverse	CGAATGGCTTCCTGGGTAAT
rpoA	96	80.4℃	97.86%	forward	CCATGAGTTCGCCACTATTCT
				reverse	GGCTGATCGGTGTAGCTTT
rnpB	93	82.04 ℃	98.29%	forward	AGAGGTACTGGCTCGGTAAA
				reverse	TCAAGCGGTTCCACCAATC

Table S3. RT-qPCR Primers. Primers used for RT-qPCR of the indicated genes were designed using the IDT PrimerQuest tool (`https://eu.idtdna.com/pages/tools/primerquest`). L_A: amplicon length, T_m: melting temperature

Name	RefSeq ID
genome	NC_000911
pCA2.4_M	CP003270
pCB2.4_M	CP003271
pCC5.2_M	CP003272
pSYSM	NC_005229
pSYSA	NC_005230
pSYSG	NC_005231
pSYSX	NC_005232
pSNDY P_{J23119}:*rhaS*; P_{rha}:*topA*	file `pSNDY_Prha_topA-6_119rhaS.gb`

Table S4. Genome and Plasmid Sequences for RNA-seq Mapping. RefSeq IDs of the genome and plasmid sequences of *Synechocystis* used for mapping of the RNA-seq reads. Construction of pSNDY is described in Table S1 and the full sequence is available as Supplemental Data File S2 (genbank file `pSNDY_Prha_topA-6_119rhaS.gb`).

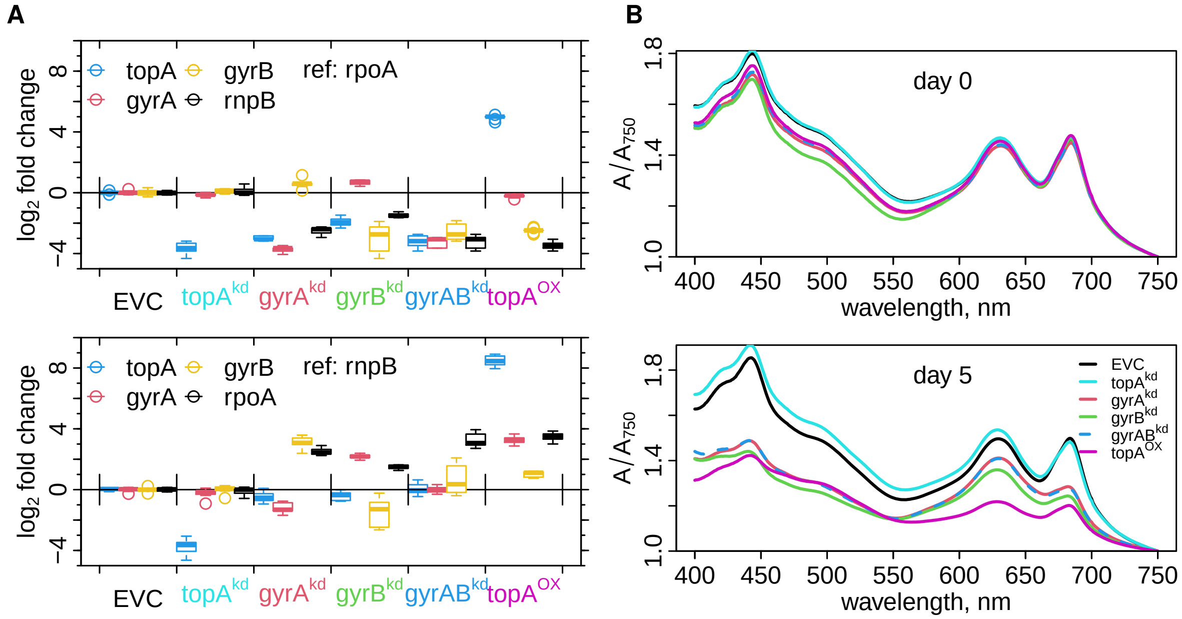

Figure S1. Batch Culture Endpoint Measurements. See Figure 2 for details. **A:** RT-qPCR results using *rpoA* (top panel) or *rnpB* (bottom) as reference genes [6]. Boxplots of 9 technical replicates (3 samples, each measured 3x) . **B:** Absorption spectra at inoculation and harvest times.

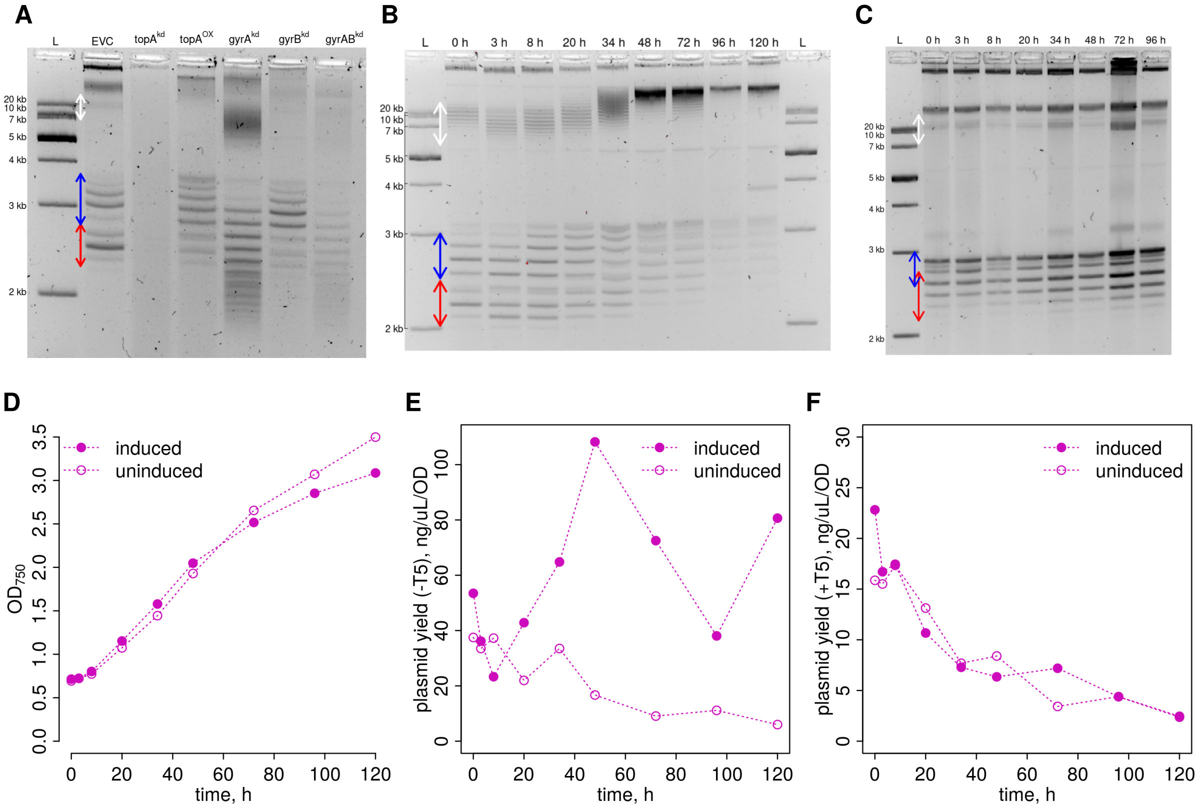

Figure S2. Plasmid Supercoiling Gels. A–C: Chloroquine-agarose gels 1.2 % agarose, 0.5x TBE and 20 µg mL^{-1} CQ, 1.8 V cm^{-1}) of plasmids extracted from all strains at harvest time (A) of the experiment shown in Figure 2, or as a time series (see D–F) of the topA^{OX} strain with (B), or without the inducer (C). At 20 µg mL^{-1} CQ, originally more relaxed (rel.) plasmids migrate further (higher migration distance) than more negatively supercoiled plasmids [7]. Two distinct plasmid topoisomer distributions can be distinguished in the EVC in gel (A), run for 20 h. We assume the less far migrated bands to correspond to the larger plasmid pCA2.4_M (2378 bp, blue arrows), and the further migrated bands to the smaller plasmid pCB2.4_M (2345 bp, red arrows). The size of a larger plasmid, pCC5.2_M (5214 bp) fits to a series of topoisomer bands indicated by white arrows. Bands above that likely stem from our pSNDY and or larger endogenous plasmids of *Synechocystis*. The gel of the induced culture (B) was run for a longer time (22 h) to get a better separation of the pCC5.2_M topoisomers. The gel in (C) was only run for 18 h. Baseline-corrected electropherograms of the gels in (A) and (B) are shown in Figure 2B and 2C of the main manuscript, respectively. **D:** Growth of the topA^{OX} strain, with or without induction with 1 mM rhamnose at time 0 h. For both conditions, a starter culture was split into 9 cultures at 0 h, each harvested at the indicated time points for OD_{750} measurement and plasmid extraction (used for electrophoresis shown in (B) and (C)). **E & F**: Yields of plasmid extraction over time, each normalized to the OD_{750} (D), and before (E) and after (F) treatment with the T5 exonuclease to remove all non closed circular DNA.

A

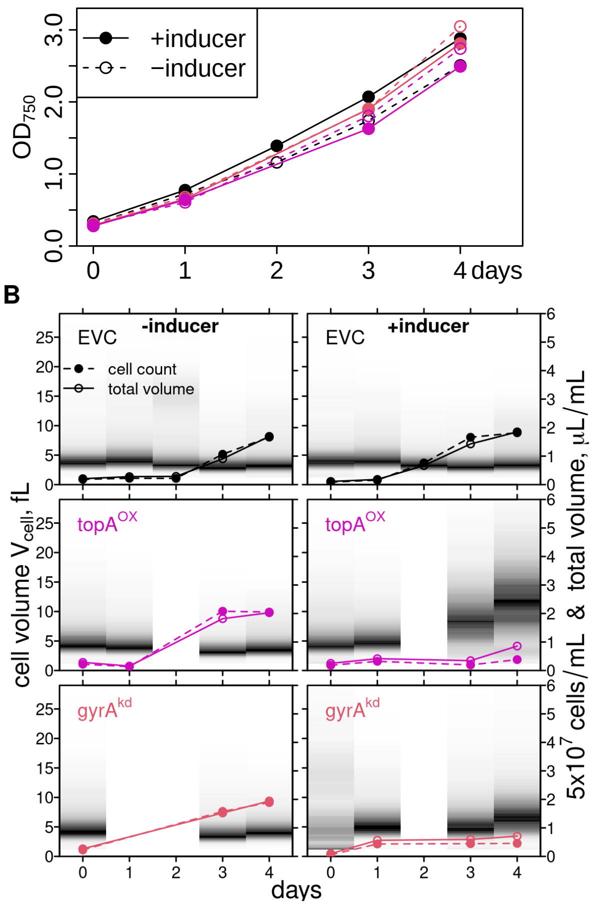

C

strain	ind.	images	cells	% 8-shaped
EVC	+	5	135	10.37
EVC	-	4	795	9.81
gyrA^{kd}	+	4	108	20.37
gyrA^{kd}	-	3	159	8.81
topA^{OX}	+	4	172	16.28
topA^{OX}	-	5	396	9.34

D

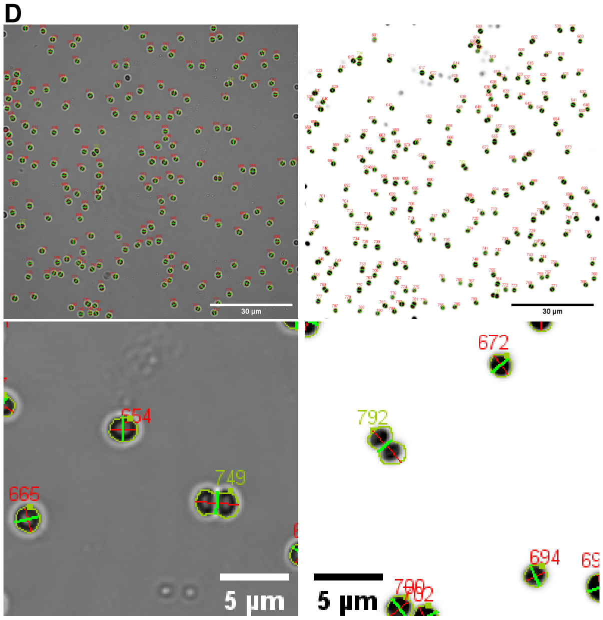

Figure S3. Batch Cultures for Microscopy. OD (**A**) and CASY cell counts and size distributions (**B**) from cultures of the indicated strains in the presence or absence of the inducer. Samples for microscopy were taken on day 4. **C:** Number of microscopy images and individual cells analyzed with the `Coli-Inspector` [8] (results in Fig. S5) and manual counts of the fraction of 8-shaped cells. The example cells in Figure S4 were also chosen from these images. **D:** Example image illustrating the semi-automated counting procedure. Each cell recognized by the `Coli-Inspector` is characterized by a length (thin red line) and width (thicker green line) parameter, where the shorter of both is always the width. For cell dimension measurements (Fig. S5A-C) the channel for chlorophyll fluorescence was used (right). Because of variations of focal planes those measurements were more reliable than those of the bright field channel (left). The fractions of 8-shaped cells (Fig. S5D, E) were manually counted in the brightfield images.

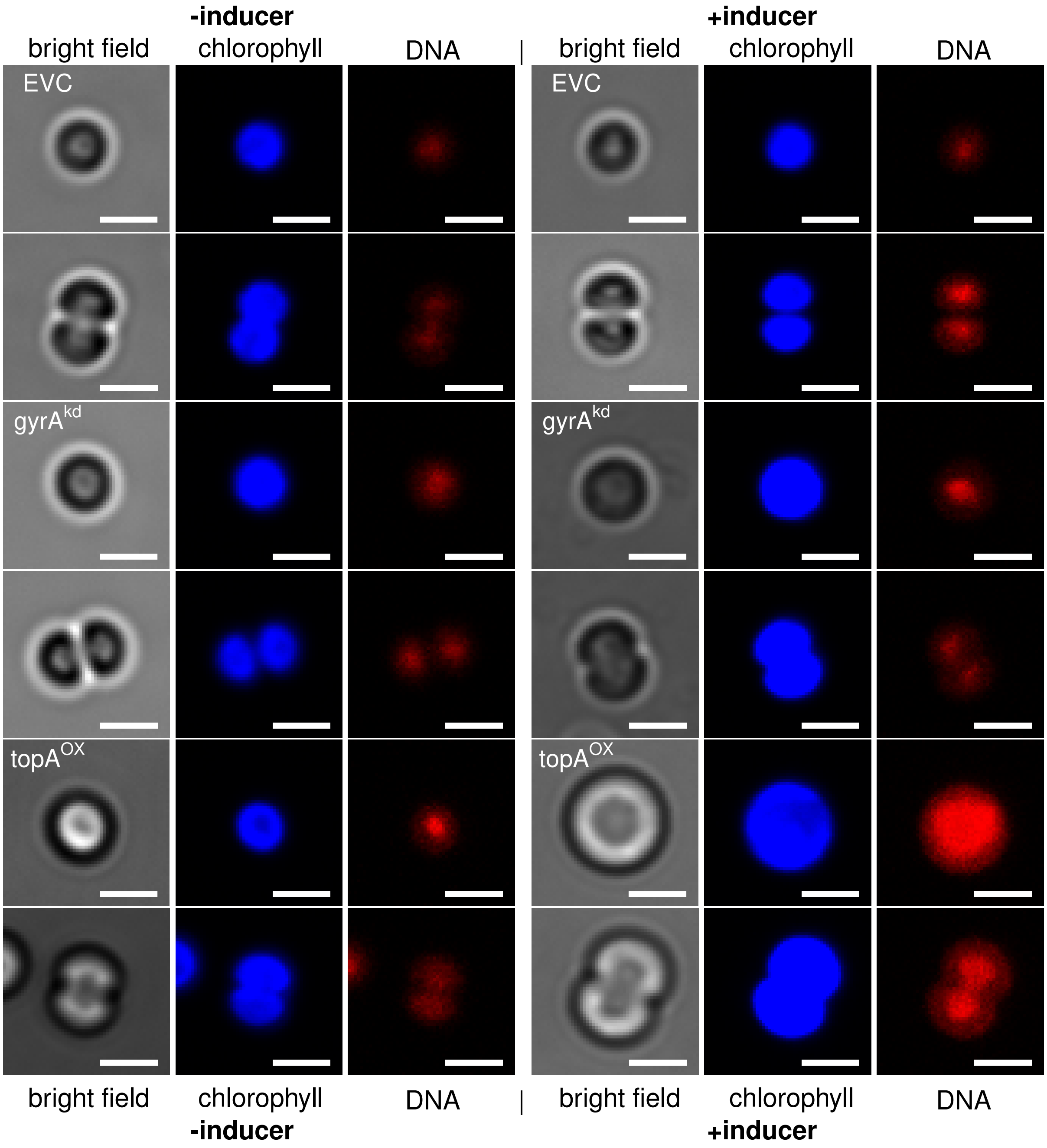

Figure S4. Batch Culture Endpoint Measurements for Microscopy. Zoom on typical single cells from the microscopy images of the cultures shown in Figure S3. The size bar length is 2 µm. From each strain and condition (± inducer) a typical round cell and a typical 8-shaped cell in division was chosen, and its brightfield (left panels) and chlorophyll- (blue, middle) and HOECHST DNA stain-specific (red, right) fluorescence channels are shown. Merged versions of these images, showing both fluorescence channels for the topA^{OX} and gyrA^{kd} strains with and without inducer, are shown in Figure 3.

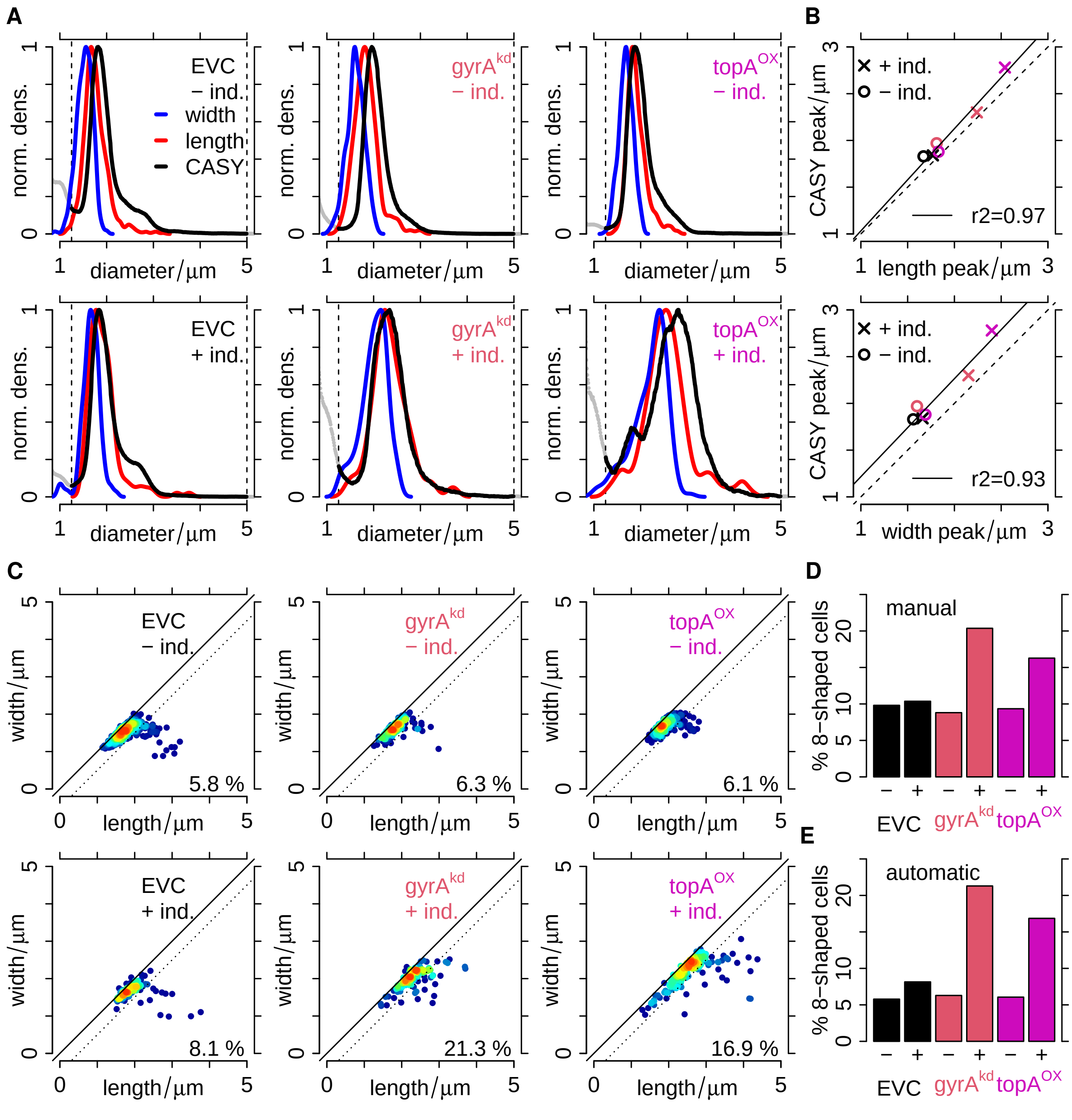

Figure S5. Microscopy Statistics and CASY Calibration. A: Density distributions of the cell diameters (black lines) measured by the CASY cell counter and cell lengths and widths (blue and red lines) determined by the `Coli-Inspector` [8] from microscopy images (Fig. S3C,D). All distributions were normalized to a maximum of 1. The dashed lines are the cut-off used for CASY data. **B**: Comparison of peak cell diameters as measured by the CASY cell counter and from microscopy images. The dashed line is the diagonal, and the solid line is a linear regression line, with the r^2 value shown in the bottom right legend. For microscopy, the peaks of the distributions were directly obtained from the density distributions shown in (A), and for CASY we used the peaks reported by the CASY. The CASY-based peak diameters are slightly larger than microscopy-based cell length and width peaks. **C:** cell-wise comparison of width and length estimation by the `Coli-Inspector` (with manual adjustments). For round cells width and length correspond well, for 8-shaped cells (in division) the width negatively correlates with length, since cells further into division are longer but the width, corresponding to the division plane, is shorter. The dotted line is the diagonal $-0.5\,\mu\text{m}$, and the cell fraction below this line, with width $<$ length $-\ 0.5\,\mu\text{m}$, was counted (bottom right text). **D & E:** Percent 8-shaped cells of all counted cells. 8-shaped cells were manually counted (**D**, see table in Fig. S3C) or determined automatically (**E**) by a difference in widths and lengths reported by the `Coli-Inspector` (see dotted line and % in (C)).

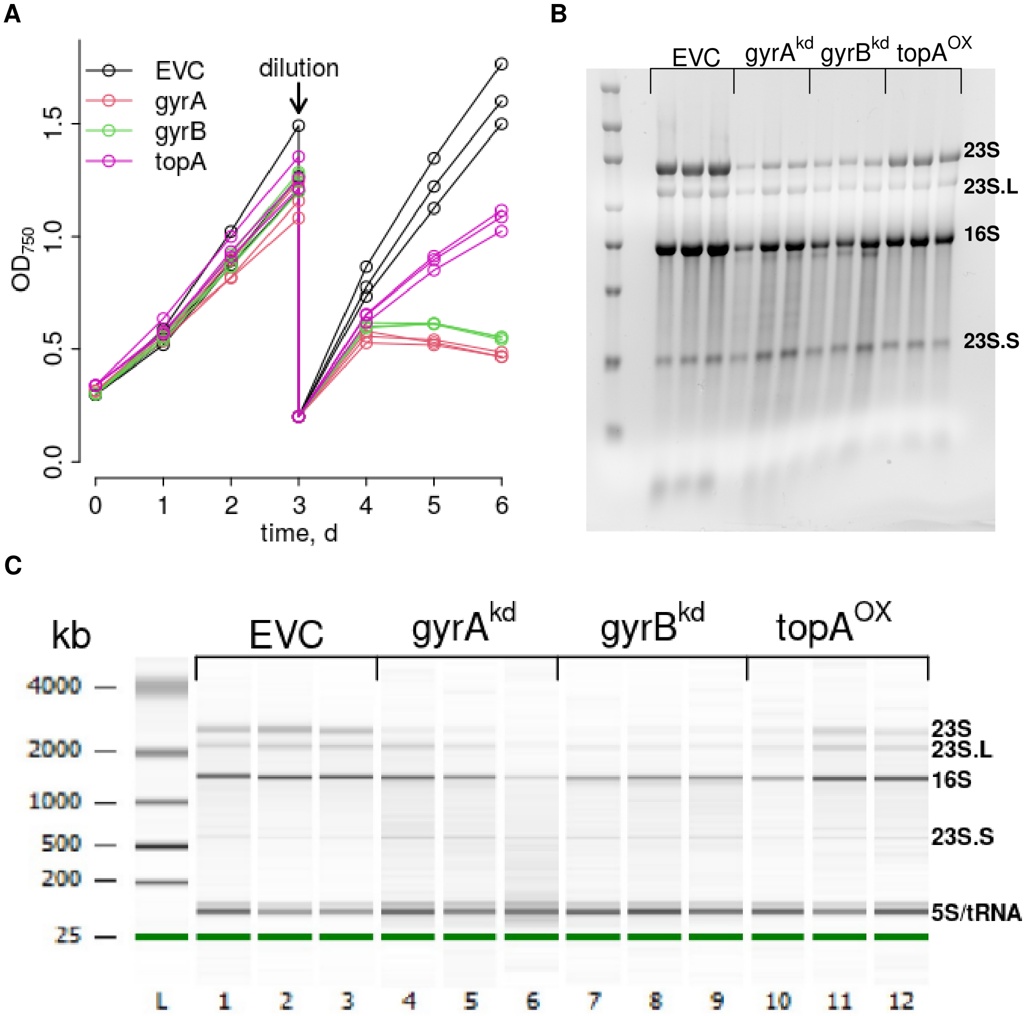

Figure S6. RNA Extraction for RNA-seq Experiment. A: Growth of triplicate cultures (split upon induction at 0 d).**B & C:** Total RNA compositions were analyzed by formaldehyde-agarose gel electrophoresis (B, 500 ng RNA per well) and by capillary gel electrophoresis (Agilent Bioanalyzer); the electropherograms of the pseudo-gel figure in (C, from the Bioanalyzer report) samples were analyzed for Figures 4A, S8 and S9.

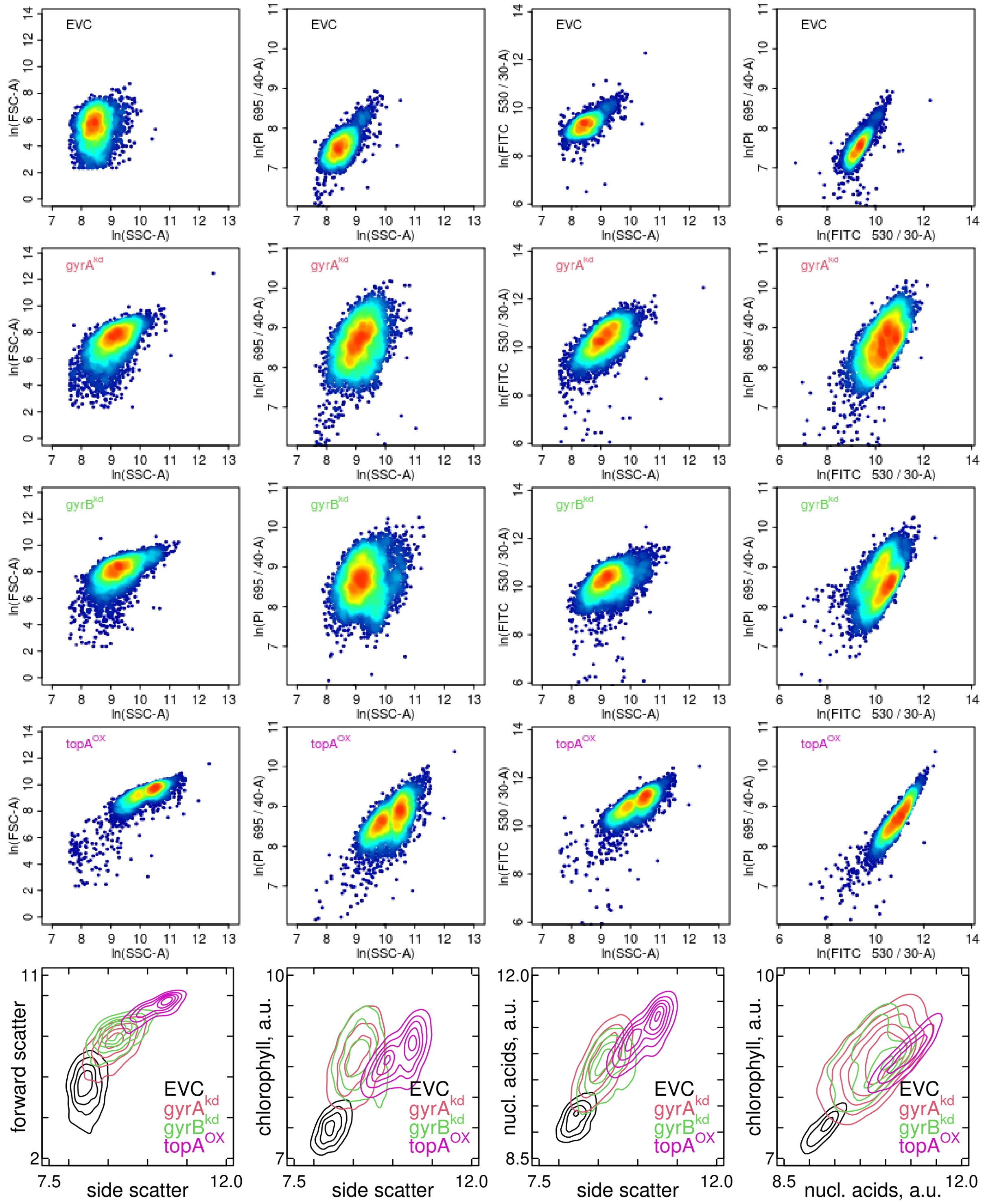

Figure S7. Flow Cytometry. Cells from the cultures used for RNA-seq and total RNA analysis (Fig. 4) were fixed in para-formaldehyde and stained with Syto-9, a nucleic acid fluorescence marker, and analyzed by flow cytometry. The data were gated by the side scatter signal (SSC-A> 2000) and the forward scatter signal (FSC-A> 10) to filter debris and background signals. Forward scatter (FSC-A) is proportional to cell size, side scatter (SSC-A) reflects cytoplasmic granularity and morphology; the FITC fluorescence channel (530/30 nm) excites the Syto-9 stain, and the PI channel (695/40 nm) excites chlorophyll. The natural logarithm ($\ln$) of all data was plotted. Colors reflect local density (red: high, blue: low). The bottom panels show a zoom into the data, and local densities are displayed as contour lines, merged for all strains.

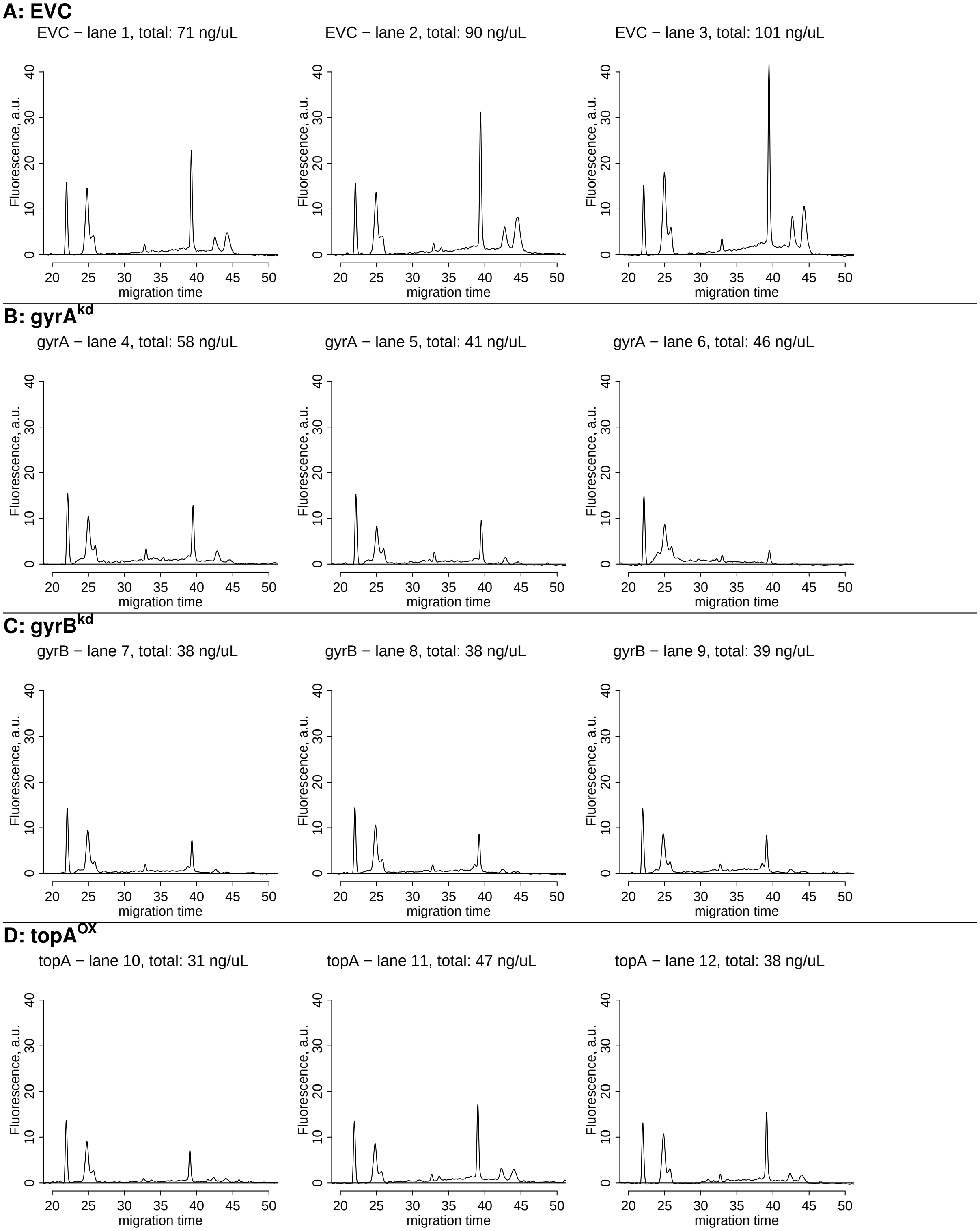

Figure S8. Raw RNA Electropherograms. Electropherograms of the capillary gel electrophoresis (Fig. S6C), exported as XML files from the 2100 Bioanalyzer software, and imported to R with the `bioanalyzeR` R package (v 0.9.1, `https://github.com/jwfoley/bioanalyzeR`) [9]. **A–D:** are each the triplicate samples for the indicated strains. Samples are the same as shown on the formaldehyde-agarose gel in Figure S6B and subsequently used for RNAseq analysis (Fig. 4B,C). The locations of rRNA peaks are indicated; r23S.S and r23S.L are short and long fragments of the 23S rRNA typically seen in *Synechocystis*, the other peaks were assigned to 5S, 16S and (full length) 23S rRNA. The total RNA concentration in the samples, as provided by the 2100 Bioanalyzer software report, are indicated on top of each plot, and these numbers were used for normalization of the electropherograms for Figures S9 and 4A.

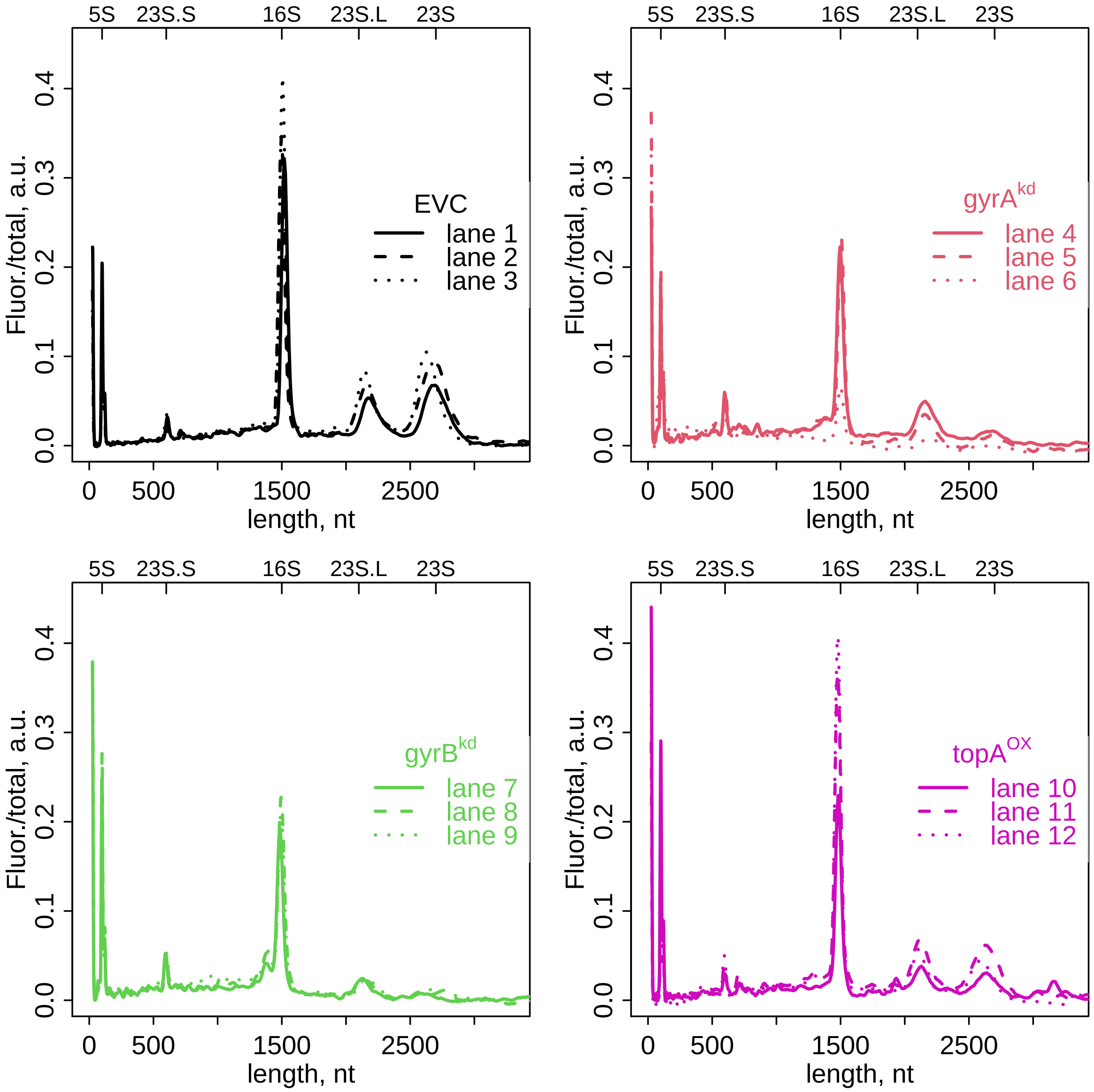

Figure S9. rRNA Relative Abundances. The RNA electropherograms in Figure S8 were each normalized by the total RNA content of the sample, as reported by the Bioanalyzer 2100 software, and plotted by replicate groups. The mean of each replicate group was calculated and plotted for Figure 4A.

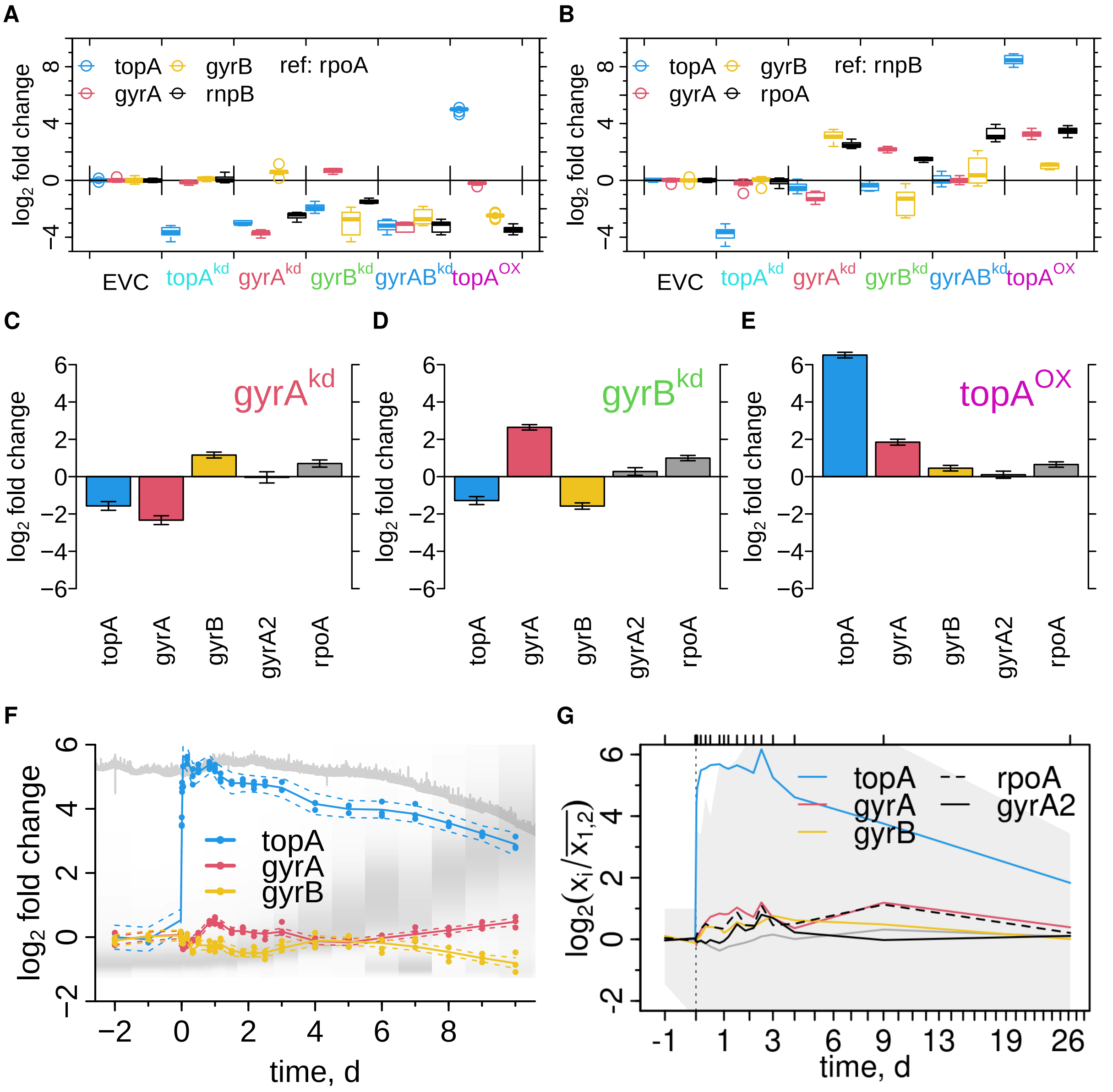

Figure S10. Homeostatic Regulation of Topoisomerase Genes: RT-qPCR vs. RNA-seq. **A and B:** RT-qPCR results from the endpoint experiments, reproduced from Figure S1A for comparison. **C-E:** Transcript abundance of topoisomerase genes and the reference gene *rpoA* used in qPCR (A,B) in the RNAseq data (Fig. 4, S6). *gyrA2* denotes the gene *sll1941*, a homolog of *gyrA*. **F-G:** The log2 fold change over the mean of the two pre-induction samples as measured by RT-qPCR (F) and RNAseq (G). RNAseq data shows that the expression of the reference gene *rpoA* increased with a periodic pattern and at a slightly higher fold change than *gyrB*, while *gyrA* stayed above or at similar levels as the reference gene. Considering this normalization effect (cf. [10]) the apparent downregulation of *gyrB* in (F) is an artifact of the reference gene, and the RT-qPCR and RNA-seq time series data are consistent.

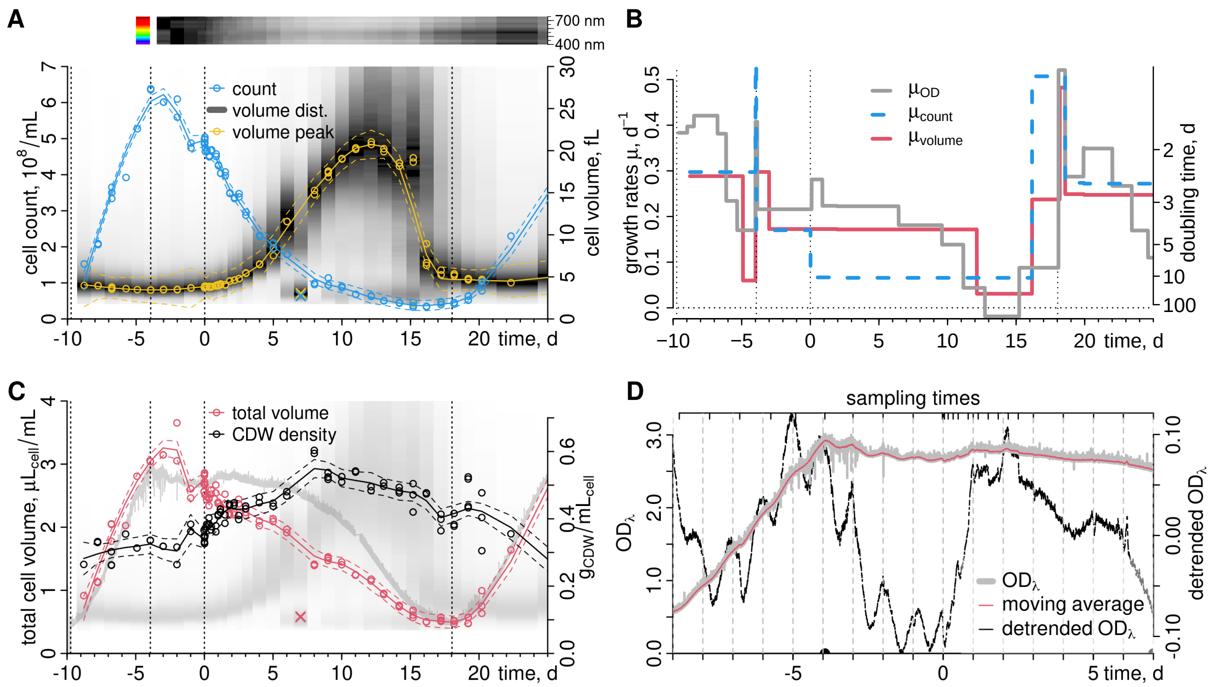

Figure S11. Pulsed Induction in Continuous Culture. Detailed biomass and growth rate data for Figure 5. **A:** Cell numbers (blue points) and volume distributions (gray scale) were recorded daily, and at higher resolution after induction, with the CASY cell counter. The peaks of the cell volume distributions are shown as yellow points. LOESS regressions with 95% confidence intervals are shown as lines. One outlier of the CASY measurement (x) was due to cell lysis during a washing step and was not included for regression. The top panel shows daily recorded spectra in gray-scale, where black indicates higher and white lower absorption. The underlying spectra were consistent with those of the batch culture shown in Figure S1.. **B:** The total cell volume (V_{total}) was calculated by integrating the single cell volume distributions in (B), and the CDW density were calculated by dividing the OD_λ signal, calibrated to the CDW measurements (A, Fig. A2C), by V_{total}. **C:** growth rates μ were calculated by local (piecewise) linear regressions of the OD_λ (A), and cell count (B) and total cell volume (D) measurements and subtraction of the culture dilution rate (Fig. A3). **D:** Detrending of the OD_λ signal for wavelet analysis (Fig. 5B). A moving average (red) of the raw signal by the OD probe (gray, time resolution: 30 s) was calculated using a window size of 30 min. The signal was detrended using the `detrend` function of the `pracma` R package (v2.3.6), where the end of the batch phase was used as the breakpoint (half bullet point on the bottom x-axis at ≈ -4 d). This subtracts two linear least-squares fits (before and after the breakpoint) from the data. This detrended signal was then used for Wavelet analysis with the `analyze.wavelet` function of `WaveletComp` R package (v1.1) for periods 0.24 h–96 h, and with `loess.span=0`, and `dj=0.05` and the mean time resolution of `dt=30` s. The dashed vertical lines indicate full days around induction as a 24 h reference. Sampling daily before and in higher resolution after induction (time 0 h) may have induced or removed the $\approx$ 24 h pattern, and sampling times are indicated on the top axis. Note, that daily sampling times shifted while the detrended OD peaks remained at constant times.

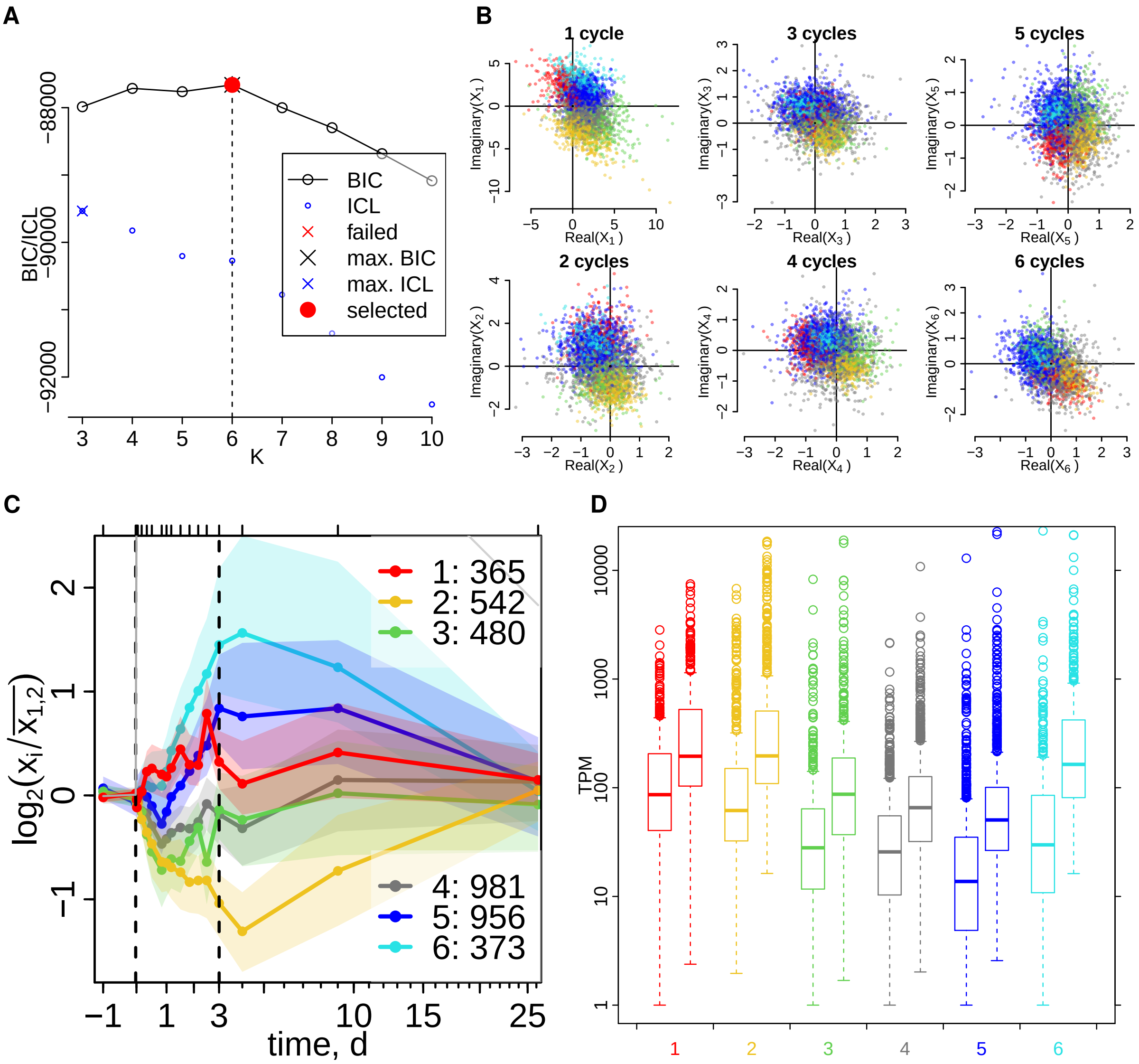

Figure S12. Clustering & Total Read-Count Distribution. A: Bayesian Information Content (BIC) as reported by `flowClust` for clustering of selected scaled components $X'_{k=1,\dots,6}$ of the Discrete Fourier Transform (DFT) of the `arcsinh`-transformed TPM data over varying number of cluster centers (K). The maximal BIC was reached for a classification into $K = 6$ distinct clusters (co-expression cohorts). This clustering was chosen for further analysis. **B:** real and imaginary parts of the DFT that were used for clustering (R package `flowClust` [11]). Colors already indicate the final cluster assignments of each transcript at $K = 6$ (A). **C:** Cluster medians (solid lines) of the relative transcript abundances (rel. abund.). For each transcript the $\log_2$ of the ratio of read-counts at time points i to mean of the two samples before induction ($i = 1, 2$, at -1 d and -1 h) was calculated (points indicate the sampling time points i). The transparent ranges indicate the 25% and 75% quantiles of each cluster. Only the time points within to two vertical lines were used for clustering. **D:** Cluster-wise distributions (boxplots) of minimal (left) and maximal (right) read-count values (TPM) of each transcripts.

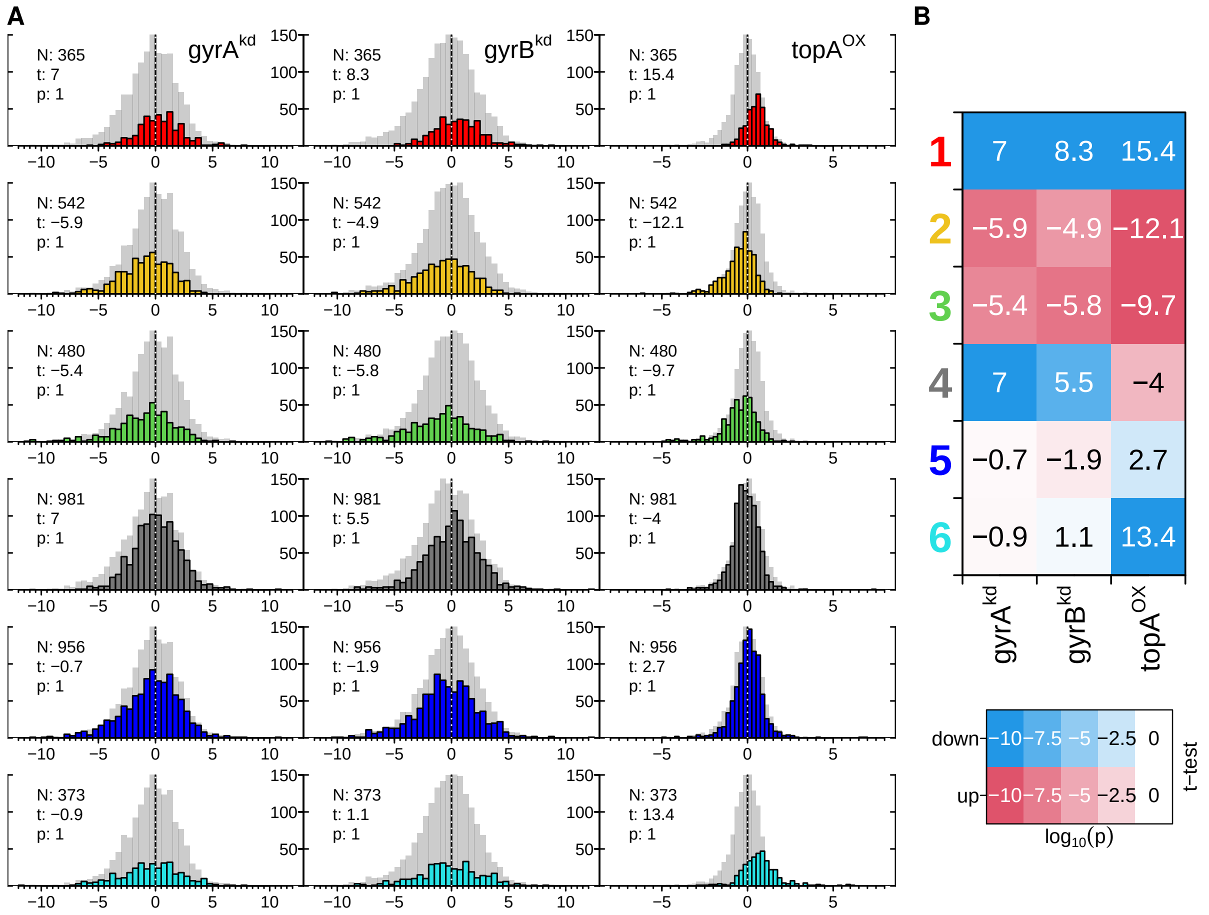

Figure S13. Co-Expression Cohorts in the Endpoint RNA-seq and Construction of t-Test Profiles. A: Distributions of the $\log_2$ fold-change of transcript abundances in the three strain endpoint experiment for each of the co-expression clusters derived from the topA^{OX} time series data. The gray background shows the distribution of all other transcripts. The y-axis are the counts for the colored distributions, while the gray background distributions are densities (without axis). For each cluster a t-test was performed (base R function `t.test`) against all transcripts not in the cluster, and the cluster sizes n, and the t-values and the p-values from each test are shown in each plot. The total number of transcripts with expression values were 3676 for gyrA^{kd} and gyrB^{kd}, and 3680 for topA^{OX}. **B:** A t-test profile plot is constructed from the t-test results in (A). A negative t-value indicates that the tested cluster transcripts have a lower mean abundance than all other transcripts and this is indicated by a red color field, the rounded t-value is shown in the fields; blue indicates a positive t-value and higher mean abundance. The p-value is converted to a transparency value for the red and blue colors (along a color palette from red/blue to white), such that the full color is reached for $p \leq p_{\text{min}}$, and for higher p-values the transparency scales with $\log_2(p)$. Both, for visibility of the text and to indicate an additional p-value cut-off the text (t-values) is plotted in white if $p \leq p_{\text{text}}$. The bottom legend shows 5 p-values (text: $\log_{10}(p)$) and the resulting field and text colors. Here $p_{\text{min}} = 10^{-10}$ and $p_{\text{text}} = 10^{-5}$.

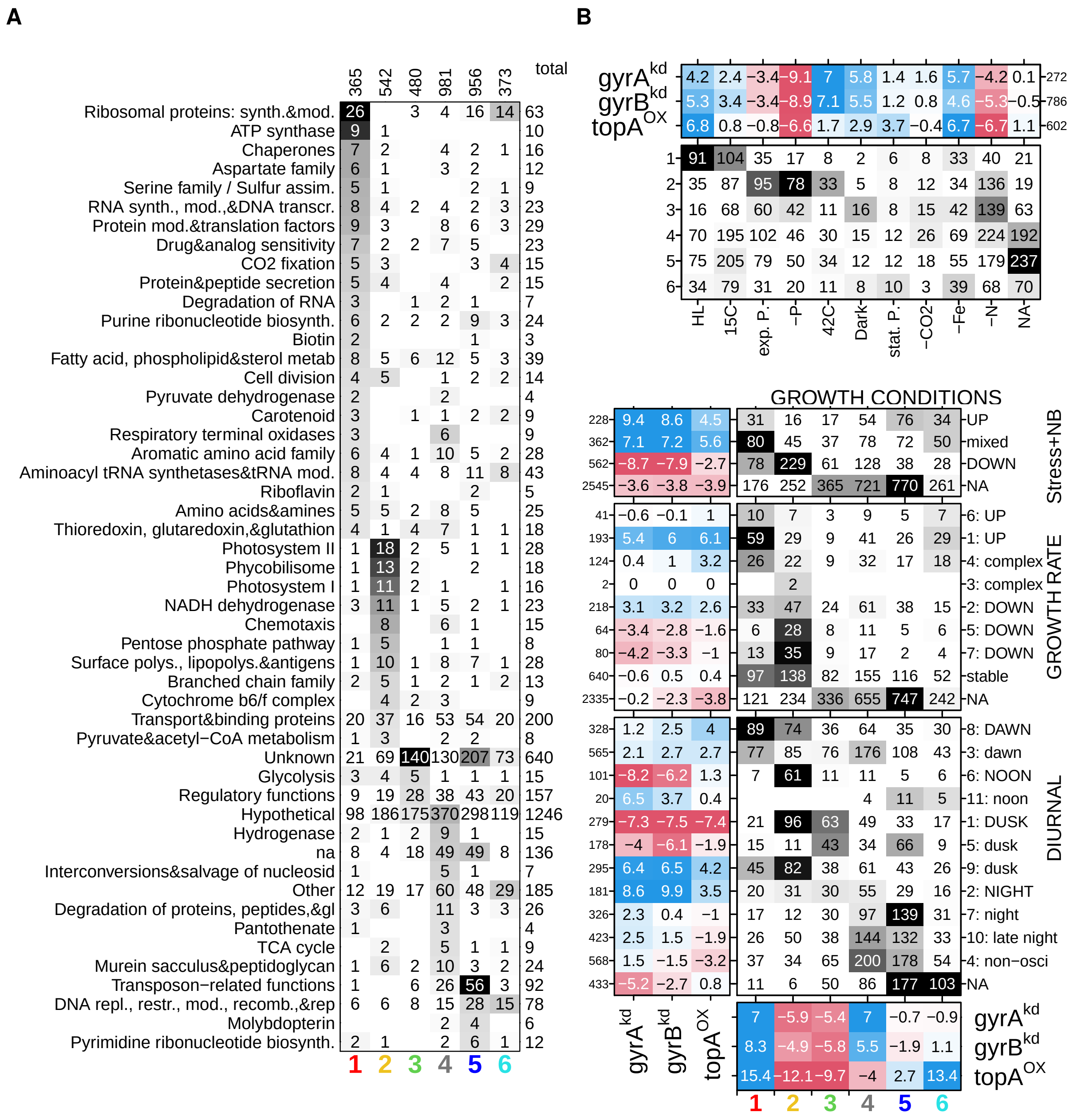

Figure S14. `CyanoBase` **Category Analysis of Co-Expressed Cohorts.** **A:** Sorted enrichment profile of functional category annotations as in Figure 6B (colored with $p_{\text{min}} = 10^{-10}$ and $p_{\text{text}} = 10^{-5}$) but sorted at $p_{\text{sort}} = 0.1$. All categories below the red line had only $p > p_{\text{sort}}$ and are unsorted. Some abbreviations of the original annotation terms are used for readability of the plot: synth. - synthesis, mod. - modification, repl. - replication, transcr. - transcription, recomb. - recombination, restr. - restriction, s. - saccharides, assim. - assimilation, & - and. **B:** Overlap enrichment and t-test profiles with clusterings as Figure 6C but for additional gene classifications from other publications; from top to bottom: experimental `GROWTH CONDITIONS` with maximal expression of transcription units from Kopf *et al.* [12], stress and novobiocin (`Stress + NB`) treatment (same as in Fig. 6C) by Prakash *et al.* [13], the original non-collapsed clustering of protein abundance level response to `GROWTH RATE` by Zavrel *et al.* [14], and a clustering of a `DIURNAL` transcriptome data set from the supplemental material by Lehmann *et al.* [15]. Numbers in the t-test fields (red and blue color scales) are the t statistic.

	365	542	480	981	956	373	total
structural constituent of ribosome	25		2	2	14	13	56
translation	25		2	6	15	14	62
rRNA binding	17		1	2	8	8	36
proton−transporting ATP synthase activity, rotational mechanism	8	1					9
cytosolic small ribosomal subunit	10		1	1	2	1	15
cytoplasm	55	33	26	82	67	27	290
ATP binding	53	43	28	61	71	36	292
plasma membrane proton−transporting ATP synthase complex	6	1					7
cytosolic large ribosomal subunit	10				4	6	20
ATP synthesis coupled proton transport	5						5
proton−transporting ATP synthase complex, coupling factor F(o)	5						5
cytosol	41	31	18	59	46	24	219
chaperone cofactor−dependent protein refolding	5			1			6
GTP binding	12	5	2	5	9	2	35
tRNA binding	10	1	1	3	4	7	26
RNA binding	12		2	12	9	2	37
unfolded protein binding	7	3	1	3	1		15
proton−transporting ATP synthase complex, catalytic core F(1)	4	1					5
DNA−directed 5'−3' RNA polymerase activity	4				1		5
transcription, DNA−templated	4				1		5
protein transport by the Sec complex	4					1	5
protein targeting	4					1	5
mRNA binding	5	1		1	2		9
protein folding	5	2	1	1	2		11
threonine biosynthetic process	4	1		1		1	7
GTPase activity	7	5	3	3	3		21
response to heat	3				1		4
carotenoid biosynthetic process	3				1		4
carboxysome	5	1			2	5	13
metal ion binding	33	43	21	59	37	25	218
protein transport	4		1	2	1	1	9
rRNA processing	4			3	2		9
RNA processing	3			2			5
ribosomal small subunit assembly	3				2		5
intracellular protein transmembrane transport	3	1				1	5
translation elongation factor activity	3	1				1	5
thylakoid membrane	17	47	12	11	2	5	94
glycogen debranching enzyme activity	2						2
cytochrome complex	2						2
cell envelope Sec protein transport complex	2						2
cytoplasmic translation	2						2
GroEL−GroES complex	2						2
NAD metabolic process	2						2
NADP biosynthetic process	2						2
translational elongation	2						2
'de novo' CTP biosynthetic process	2						2
CTP biosynthetic process	2						2
photosynthesis	7	41	11	14	3	6	82
plasma membrane−derived thylakoid photosystem II	1	19	2	4	2		28
integral component of membrane	60	149	64	216	130	49	668
inorganic phosphate transmembrane transporter activity		7					7
phycobilisome	3	13	1	2	2	3	24
quinone binding	3	12	2	4			21
protein−chromophore linkage	1	15	4	5	3	3	31
photosystem II reaction center		7		1			8
photosynthesis, light reaction		8	1	1	1		11
NADH dehydrogenase (ubiquinone) activity		9	1	4		1	15
chlorophyll binding		7	1	1		1	10
electron transporter, transferring electrons within the cyclic electron transport pathway of photosynthesis activity		6	1	1			8
plasma membrane−derived photosystem I		5				1	6
phosphate ion binding		4					4
phosphate ion transmembrane transport		4					4
iron ion binding	3	13	4	11	4		35
photosynthetic electron transport in photosystem II		5	1	1			7
photosystem I	1	5	1				7
metalloendopeptidase activity	4	10		7	1	3	25
efflux transmembrane transporter activity		4		1			5
photosynthetic electron transport chain		4				1	5
nitrate assimilation		4			1		5
heme binding	3	9	6	2	2		22
photosystem II stabilization		3					3
ATPase−coupled phosphate ion transmembrane transporter activity		3					3
oxidoreductase activity, acting on iron−sulfur proteins as donors		3					3
oxidoreductase activity, acting on the CH−CH group of donors, iron−sulfur protein as acceptor		3					3
photosynthesis, dark reaction		3					3
oxidation−reduction process	1	11	3	9	5	2	31
NADP binding	3	7	1	1	3	1	16
outer membrane−bounded periplasmic space	7	19	12	17	9	7	71
light−independent chlorophyll biosynthetic process		4		2			6
photosystem II		4	1	1			6
transmembrane transporter activity	3	11		7	9	3	33
electron transfer activity	5	12	3	12	4	3	39
NOT ANNOTATED	50	126	225	311	386	134	1232
histidine phosphotransfer kinase activity		1	8	3	3	3	18
protein histidine kinase activity		2	8	3	3	3	19
monosaccharide binding			3				3
protein autophosphorylation		2	8	4	3	3	20
protein disulfide oxidoreductase activity	1		4	1	1		7
protein−disulfide reductase activity	1		3				4
oxidoreductase activity	8	8	4	28	13	5	66
chemotaxis	1	1		7	1		10
transposase activity				4	22		26
transposition, DNA−mediated				4	22		26
DNA binding	13	10	13	34	61	20	151
DNA−directed DNA polymerase activity	1				7		8
endonuclease activity	1	2	1		10	2	16
defense response to virus	1		3		9	2	15
maintenance of CRISPR repeat elements					5	1	6
exodeoxyribonuclease V complex						3	3
single−stranded DNA helicase activity						3	3
ribosomal large subunit assembly	2					4	6
siderophore uptake transmembrane transporter activity					1	3	4
type I site−specific deoxyribonuclease activity			1			3	4
DNA modification			1		1	3	5
	1	2	3	4	5	6	

Figure S15. GO Analysis of Co-Expressed Cohorts. Sorted enrichment profile as in Figures 6B and S14A (colored with $p_{\text{min}} = 10^{-10}$ and $p_{\text{text}} = 10^{-5}$) but for Gene Ontology (GO) terms, downloaded from the `UniProt` database (2021-03-20, `organism:1111708`). Rows are cut and sorted along columns at $p_{\text{sort}} = 0.01$.

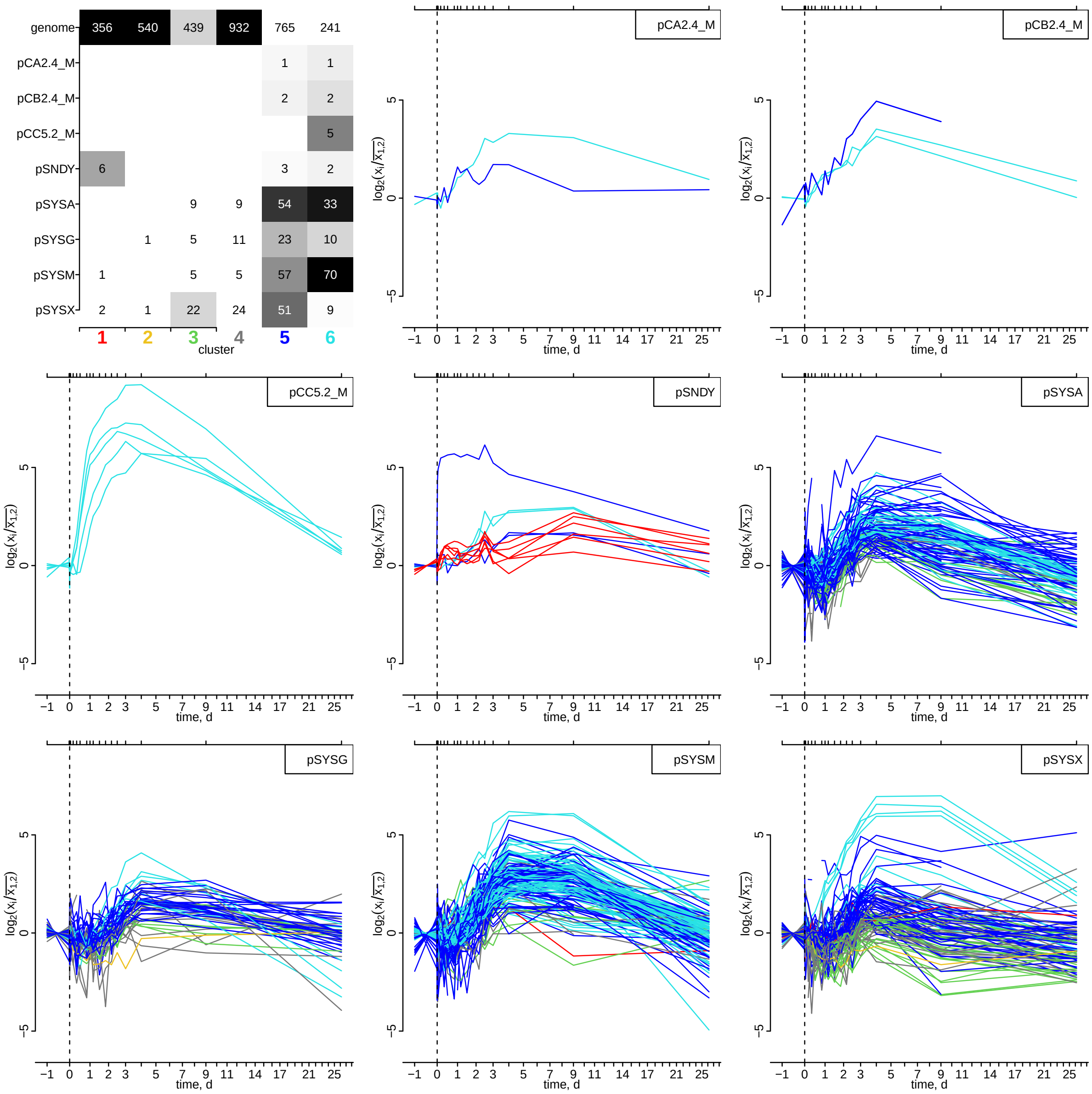

Figure S16. Transcriptome Time Series - Plasmids. Top left panel: Enrichment profile of time series clusters with the locations on the chromosome, one of the seven endogenous plasmids, or our construct pSNDY [2] (Table S1). All other panels show the temporal transcript abundance profiles for the coding genes of each plasmid (see top right legends for plasmid names); each transcript is colored according to its cluster label. Missing values stem from 0 read-counts in the raw data.

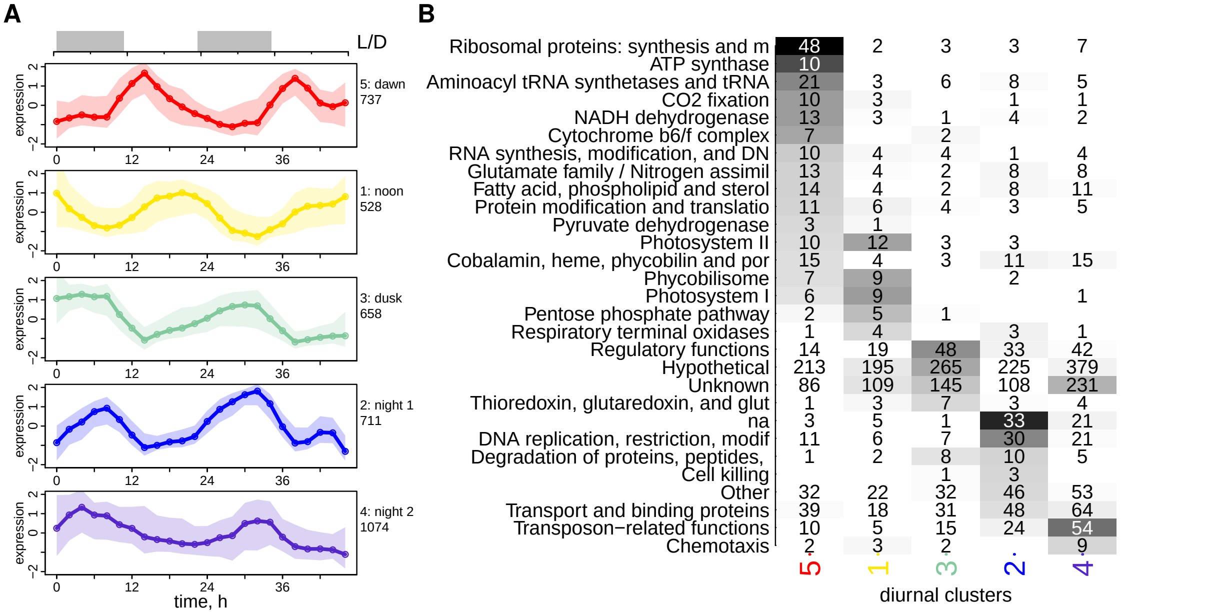

Figure S17. Diurnal Co-Expression Cohorts. Clustering of diurnal transcriptome data by Saha *et al.* [16] into 5 co-expression cohorts, using the Fourier transformed data and the `flowClust` algorithm. The maximal BIC clustering at $K = 5$ clusters was used for analysis. **A:** Cluster medians of the normalized (to mean 0) expression values with an additional moving average over 3 samples. Transparent ranges show the 10% and 90% quantiles, *i.e.* they encompass 80% of all values in a cluster. Cluster labels and sizes (number of genes) are indicated on the right y-axis. The gray and white bars on the top indicate dark and light phases of the experiment. **B:** Enrichment profiles of co-expressed cohorts with `CyanoBase` functional categories as for Figure 6B (colored with $p_{\text{min}} = 10^{-10}$ and $p_{\text{text}} = 10^{-5}$), but cut and sorted at $p_{\text{sort}} = 0.05$.

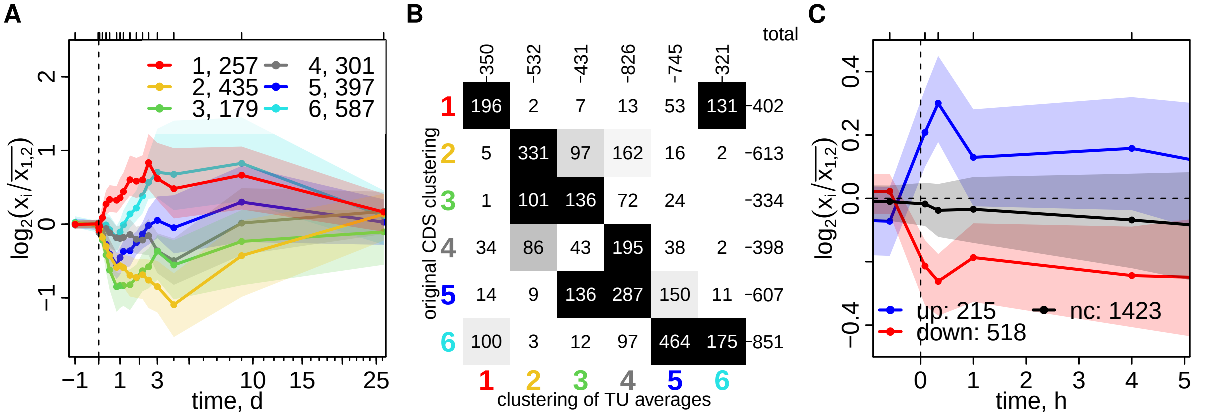

Figure S18. Transcription Unit Clustering. A: Clustering of transcription units (TU) defined by [12]. Average expression was calculated for all TU from the expression of coding genes they encompass (*via* the "Sense.tags" column of the original data set), and the resulting TU time-series was clustered by k-means, using cluster centers from the CDS clustering (Fig. 6, S12) and identical time-series processing. The number of TU in each cluster is indicated in the legend. The lines and dots are the medians and the ranges show the $25\,\%$ and $75\,\%$ quantiles of each cluster. **B:** enrichment profile of the original CDS clustering (y-axis) with the TU-based re-clustering; colored with $p_{\text{min}} = 10^{-10}$ and $p_{\text{text}} = 10^{-5}$ and with the original order. **C:** As (A) but for immediate response clusters. All TU were classified by the log2 ratio of the means of the two post-induction time points to the means the two pre-induction time points ($\log_2\left(\overline{x_{3,4}}/\overline{x_{1,2}}\right)$); up: > 0.15, down: < -0.15, and nc: all others. The number of TU in each class is indicated in the legend.

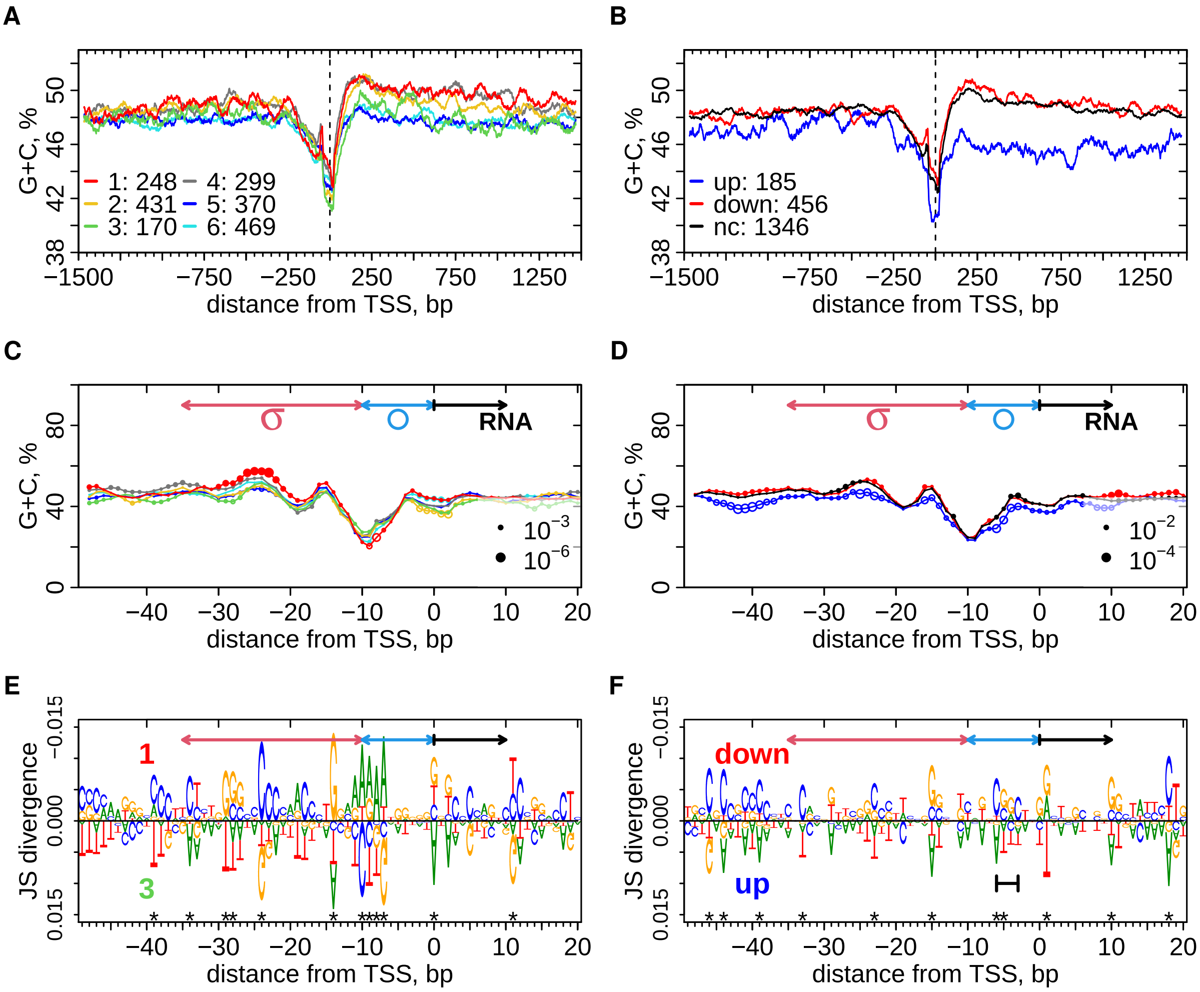

Figure S19. Long Range G+C Content and the Discriminator Region. The G+C frequencies of clustered TU (Fig. S18) aligned at their transcription start sites (TSS) were calculated in 66 bp bins (A and B) or 5 bp bins (C and D) around the TSS, sequence logos (E and F) were calculated without binning. **A & C:** Nucleotide frequency profiles for the differential response clusters shown in Figure S18A. (A) is identical to Figure 7A, and reproduced here for comparison. **B & D:** same as (A and C) but for the immediate response clusters (20 min post-induction) described in Figure S18C. The cluster legends in (A) and (B) provide the number of TUs in each cluster. Note, that only TU from the main genome were considered for this analysis, therefore the numbers are lower than those reported in Figure S18. The point sizes in (B) and (D) scale with $-\log_2(p)$, where p are the minimal p-values of two-sided cumulative hypergeometric distribution tests of the total counts in each cluster. Filled points indicate significant enrichment and open circles indicate significant deprivation of the motif count (here simply G or C on the forward strand) in the respective cluster. The dot size for the minimal and half-minimal p-values are indicated in the bottom-right legends. **E & F:** The Jensen-Shannon (JS) divergence [17] between the position weight matrices of the indicated promoter clusters; as in Figure 7E-F, reproduced here for direct visual comparison with G+C frequency profiles (B-D). The short horizontal bar in (F) indicates the GC discriminator region -6 to -3. See S24-S25 for details.

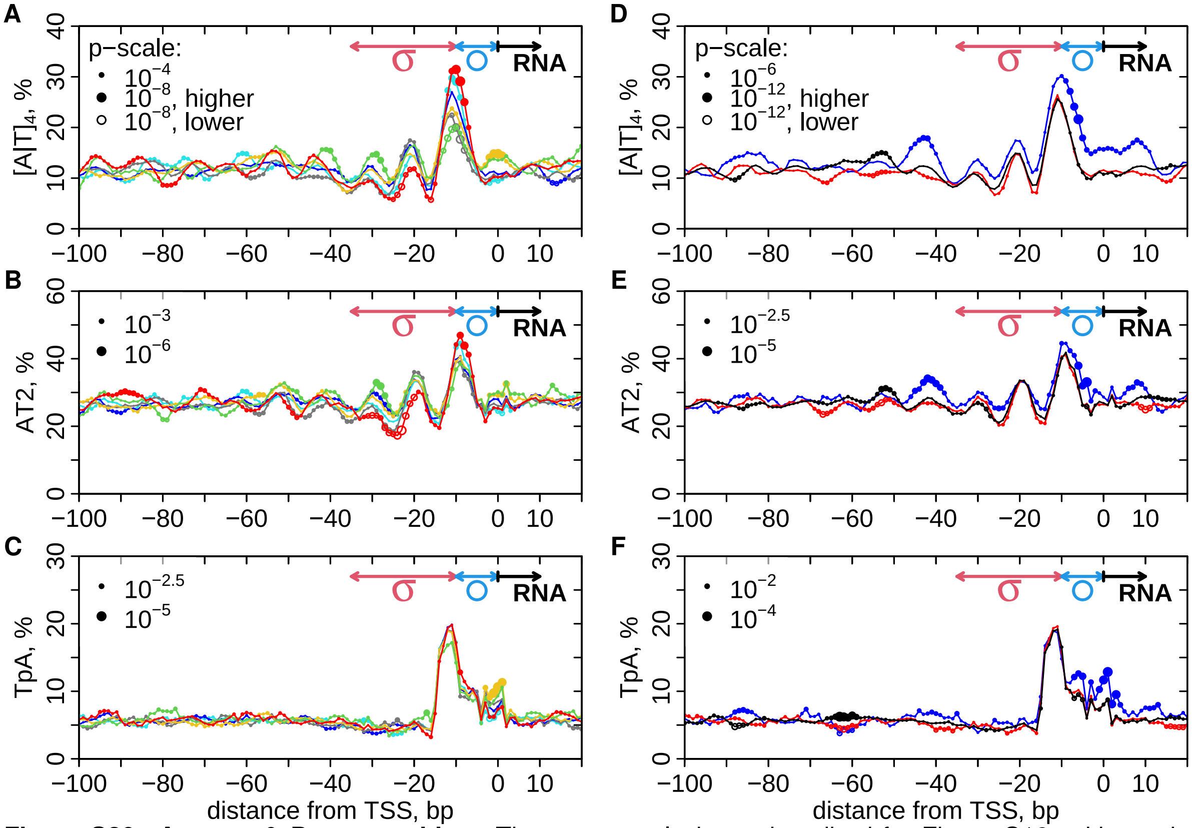

Figure S20. A-tracts & Decomposition. The same analysis as described for Figure S19, with moving averages over 5 bp, and for different DNA motifs: "[A|T]$_4$" are repeats of A and T nucleotides of length 4 (A-tracts). The "AT2" motif are dinucleotides ApA, ApT or TpA, and "TpA" is the dinucleotide "TpA". **A–C** are frequency profiles for the differential response TU clusters (Fig. S18A). **D–F** are frequency profiles for the immediate response clusters (Fig. S18C).

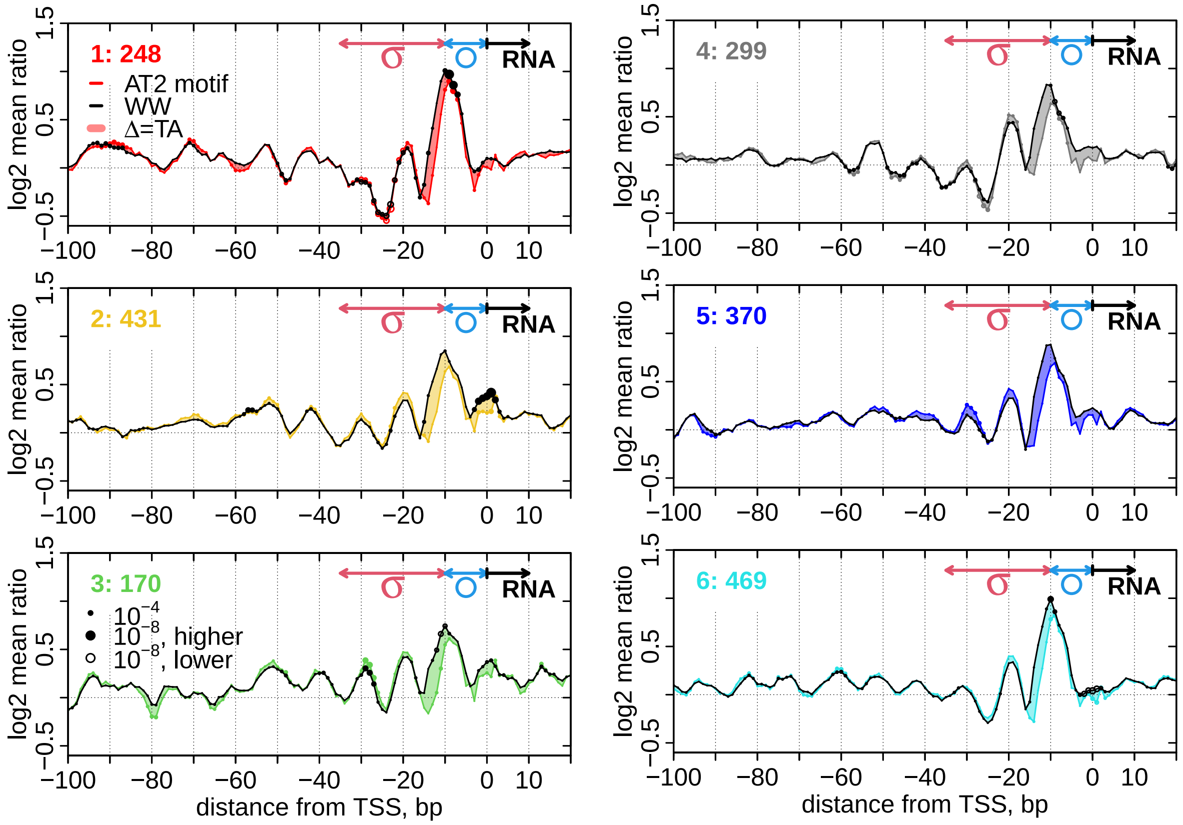

Figure S21. Normalized WW and AT2 Motif Frequencies. Direct visualization of the spatial relation of periodic enrichment of the AT2 motif and localized enrichment of the TpA step. The log2 mean ratios of the WW (black) and the AT2 dinucleotide motifs (colored by cluster) were calculated as described for Figure 7B and then normalized by dividing each position by the frequency in the total analyzed window. The TpA step is the difference between the WW and the AT2 motif frequencies and indicated by shade.

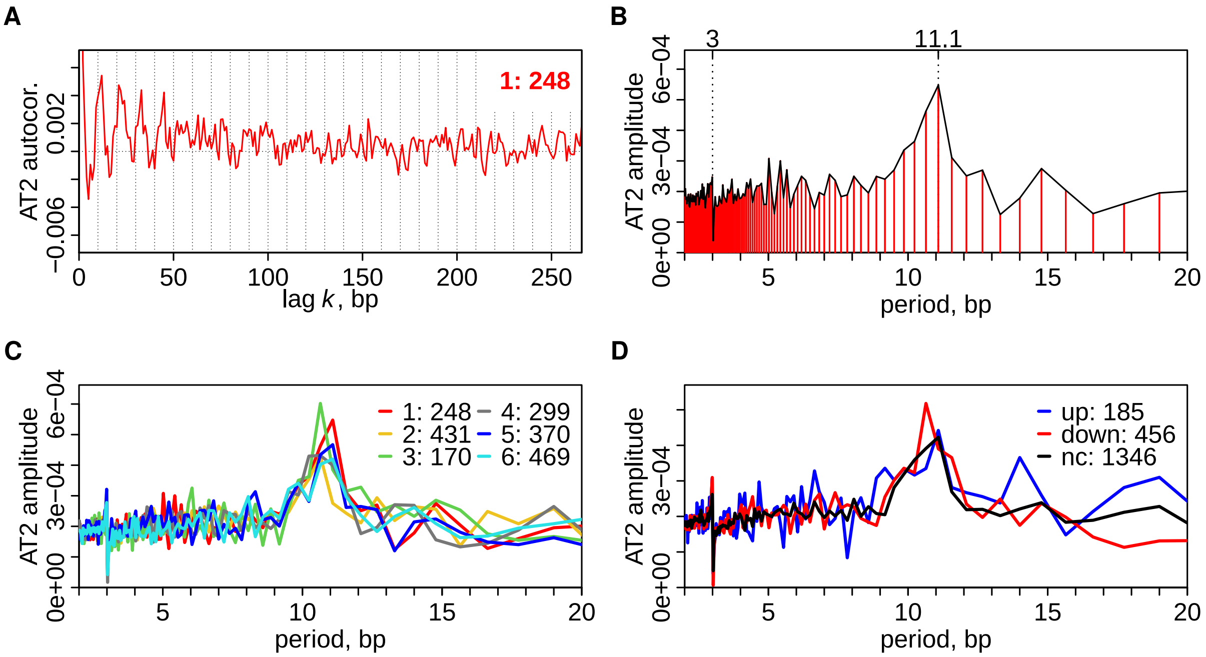

Figure S22. Autocorrelation Analysis of AT2 Motif Frequencies in Upstream Regions. A: The autocorrelation function of AT2 motif frequencies in concatenated promoter sequences (-150 bp–$+15$ bp around the TSS), here exemplary for cluster 1, was calculated after Schieg and Herzel [18] and as described by Lehmann, Machné and Herzel [15], but without removal of the first 30 positions which controls for periodic coding regions by alpha helices. **B:** Power spectrum of the autocorrelation functions in (A). **C & D:** Power spectra of the AT2 autocorrelation function is described in (A and B) but for all differential response clusters (C, Fig. S18A) and for all immediate response clusters (D, Fig. S18C).

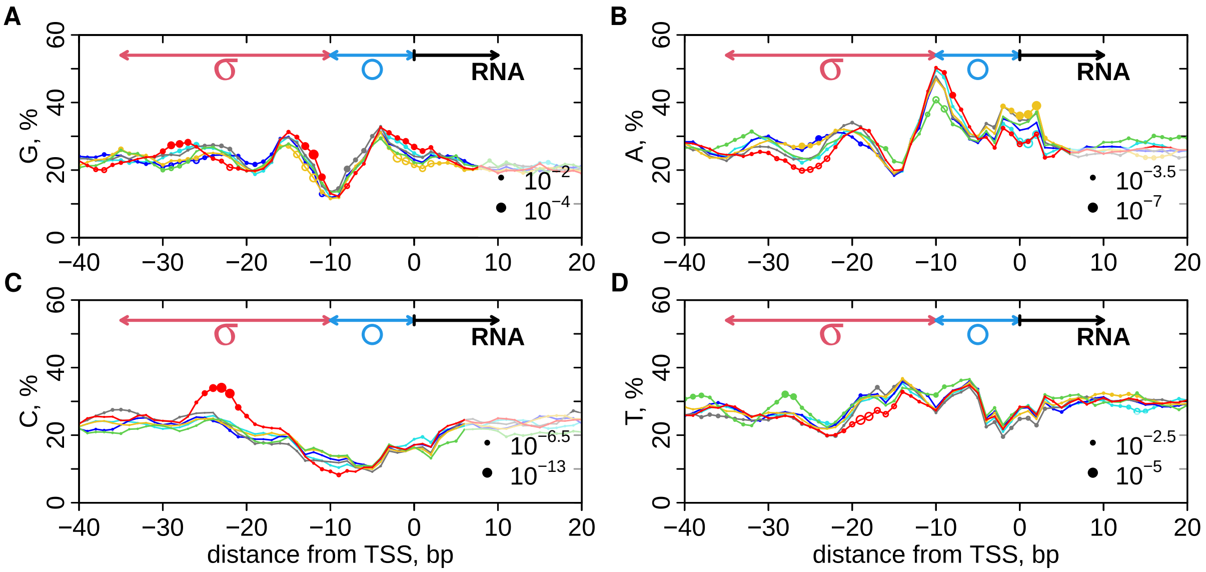

Figure S23. Single Nucleotide Frequencies. The same analysis as described for Figure S19, with moving averages over 5 bp, and for single nucleotides.

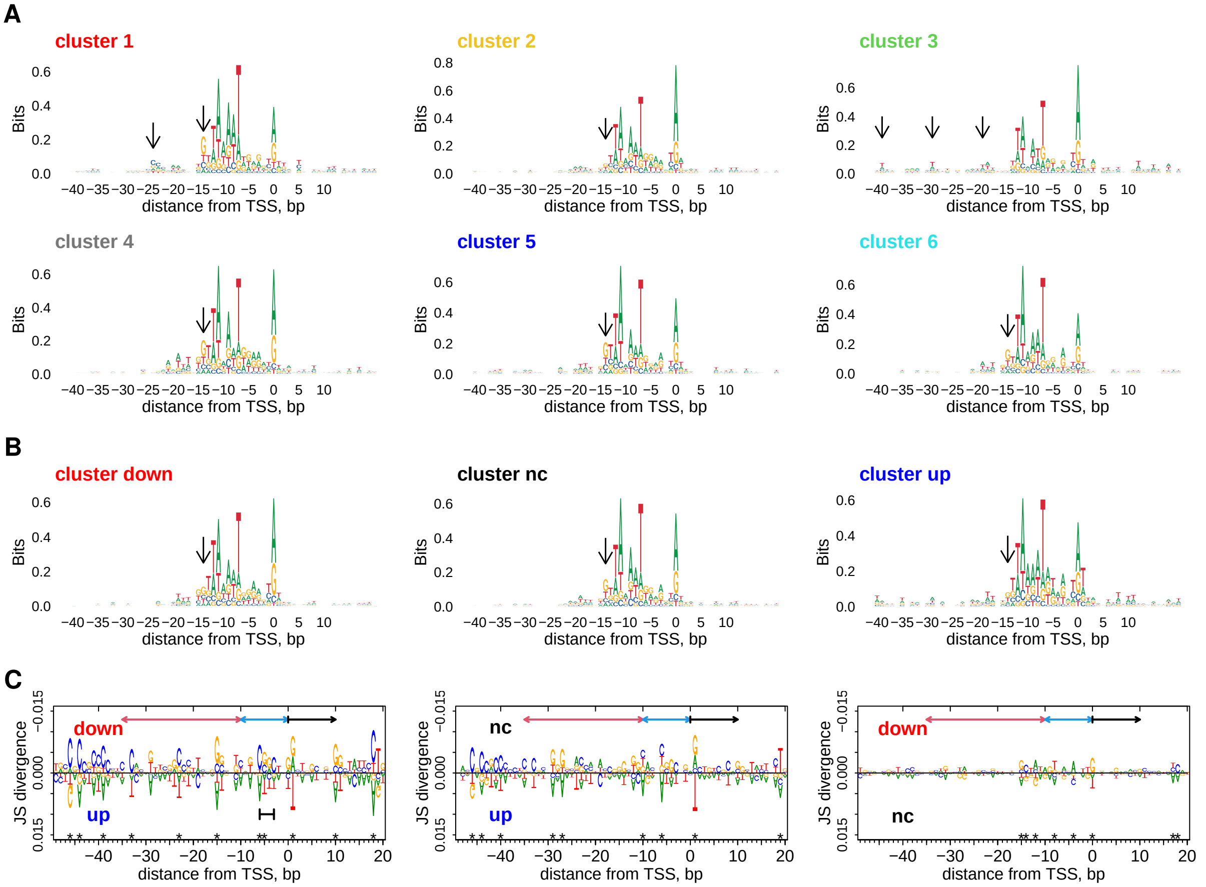

Figure S24. Sequence Logos at the Transcription Start Site. Sequence logos [19] were generated with `ggseqlogo` (`https://CRAN.R-project.org/package=ggseqlogo`) for the time series clusters 1-6 (**A**) and the early response clusters (**B**) from Figures S18A and C. The Jensen-Shannon (JS) divergence (**C**) between the early response clusters in (B) was calculated with `DiffLogo`, where $*$ indicate significance at $p < 0.05$ [17]. In all plots the TSS is 0, thus +1 should be added to get conventional nomenclature, where TSS is +1. **A-B:** All clusters showed overall similar sequence properties, reflecting previous results [20–24]: an enrichment of T and A at -12 and -11, and T at -7. The former (-12/-11) mark the first nucleotides of the open DNA bubble during initiation of transcription. The latter (-7) binds to a specific pocket in the σ^{70} factor of *E. coli* during open bubble formation [25–27]. A G at -14 ("extended -10") is enriched in all clusters except the downregulated clusters 2 (yellow) and 3 (green). This was also observed in promoters of genes upregulated in the ΔsigBCDE strain [23], and was stronger in SigA-bound than in SigE-bound TU [24]. Only cluster 1 (red) had a weak enrichment of C at -22 to -24, and only cluster 3 (green) showed weak enrichments of T at ca. -29, -39 and -49, i.e., in helically phased distances. A T at ca. -30 was also observed in genes downregulated in a ΔrpoZ strain [22]. **C:** The difference logo confirms a lower GC-content in the discriminator region, (marked by horizontal bars from -6 to -3), in the promoters that were upregulated ("up") immediately after topA^{OX} induction than in downregulated promoters ("down"), but shows similar enrichments throughout the core promoter.

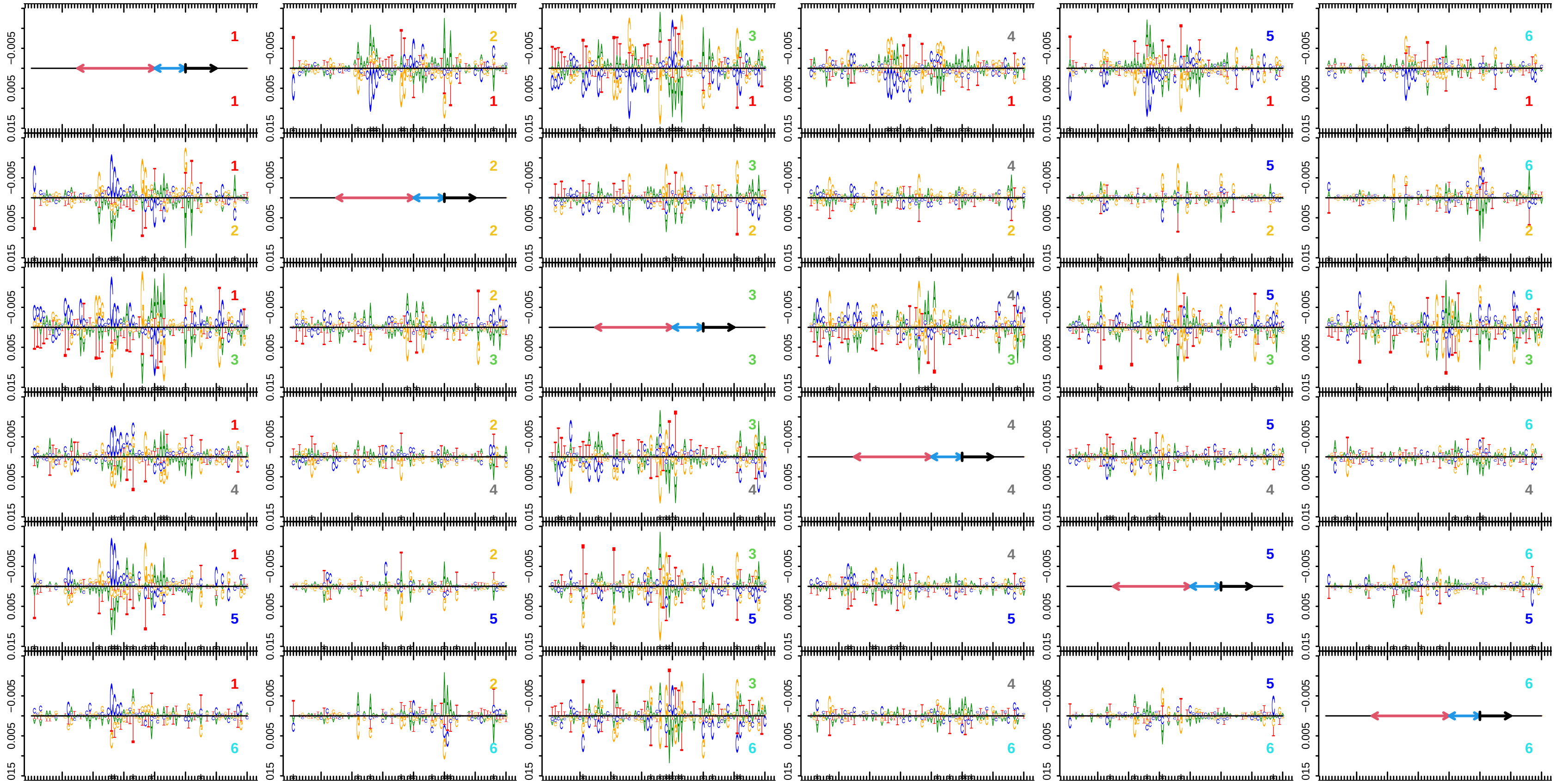

Figure S25. Difference logos for all pairs of time series clusters, see Figure S24A for details. The arrows indicate the -35, -10 and TSS (0) positions, as in all other promoter plots. The difference logos confirm the enrichment of C between -20 and -25 and A between -10 and -7 of cluster 1 promoters (Fig. S23), and the helically phased enrichment of A-tracts in cluster 3 (Fig. S20A).

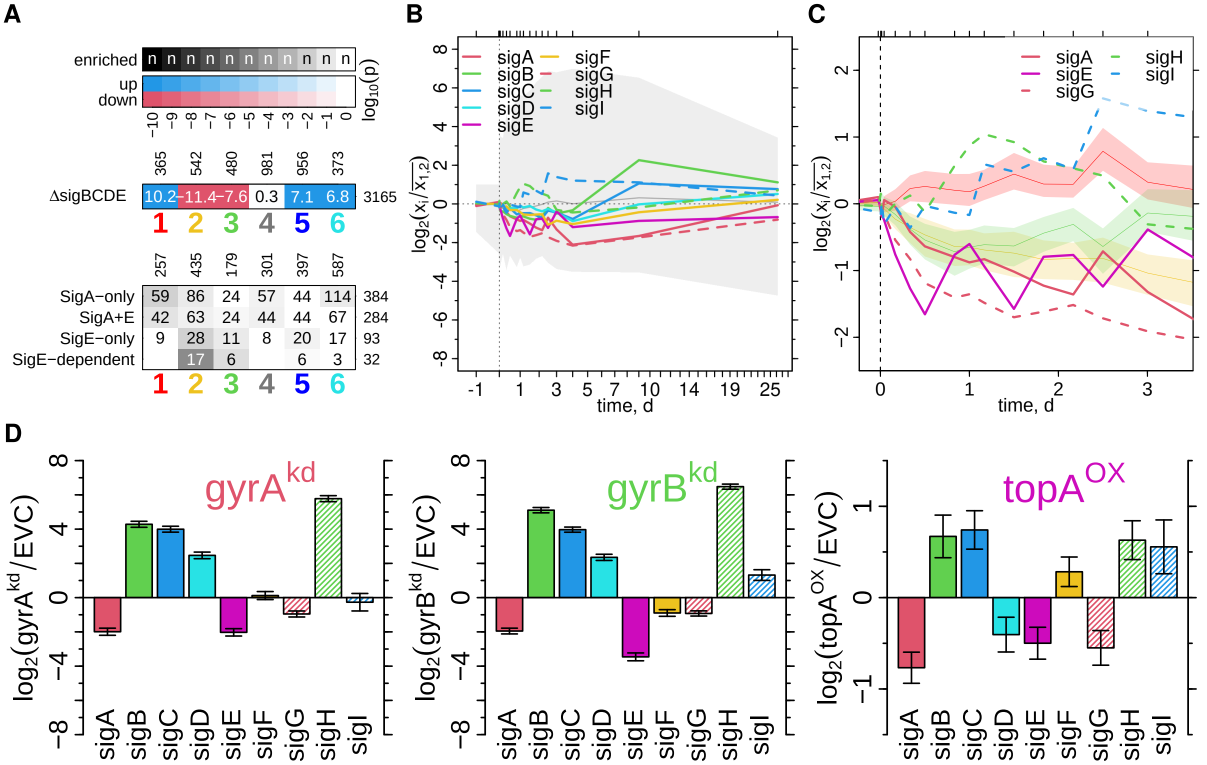

Figure S26. Sigma Factor Expression and Target Genes. *Synechocystis* carries 9 different sigma factors. SigA is essential and activates the transcription and translation machineries during autotrophic growth conditions [28]. The target genes of group 2 sigma factors (SigBCDE) overlap with SigA target genes and are involved in transcriptional remodeling during dark/light transitions and rapid respond to various adverse conditions [23, 24, 29, 30]. SigD and SigE may be involved in circadian control of transcription, since their transcript and protein abundances showed diurnal patterns [30–32]; and SigE activates transcription of sugar catabolic pathways, incl. glycogen breakdown [33]. The functions of group 3 sigma factors (SigFGHI) are not well studied. They lack the domain 1.2 [28] which in *E. coli* is known to mediate differential sensitivity of promoters to ppGpp [34]. SigH is upregulated and SigG downregulated during heat stress [35], and SigH is involved in acid acclimation [36]. SigI is upregulated during the dark phase of the diurnal cycle [16, 37]. **A:** T-test profile of cluster expression (by single genes, as in Fig. 6A) in the ΔsigBCDE strain [23] (blue/red color scale) and cluster enrichment profile (black/white color scale) of the TU clusters (Fig. S18) with SigA-bound, SigE-bound and SigE-dependent TU (column "TSSs within 100bp of peak summit" of Table S6, and Table 1 of [24]). Total numbers in each group are shown on the top and right axes and colors scale with the p-values as indicated in the legend. **B & C:** Time-series of normalized transcript abundances (Fig. 6, S12) for all nine annotated sigma factors of *Synechocystis* [28], for the full time series (B) and as a zoom on the first 3 days post-induction for selected sigma factors and with 25%/75% quantile ranges of clusters 1–3 shown as transparent ranges (C). **D:** Normalized abundances of the sigma factor transcripts in the endpoint measurements of the gyrA^{kd}, gyrB^{kd} and topA^{OX} strains (Fig. 4). **Summary:** SigA-bound TU are slightly enriched in the upregulated cluster 1 (A, bottom), yet the *sigA* transcript is downregulated in all conditions (B-D). Transcripts of the group 2 factors *sigB* and *sigC* are upregulated in all conditions (D), but only as a late adaptive response in the topA^{OX} time series (B); *sigD* is upregulated only in the gyr^{kd} strains, and *sigE* is strongly downregulated in all conditions (D). The time series (B-C) reveals a notable circadian pattern in *sigE* downregulation, preceding the cluster 3 peaks, while *sigH* is transiently upregulated during day 1, accompanied by an upregulation of *sigI* in three circadian steps, preceding the circadian peaks of cluster 1 transcripts. Thus, the response of sigma factors in our experiments may be related to both the glycogen-enriched phenotype (*sigE*, [33]) and the circadian pattern of gene expression after topA^{OX} induction. However, more data, e.g., chromatin-immunoprecipitation based binding studies, are required to test the relevance of these pattern.

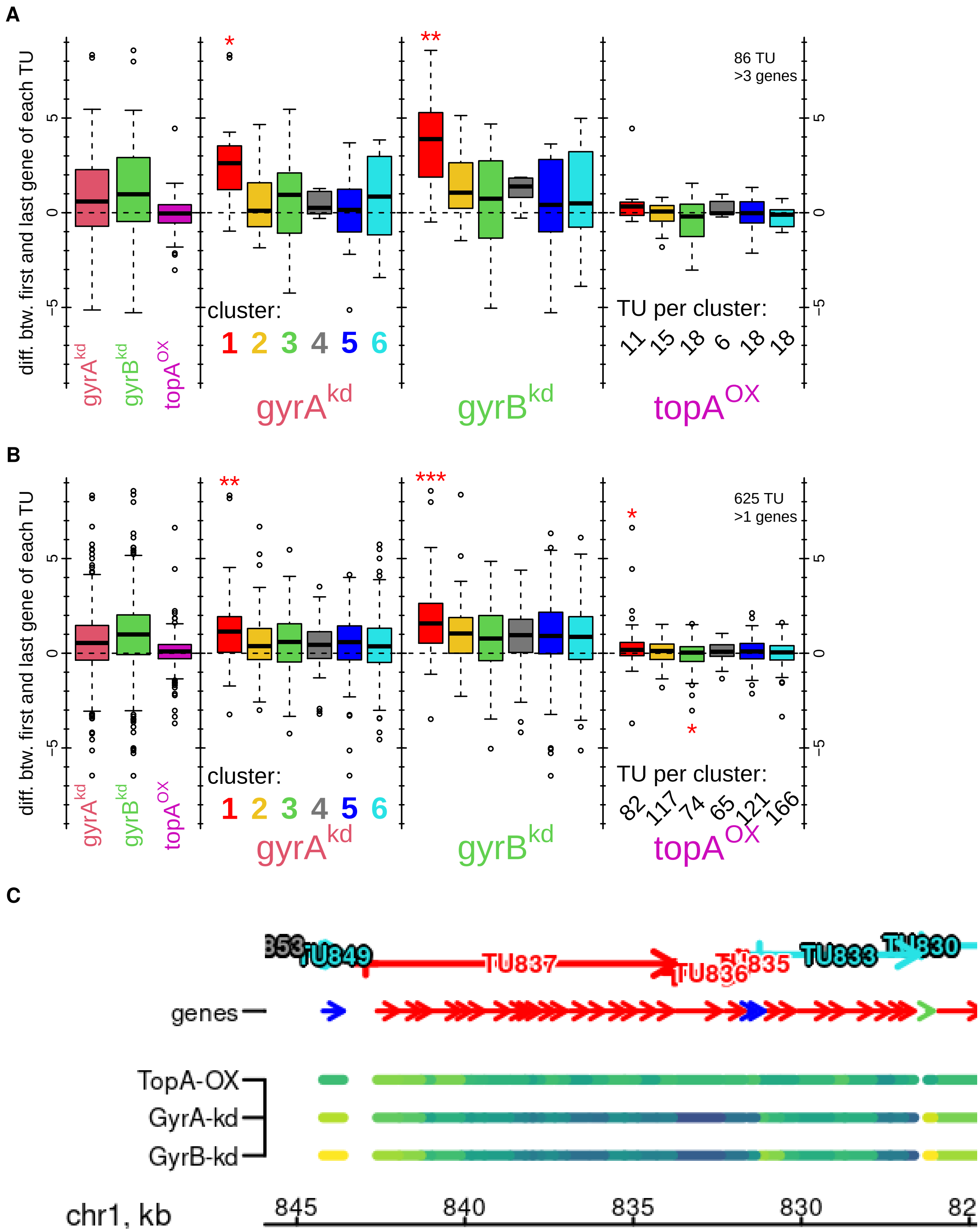

Figure S27. Graded Response Along Transcription Units. A & B: As Figure 7G but for all strains and all TU with ≥ 4 genes (A) or for all TU with ≥ 2 genes (B). Red stars above or below the boxplots indicate significance (*: $p < 0.05$, **: $p < 0.01$ and ***: $p < 0.001$) in two-sided t-tests of each cluster vs. all other clusters. **C:** Example TUs coding for ribosomal proteins. TU and coding genes are colored by their cluster labels. The transcript abundance levels of coding genes relative to the empty vector control in the endpoint experiments are color-coded by the `viridis` color scheme such that blue are lower and yellow are higher values. The values become progressively lower along the TU, in 5' to 3' direction, the gyrA^{kd} and gyrB^{kd} strains but not in the topA^{OX} strain.

Appendix A: Reactor Dynamics

Transient Increase in Cell Volume and Density. To study the dynamic response to transient *topA* induction, the topA^{OX} strain was grown in a Lambda Minifor bioreactor (Fig. A1) with continuous (online) monitoring of turbidity (OD$_\lambda$, Fig . A2A,B). Continuous culture dilution was initiated at OD$_\lambda$ ≈ 2.9 and with dilution rate $\phi \approx 0.24\,\mathrm{d}^{-1}$. The culture stabilized around OD$_\lambda$ ≈ 2.7. Notably, a subtle ≈ 24 h pattern of OD$_\lambda$ was observed in both batch and pre-induction continuous growth phases. Then rhamnose was injected to 2 mM to induce overexpression of *topA*. The *topA* transcript was upregulated to ≈45-fold over the pre-induction level within 4 h, as measured by RT-qPCR and confirmed by RNA-seq (Fig. S10) and decreased slowly over the course of the experiment. The OD$_\lambda$ initially increased for 1 d post-induction, then slowly decreased. Cell dry weight (CDW) measurements were noisy but matched the OD$_\lambda$ signal over the sampled period (Fig. 5A, A2C). In contrast, cell numbers started to decrease immediately, and cell volumes increased (Fig. S11A). We calculated growth rates of OD$_\lambda$, cell numbers and the total cell volume (Fig. S11C, A3). Cell division was not completely blocked but severely reduced to a division time of ≈10 d ($\mu_{\text{count}} \approx 0.07\,\mathrm{d}^{-1}$). Total cell volume growth was much less affected and remained stable ($\mu_{\text{volume}} \approx 0.18\,\mathrm{d}^{-1}$) throughout continuous culture operation until 12 d post-induction. Thus, artificial *topA* overexpression blocked cell division but not cell volume growth. OD$_\lambda$ growth remained highest ($\mu_{\text{OD}} \approx 0.23\,\mathrm{d}^{-1}$) and stable over the first 5 d–6 d. In parallel, glycogen content increased to about 35 %–40 % of the CDW (Fig. 5A). We further noticed that sampled cells started to sediment much faster, indicating increased intracellular density. By calibrating the OD$_\lambda$ signal to the CDW measurements (Fig. A2C) and dividing by the total cell volume we can estimate a CDW density and this value also increased over time from 0.3 to 0.5 $\mathrm{g}_{\text{DCW}}/\mathrm{mL}_{\text{cell}}$ (Fig. S11B). This range is consistent with data from *E. coli* [38, 39]. However, the CDW per OD$_{750}$ was relatively lower for the enlarged strains in the endpoint measurement (Fig. 2A), and thus, the calibration to OD$_\lambda$ may overestimate true CDW density. The enlarged and denser cells also became increasingly fragile: in the CASY cell counter data a small population of varying intensity appeared at <2 fL. This peak was highest at 7 d (outlier $\mathtt{x}$ in Fig. S11A), where cells were lysed during centrifugation in a washing step. The washing step was skipped thereafter, and the peak of small cells (dead or fragmented) remained small but increased towards the end of the continuous culture. Maximal cell volumes >20 fL were reached 10 d–15 d post-induction. From day 14 a population of smaller cells, ≈ 7.5 fL, appeared. On 16 d this population was the majority, and cell volume further decreased to 5 fL. Cell pigmentation recovered and the culture appeared greener again. We then switched off dilution, and the culture resumed growth, although at lower growth rates than pre-induction.

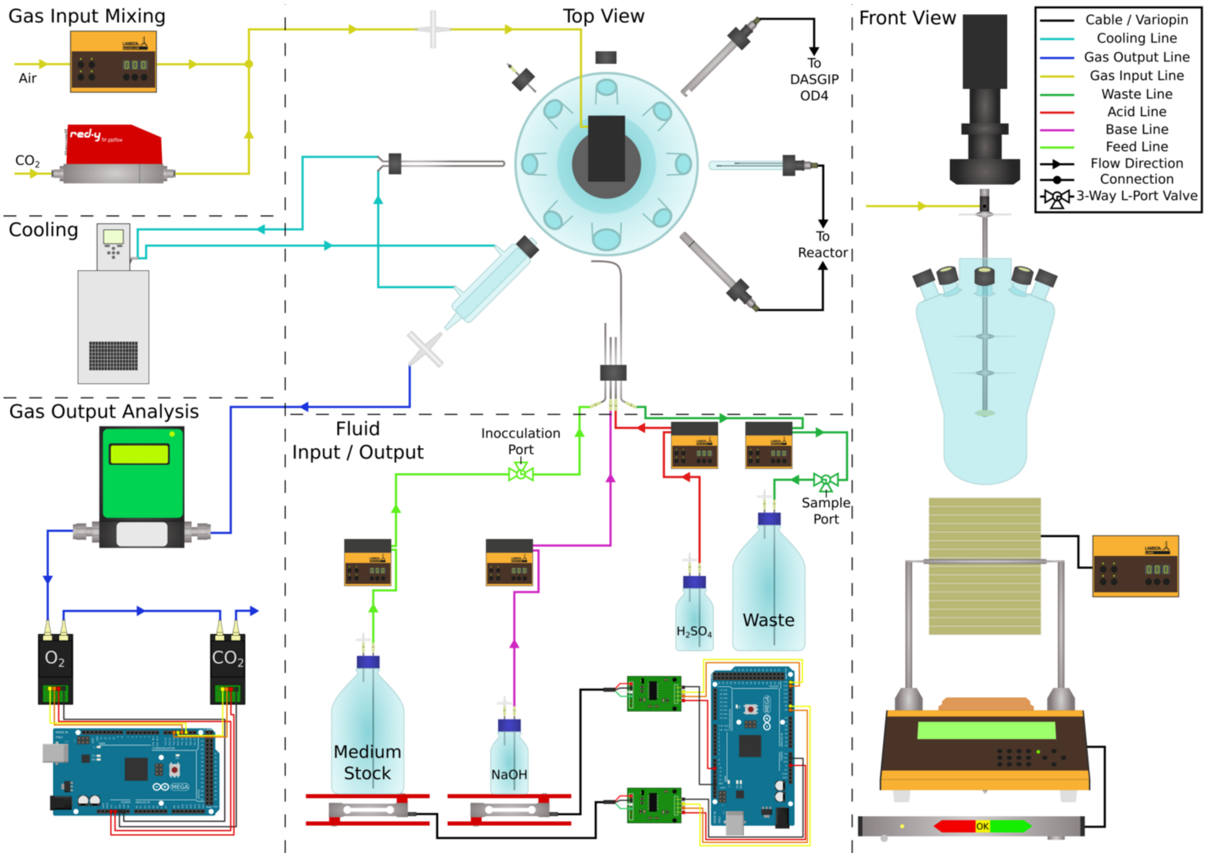

Figure A1. Photobioreactor Setup. A schematic overview of the cultivation setup showing the Lambda Minifor bioreactor in front and top-down views alongside its external components and custom expansions. The gas input mixture is generated by a Lambda MASSFLOW 5000 gas flow controller and a Voegtlin red-y smart controller, which regulate the flow of compressed air and CO_2 respectively. This input gas mixture is then introduced into the cultivation vessel via the sparger at the end of the agitation unit. The offgas condenser as well as the reactor's cooling finger are part of a water cooling circuit which is regulated by a Lauda Eco Silver thermostat set to $16\,°C$. An Aalborg Massflow Meter monitors the flow rate of the culture's offgas before it is lead through a custom microcontroller-based gas sensor array in order to evaluate its O_2 and CO_2 content. The reactor actively regulates the culture's pH and temperature values by controlling its heating compartment as well as the Lambda Preciflow peristaltic pumps which are attached to NaOH and H_2SO_4 stock bottles, each $0.5\,M$. Additional culture parameters are monitored by a dissolved O_2 probe attached to the reactor and an OD4 probe connected to a DASGIP OD4 device. An additional set of peristaltic pumps is attached to the culture's medium stock and waste containers in order to control the reactor's volume and medium turnover. The reactor weighting module enables the system to operate under chemostat conditions. This is achieved by manually configuring the medium feed peristaltic pump at a constant speed in order to achieve a desired medium turnover rate while automatically regulating the waste pump speed to keep the total reactor weight constant. Additionally, a custom microcontroller-based scale setup is monitoring the weight of both the medium and NaOH stock bottles, which allows for the calculation of medium and base pump rates from the recorded data. The culture's illumination is provided by the Lambda LUMO modules, an LED strip fitted around the cultivation vessel.

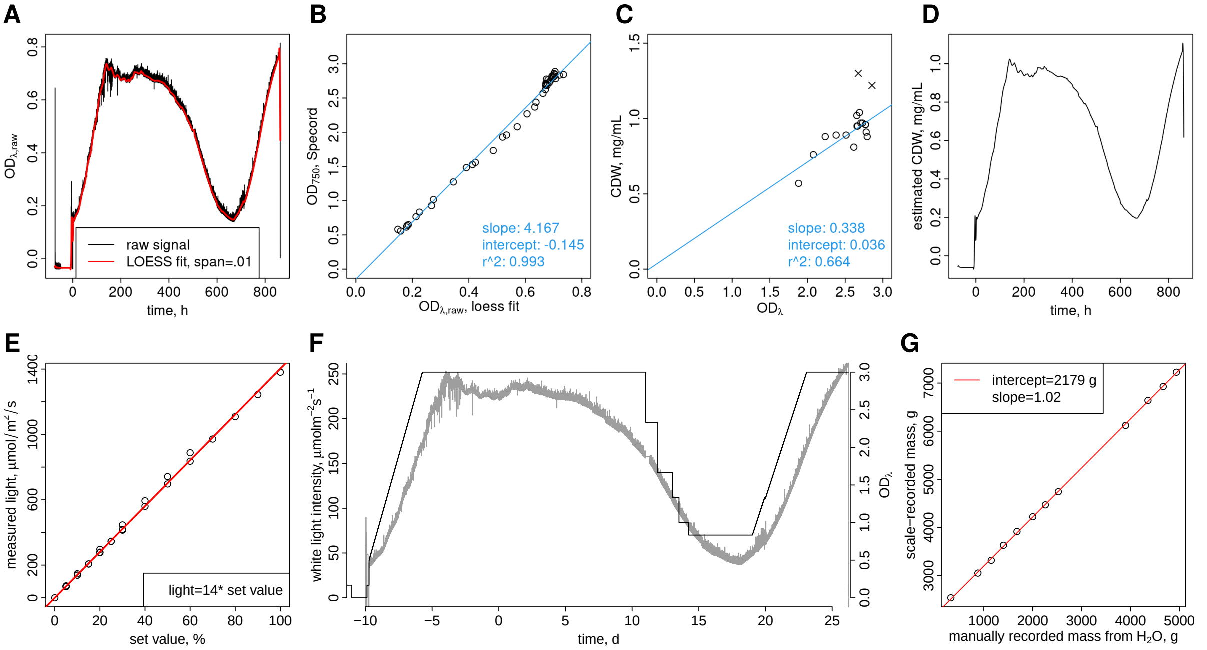

Figure A2. Calibrations. **A:** LOESS regression (R `loess`) of the raw signal (resolution ca. 1 sec) from the DASGIP OD4 module ($OD_{\lambda,raw}$). **B:** calibration of the LOESS fit of the $OD_{\lambda,raw}$ signal to offline $OD_{750\text{ nm}}$ by linear regression (R *lm*). The calibrated signal is used throughout the document and denoted OD_λ. **C:** calibration cell dry weight (CDW) to the OD_λ signal. Data points marked by X were removed as outliers. **D:** the LOESS fit of the OD4 signal was then used to estimate CDW for all time points. **E:** calibration of the Lambda LUMO light module with a Licor light meter (LI-250A) with a spherical sensor bulb (LI-193). **F:** time-series of set and calibrated (white) light intensities (black line, left y-axis) compared to the OD_λ time-series (gray line, right axis). The light intensity was manually adjusted to avoid high-light stress in the culture during biomass decrease: light was initially increased as a ramp from 42 to 250 photons, then kept constant, and manually decreased to maintain light intensity approximately at $\sim$90 µmol m^{-2} s^{-1} per OD_{750}. After the switch to batch culture light was again increased from 70 to 250 µmol m^{-2} s^{-1}. **G:** The Arduino-based scales where calibrated prior to the experiment (not shown). During the experiment the liquid level on the 5L feed bottle was marked regularly, and the mass of water filled to these marked was recorded on a benchtop scale (Kern) after the experiment to test consistent performance. The recorded mass was reproduced sufficiently well (red line: linear regression): the intercept of the linear regression corresponds to the mass of the empty feed bottle and the slope was ≈ 1. Since the manual marks on the bottle are more error prone than the pre-calibration, we did not re-calibrate the data but relied on the recorded mass for calculation of the dilution rate.

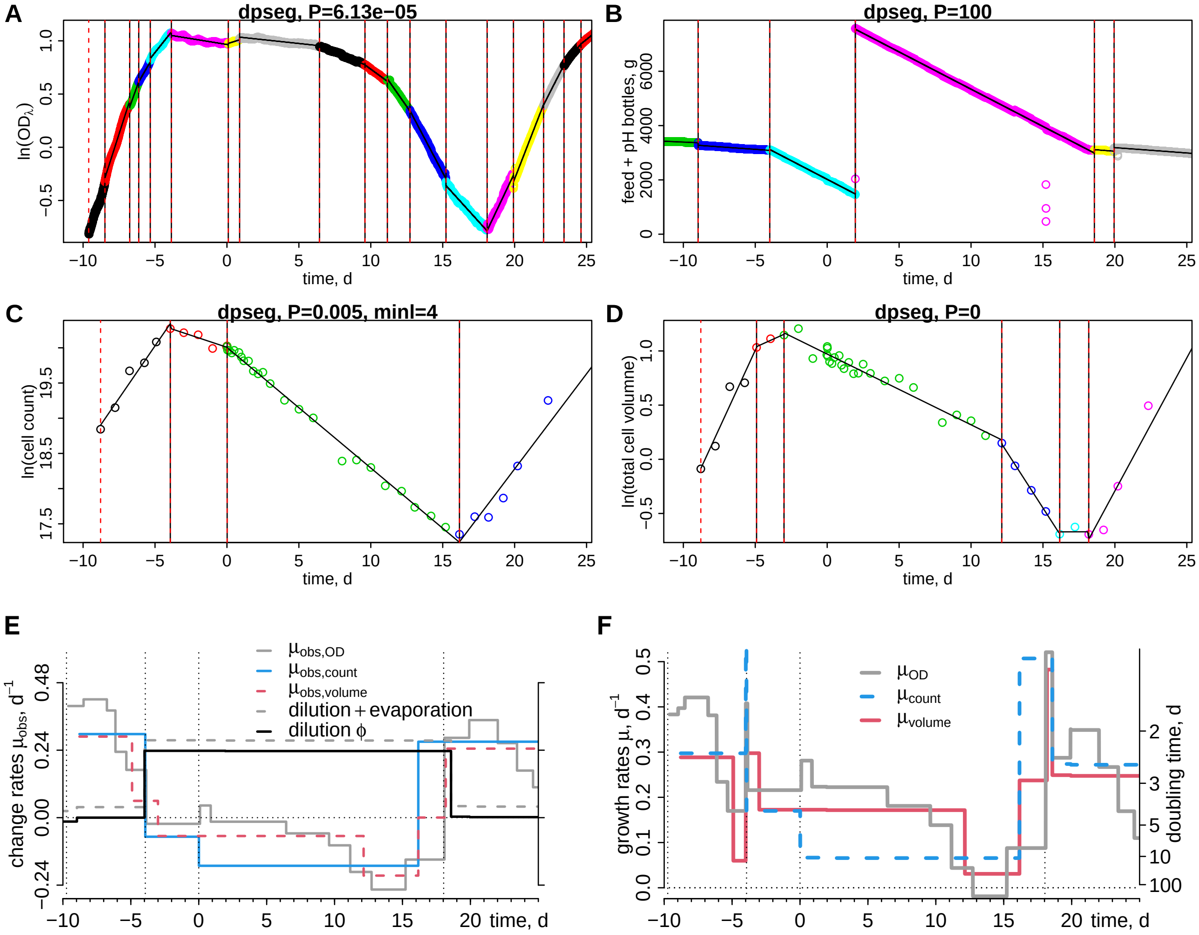

Figure A3. Calculation of Dilution and Growth Rates. All rates were calculated from the slopes of measured data (or of their natural logarithms as indicated) using piecewise linear segmentation with the R package `dpseg`. The plots in **A-D** were generated by `dpseg` and the vertical lines indicate borders of the piecewise segments, and the used penalty parameter P is shown in the plot title on the top axis. The minimal segment length parameter `minl` was only used in (C). **A**: the calibrated OD$_\lambda$ signal (1 sec resolution) was smoothed with a moving average and window size 15 and interpolated at 300 sec intervals. **B**: sum of the recorded weights of medium feed and pH control bottle weight; outliers (faulty measurements or bottle changes) were removed and data interpolated at 300 sec intervals. **C:** the total cell count for each CASY measurement, single measurements and means of technical duplicates. **D:** the total cell volume, calculated as the integral of the single cell volume distribution, for each CASY measurement, single measurements and means of technical duplicates. **E:** Observed rates. The (negative) slopes of the summed bottle weight changes (B) reflect the amounts added to the reactor culture by the Lambda reactor mass control system, assuming 1 g/mL density. The total culture dilution rate (dashed gray line, "dilution + evaporation") is obtained by division by the culture volume ($V_\ell = 1\,\text{L}$). The liquid loss by evaporation is seen at times before onset of continuous culture (time -4 d) and is subtracted to obtain the actual dilution rate ϕ (black line). The slopes of the change of the natural logarithms of the OD$_\lambda$ signal (A), the total integrated cell volume (B), and the cell counts (C) are the observed change rates $\mu_{\text{obs,OD}}$ (gray line), $\mu_{\text{obs,volume}}$ (red line) and $\mu_{\text{obs,count}}$ (blue line), respectively. **F:** The culture growth rates μ_{OD} (gray line) and μ_{count} (blue line) and μ_{volume} (red line) were calculated as the difference between observed change rates and the culture dilution rate: $\mu = \mu_{\text{obs}} - \phi$.

References

1. Yao, L., Cengic, I., Anfelt, J., and Hudson, E. (Mar, 2016) Multiple Gene Repression in Cyanobacteria Using CRISPRi.. *ACS Synth Biol,* **5**(3), 207–212.
2. Behle, A., Saake, P., Germann, A., Dienst, D., and Axmann, I. (Apr, 2020) Comparative Dose-Response Analysis of Inducible Promoters in Cyanobacteria.. *ACS Synth Biol,* **9**(4), 843–855.
3. Labun, K., Montague, T., Krause, M., Torres Cleuren, Y., Tjeldnes, H., and Valen, E. (Jul, 2019) CHOPCHOP v3: expanding the CRISPR web toolbox beyond genome editing.. *Nucleic Acids Res,* **47**(W1), W171–W174.
4. Bae, S., Park, J., and Kim, J. (May, 2014) Cas-OFFinder: a fast and versatile algorithm that searches for potential off-target sites of Cas9 RNA-guided endonucleases.. *Bioinformatics,* **30**(10), 1473–1475.
5. Cui, L., Vigouroux, A., Rousset, F., Varet, H., Khanna, V., and Bikard, D. (May, 2018) A CRISPRi screen in *E. coli* reveals sequence-specific toxicity of dCas9.. *Nat Commun,* **9**(1), 1912.
6. Pinto, F., Pacheco, C., Ferreira, D., Moradas-Ferreira, P., and Tamagnini, P. (2012) Selection of suitable reference genes for RT-qPCR analyses in cyanobacteria.. *PLoS One,* **7**(4), e34983.
7. Rüdiger, S., Rediger, A., Kölsch, A., Dienst, D., Axmann, I. M., and Machné, R. (2021) Plasmid supercoiling decreases during the dark phase in cyanobacteria: a clarification of the interpretation of chloroquine-agarose gels.. *bioRxiv,*.
8. Vischer, N., Verheul, J., Postma, M., van den Berg van Saparoea, B., Galli, E., Natale, P., Gerdes, K., Luirink, J., Vollmer, W., Vicente, M., and den Blaauwen, T. (2015) Cell age dependent concentration of *Escherichia coli* divisome proteins analyzed with ImageJ and ObjectJ.. *Front Microbiol,* **6**, 586.
9. Foley, J. bioanalyzeR: Analysis of Agilent electrophoresis data (2021) R package version 0.7.3.
10. Lehmann, R., Machné, R., Georg, J., Benary, M., Axmann, I. M., and Steuer, R. (Apr, 2013) How cyanobacteria pose new problems to old methods: challenges in microarray time series analysis.. *BMC Bioinformatics,* **14**(1), 133.
11. Lo, K., Hahne, F., Brinkman, R., and Gottardo, R. (2009) flowClust: a Bioconductor package for automated gating of flow cytometry data.. *BMC Bioinformatics,* **10**, 145.
12. Kopf, M., Klahn, S., Scholz, I., Matthiessen, J., Hess, W., and Voss, B. (Oct, 2014) Comparative analysis of the primary transcriptome of *Synechocystis* sp. PCC 6803.. *DNA Res,* **21**(5), 527–539.
13. Prakash, J., Sinetova, M., Zorina, A., Kupriyanova, E., Suzuki, I., Murata, N., and Los, D. (Dec, 2009) DNA supercoiling regulates the stress-inducible expression of genes in the cyanobacterium *Synechocystis*.. *Mol Biosyst,* **5**(12), 1904–1912.
14. Zavrel, T., Faizi, M., Loureiro, C., Poschmann, G., Stuhler, K., Sinetova, M., Zorina, A., Steuer, R., and Cerveny, J. (Feb, 2019) Quantitative insights into the cyanobacterial cell economy.. *eLife,* **8**, e42508.
15. Lehmann, R., Machné, R., and Herzel, H. (Oct, 2014) The structural code of cyanobacterial genomes.. *Nucleic Acids Res,* **42**(14), 8873–8883.
16. Saha, R., Liu, D., Hoynes-O'Connor, A., Liberton, M., Yu, J., Bhattacharyya-Pakrasi, M., Balassy, A., Zhang, F., Moon, T., Maranas, C., and Pakrasi, H. (2016) Diurnal Regulation of Cellular Processes in the Cyanobacterium *Synechocystis* sp. Strain PCC 6803: Insights from Transcriptomic, Fluxomic, and Physiological Analyses.. *mBio,* **7**(3), e00464–16.
17. Nettling, M., Treutler, H., Grau, J., Keilwagen, J., Posch, S., and Grosse, I. (Nov, 2015) DiffLogo: a comparative visualization of sequence motifs.. *BMC Bioinformatics,* **16**, 387.
18. Schieg, P. and Herzel, H. (Oct, 2004) Periodicities of 10-11bp as indicators of the supercoiled state of genomic DNA.. *J Mol Biol,* **343**(4), 891–901.
19. Schneider, T. and Stephens, R. (Oct, 1990) Sequence logos: a new way to display consensus sequences.. *Nucleic Acids Res,* **18**(20), 6097–6100.
20. Vogel, J., Axmann, I., Herzel, H., and Hess, W. (Jun, 2003) Experimental and computational analysis of transcriptional start sites in the cyanobacterium *Prochlorococcus* MED4.. *Nucleic Acids Res,* **31**(11), 2890–2899.
21. Mitschke, J., Georg, J., Scholz, I., Sharma, C., Dienst, D., Bantscheff, J., Voss, B., Steglich, C., Wilde, A., Vogel, J., and Hess, W. (Feb, 2011) An experimentally anchored map of transcriptional start sites in the model cyanobacterium *Synechocystis sp.* PCC6803.. *Proc Natl Acad Sci U S A,* **108**(5), 2124–2129.
22. Gunnelius, L., Hakkila, K., Kurkela, J., Wada, H., Tyystjarvi, E., and Tyystjarvi, T. (Apr, 2014) The omega subunit of the RNA polymerase core directs transcription efficiency in cyanobacteria.. *Nucleic Acids Res,* **42**(7), 4606–4614.
23. Koskinen, S., Hakkila, K., Kurkela, J., Tyystjarvi, E., and Tyystjarvi, T. (Jul, 2018) Inactivation of group 2 sigma factors upregulates production of transcription and translation machineries in the cyanobacterium *Synechocystis* sp. PCC 6803.. *Sci Rep,* **8**(1), 10305.
24. Kariyazono, R. and Osanai, T. (Apr, 2022) Identification of the genome-wide distribution of cyanobacterial group-2 sigma factor SigE, accountable for its regulon.. *Plant J,* **110**(2), 548–561.
25. Shultzaberger, R., Chen, Z., Lewis, K., and Schneider, T. (2007) Anatomy of *Escherichia coli* sigma70 promoters.. *Nucleic Acids Res,* **35**(3), 771–788.
26. Feklistov, A. and Darst, S. (Dec, 2011) Structural basis for promoter-10 element recognition by the bacterial RNA polymerase sigma subunit.. *Cell,* **147**(6), 1257–1269.
27. Chen, J., Chiu, C., Gopalkrishnan, S., Chen, A., Olinares, P., Saecker, R., Winkelman, J., Maloney, M., Chait, B., Ross, W., Gourse, R., Campbell, E., and Darst, S. (Apr, 2020) Stepwise Promoter Melting by Bacterial RNA Polymerase.. *Mol Cell,* **78**(2), 275–288.e6.

28. Imamura, S. and Asayama, M. (2009) Sigma factors for cyanobacterial transcription.. *Gene Regul Syst Bio,* **3**, 65–87.
29. Imamura, S., Yoshihara, S., Nakano, S., Shiozaki, N., Yamada, A., Tanaka, K., Takahashi, H., Asayama, M., and Shirai, M. (Jan, 2003) Purification, characterization, and gene expression of all sigma factors of RNA polymerase in a cyanobacterium.. *J Mol Biol,* **325**(5), 857–872.
30. Imamura, S., Asayama, M., Takahashi, H., Tanaka, K., Takahashi, H., and Shirai, M. (Nov, 2003) Antagonistic dark/light-induced SigB/SigD, group 2 sigma factors, expression through redox potential and their roles in cyanobacteria.. *FEBS Lett,* **554**(3), 357–362.
31. Kucho, K., Okamoto, K., Tsuchiya, Y., Nomura, S., Nango, M., Kanehisa, M., and Ishiura, M. (Mar, 2005) Global analysis of circadian expression in the cyanobacterium *Synechocystis* sp. strain PCC 6803.. *J Bacteriol,* **187**(6), 2190–2199.
32. Yoshimura, T., Imamura, S., Tanaka, K., Shirai, M., and Asayama, M. (Apr, 2007) Cooperation of group 2 sigma factors, SigD and SigE for light-induced transcription in the cyanobacterium *Synechocystis* sp. PCC 6803.. *FEBS Lett,* **581**(7), 1495–1500.
33. Osanai, T., Kanesaki, Y., Nakano, T., Takahashi, H., Asayama, M., Shirai, M., Kanehisa, M., Suzuki, I., Murata, N., and Tanaka, K. (Sep, 2005) Positive regulation of sugar catabolic pathways in the cyanobacterium *Synechocystis* sp. PCC 6803 by the group 2 sigma factor sigE.. *J Biol Chem,* **280**(35), 30653–30659.
34. Haugen, S., Berkmen, M., Ross, W., Gaal, T., Ward, C., and Gourse, R. (Jun, 2006) rRNA promoter regulation by nonoptimal binding of sigma region 1.2: an additional recognition element for RNA polymerase.. *Cell,* **125**(6), 1069–1082.
35. Huckauf, J., Nomura, C., Forchhammer, K., and Hagemann, M. (Nov, 2000) Stress responses of *Synechocystis* sp. strain PCC 6803 mutants impaired in genes encoding putative alternative sigma factors.. *Microbiology (Reading),* **146 (Pt 11)**, 2877–2889.
36. Zhang, X., Chen, G., Qin, C., Wang, Y., and Wei, D. (Nov, 2012) Slr0643, an S2P homologue, is essential for acid acclimation in the cyanobacterium *Synechocystis* sp. PCC 6803.. *Microbiology (Reading),* **158**(Pt 11), 2765–2780.
37. Srivastava, A., Summers, M., and Sobotka, R. (May, 2020) Cyanobacterial sigma factors: Current and future applications for biotechnological advances.. *Biotechnol Adv,* **40**, 107517.
38. Zimmerman, S. and Trach, S. (Dec, 1991) Estimation of macromolecule concentrations and excluded volume effects for the cytoplasm of *Escherichia coli*.. *J Mol Biol,* **222**(3), 599–620.
39. Oldewurtel, E., Kitahara, Y., and van Teeffelen, S. (Aug, 2021) Robust surface-to-mass coupling and turgor-dependent cell width determine bacterial dry-mass density.. *Proc Natl Acad Sci U S A,* **118**(32), e2021416118.
